# IL‐12 and IL‐15 induce the expression of CXCR6 and CD49a on peripheral natural killer cells

**DOI:** 10.1002/iid3.190

**Published:** 2017-09-27

**Authors:** Theresa Hydes, Angela Noll, Gabriela Salinas‐Riester, Mohammed Abuhilal, Thomas Armstrong, Zaed Hamady, John Primrose, Arjun Takhar, Lutz Walter, Salim I. Khakoo

**Affiliations:** ^1^ Clinical and Experimental Sciences, Faculty of Medicine University of Southampton Southampton UK; ^2^ Primate Genetics Laboratory German Primate Centre Göttingen Germany; ^3^ Transcriptome and Genome Analysis Laboratory Göttingen University Medical Centre Göttingen Germany; ^4^ Hepatobiliary Surgery University Hospital Southampton NHS Foundation Trust Southampton UK

**Keywords:** CD49a antigen, chemokine receptor 6 protein, cytokines, human liver, natural killer cells

## Abstract

**Introduction:**

Murine hepatic NK cells exhibit adaptive features, with liver‐specific adhesion molecules CXCR6 and CD49a acting as surface markers.

**Methods:**

We investigated human liver‐resident CXCR6+ and CD49a+ NK cells using RNA sequencing, flow cytometry, and functional analysis. We further assessed the role of cytokines in generating NK cells with these phenotypes from the peripheral blood.

**Results:**

Hepatic CD49a+ NK cells could be induced using cytokines and produce high quantities of IFNγ and TNFα, in contrast to hepatic CXCR6+ NK cells. RNA sequencing of liver‐resident CXCR6+ NK cells confirmed a tolerant immature phenotype with reduced expression of markers associated with maturity and cytotoxicity. Liver‐resident double‐positive CXCR6 + CD49a+ hepatic NK cells are immature but maintain high expression of Th1 cytokines as observed for single‐positive CD49a+ NK cells. We show that stimulation with activating cytokines can readily induce upregulation of both CD49a and CXCR6 on NK cells in the peripheral blood. In particular, IL‐12 and IL‐15 can generate CXCR6 + CD49a+ NK cells in vitro from NK cells isolated from the peripheral blood, with comparable phenotypic and functional features to liver‐resident CD49a+ NK cells, including enhanced IFNγ and NKG2C expression.

**Conclusion:**

IL‐12 and IL‐15 may be key for generating NK cells with a tissue‐homing phenotype and strong Th1 cytokine profile in the blood, and links peripheral activation of NK cells with tissue‐homing. These findings may have important therapeutic implications for immunotherapy of chronic liver disease.

## Introduction

Natural Killer (NK) cells provide first line defence against virally‐infected and cancer cells. They comprise nearly 50% of the hepatic lymphocyte population 1, 2 and play a role in the pathogenesis of several liver diseases. Polymorphisms within the NK cell Killer‐cell Immunoglobulin‐like Receptors (KIR) and their Human leukocyte antigen (HLA) ligands are known to influence outcomes for Hepatitis B and C viral infections and susceptibility toward hepatocellular carcinoma 3, 4, 5, 6, 7. Skewed NK cell phenotypes can also influence disease progression. In hepatitis C, enrichment of NKp46+ NK cells in the liver and hypofuctional CD56‐CD16+ NK cells in the blood, are associated with poor treatment responses 8, 9. Furthermore NK cell cytotoxicity, cytokine release, and tumor surveillance are impaired in pre‐cancerous fibrosis and cirrhosis and tumor‐infiltrating regions of the liver 10. The induction of “hyperfunctional” NK cell phenotypes in the liver may therefore improve outcomes in liver diseases.

While NK cells are classically members of the innate immune system, selected populations in mice and macaques display antigen‐specific memory toward haptens 11, 12, 13 and viral antigens 11, 14, 15, 16. The chemokine receptor (CXCR), CXCR6 12, and adhesion molecule CD49a 13 have been identified as surface markers of memory NK cells in mice. Interestingly these features were generally limited to liver‐resident NK cells. NK subsets expressing both CD49a 17 and CXCR6 18, 19, 20 have since been identified in the human liver and are generally absent from peripheral blood. CD49a+ NK cells have been described as T‐bet + Eomes−. The majority are NKG2C+ with an oligoclonal KIR expression pattern consistent with previous clonal expansion. They also have strong proliferative capabilities and have therefore been described as having adaptive features. Liver‐resident CD49a+ NK cells highly express Th1 cytokines, but show poor degranulation 17. Conversely CXCR6+ NK cells are T‐bet^low^Eomes^high^ with poor production of inflammatory cytokines and cytotoxic mediators 20. CXCR6+ NK cells are a major liver‐resident NK cell population, comprising nearly 60% of hepatic NK cells 20. They express CC chemokine receptor (CCR) 5 and may play a role in liver‐homing through their interaction with CC chemokine ligand (CCL) 3, CCL5, and CXCL16 18. CXCR6+ NK cells appear to be immunotolerant, with reduced production of interferon gamma (IFNγ), tumor necrosis factor alpha (TNFα), perforin, and granyme B 20. CXCR6 + CD69+ NK cells have also recently been described in human lymphoid tissue and therefore CXCR6 is likely to be a marker of general tissue‐residency 21. In summary, liver‐resident CD49a+ and CXCR6+ NK subsets appear distinct. There has been no direct comparison of these two subsets within the same cohort.

While antigen‐specific memory has not been demonstrated in human NK cells, many studies have shown they possess adaptive features. Clonal expansion of NKG2C+ NK cells in the peripheral blood has been seen following viral infection, particularly cytomegalovirus infection (CMV) 22, 23, 24. Furthermore NK cells in mice and humans display adaptive behavior in response to a combination of pro‐inflammatory cytokines, interleukin (IL)‐12, IL‐15, and IL‐18 25, 26, 27. Cytokines “prime” NK cells resulting in enhanced IFNγ release on re‐stimulation, enhanced proliferation 25, 26, and longevity 25.

The unique hepatic cytokine microenvironment may drive NK cell differentiation toward functionally distinct liver‐resident subsets 28, 29. This may promote tolerance in health in the face of large volumes of non‐self antigens from the portal vein, and an activated “hyperfunctional” phenotype during disease. It is therefore important to understand how NK cells expressing tissue‐resident markers CXCR6+ and CD49a+ are influenced by cytokines as this may impact liver disease. Cytokine‐induced memory‐like NK cells have already demonstrated therapeutic benefit for haematological malignancies 27, 30 and the presence of these in the liver may open doors for novel immunological therapies for viral hepatitis and liver cancer.

We therefore performed a direct phenotypic and functional comparison of liver‐resident CXCR6+ and CD49a+ NK cells in humans and assessed the role of cytokines in generating NK cells with this phenotype in both the liver and peripheral blood.

## Results

### CD49a+ NK cells are found in a small percentage of the population, whereas CXCR6+ cells are found universally

**Figure 1:**
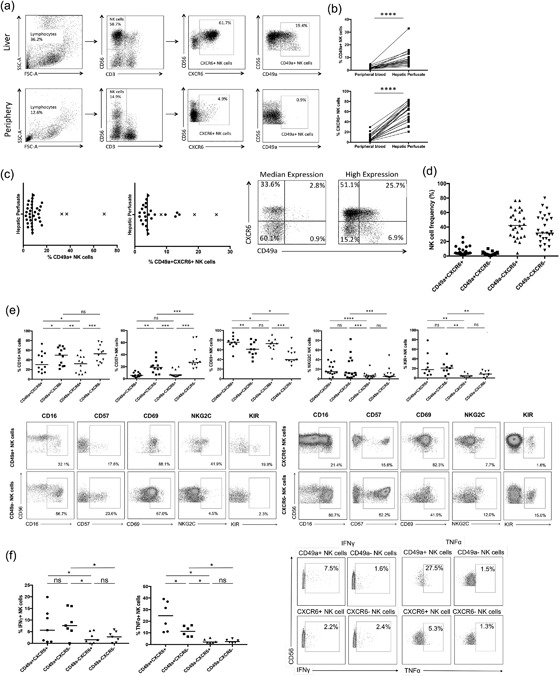
(a) Representative flow cytometry plots showing gating strategy and individual frequencies of CD49a+ and CXCR6+ NK cell populations within the peripheral blood and hepatic perfusate. (b) A comparison of the frequency of CD49a+ (*n* = 20) and CXCR6+ (*n* = 22) NK cells within the peripheral blood and hepatic perfusate (paired samples). Dot plots display individual values. (Wilcoxon matched pairs test). (c) Distribution of frequencies of CD49a+ (*n* = 35) and CD49a + CXCR6+ (*n* = 27) NK cells within the hepatic lymphocyte population. Dot plot displays individual values and median. Individuals with high frequencies of CD49a+ NK cells are plotted with a cross. Representative flow cytometry plots gated on NK cells showing examples of individuals with average and high frequencies of CD49a + CXCR6+ NK cells. (d) Frequencies of CD49a + CXCR6+, CD49a + CXCR6−, CD49a‐CXCR6+, and CD49a‐CXCR6− NK cell subsets in the human liver (*n* = 27). Dot plots display individual values and median. (e) Comparison of frequency of CD16 (*n* = 12), CD57 (*n* = 12), CD69 (*n* = 11), NKG2C (*n* = 22), and KIR+ (*n* = 9) NK cells between liver‐resident subpopulations CD49a + CXCR6+, CD49a + CXCR6−, CD49a‐CXCR6+, and CD49a‐CXCR6− (Wilcoxon matched pairs test). Representative flow cytometry plots gated on CD49a± and CXCR6± NK cells showing expression of CD16, CD57, CD69, NKG2C, and KIR. (f) Percentage of IFNγ+ (*n* = 7) and TNFα+ (*n* = 6) NK cells within the hepatic CD49a + CXCR6+, CD49a + CXCR6−, CD49a‐CXCR6+, and CD49a‐CXCR6− NK cell populations following stimulation with IL‐12 10 ng/ml and IL‐15 1 ng/ml for 12 h, respectively. Dot plots display individual values and median. (Wilcoxon matched pairs test). Representative flow cytometry plots gated on CD49a± and CXCR6± NK cells showing IFNγ and TNFα production. *p *< 0.05*, *p *< 0.01**, *p *< 0.001***, *p *< 0.0001****.

Liver mononuclear cells were isolated from the unaffected liver margin of 52 patients who had undergone hepatic resection, with paired peripheral blood samples obtained for 29 individuals. We observed CXCR6+ NK cells in all individuals with a median frequency of 57.7% (range 17.4–91.1%), whereas CD49a+ NK cell frequencies were lower, median frequency 7.8% (range 2.3–69.0%) (Fig. 1a,b). Both subsets were virtually absent from the peripheral blood; 4.0% (range 0.5–29.5%) and 1.9% (range 0.3–12.0%), respectively (Fig. 1a,b). Only 3/35 individuals (8.6%) had high frequencies of hepatic CD49a+ NK cells (>30% of the overall NK cell population) (Fig. 1c), compared to 31/34 (91.%) for CXCR6+ NK cells. Thus CXCR6+ NK cells represent the dominant liver‐specific NK cell sub‐population. Only 3.9% of hepatic NK cells were CD49a + CXCR6+ “double‐positive” (range 1.5–25.7%) with a third of individuals (8/27) displaying much higher frequencies (6.3–25.7%), dictated by CD49a expression (Fig. 1c). The majority of NK cells in the human liver were CXCR6+CD49a− (42.1%) or CXCR6‐CD49a− (31.8%) (Fig. 1d). Importantly frequencies of CXCR6+ and CD49a+ NK cells were similar whether perfusion or tissue digestion isolation techniques were used (Supplementary Fig. S1).

### CD49a+ and CXCR6+ hepatic NK cells are phenotypically distinct

Both CD49a and CXCR6 are markers of adaptive NK cells in mice, therefore to determine whether CD49a+ and CXCR6+ NK cells might represent adaptive NK cells in humans, we began by comparing their expression of markers of maturity and function. Separation of hepatic NK cell into CD49a + CXCR6+, CD49a + CXCR6−, CD49a‐CXCR6+ and CD49a‐CXCR6‐ subsets demonstrated CXCR6 expression was associated with low levels of CD16 and CD57 and high levels of CD69 (Fig. 1e). Conversely CD49a+ NK cells were more likely to be NKG2C+ or KIR+ compared to CD49a‐ NK cells. Therefore, CD49a + CXCR6+ NK cells were CD69 + CD16^low^CD57^low^ with a higher frequency of KIR and NKG2C expression compared to CD49a‐ NK cells (Fig. 1e).

### CD49a+ and CXCR6+ hepatic NK cells are functionally distinct

Following stimulation with IL‐12 and IL‐15 hepatic NK cells expressing CD49a produced greater quantities of IFNγ (7.5% vs 1.6%, *p *< 0.05) and tumor necrosis factor alpha (TNFα) (22.5 vs 2.4%, *p *< 0.05) compared to CD49a− NK cells. On CD49a+ NK cells, IFNγ production remained high irrespective of CXCR6 expression, and TNFα expression was particularly high on CD49a + CXCR6+ NK cells (Fig. 1f). Thus double‐positive CD49a + CXCR6+ cells behave more like single‐positive CD49a “adaptive‐like” NK cells, than single‐positive CXCR6+ NK cells.

### Transcriptomic analysis of CXCR6+ and CXCR6− liver‐resident NK cells

**Figure 2:**
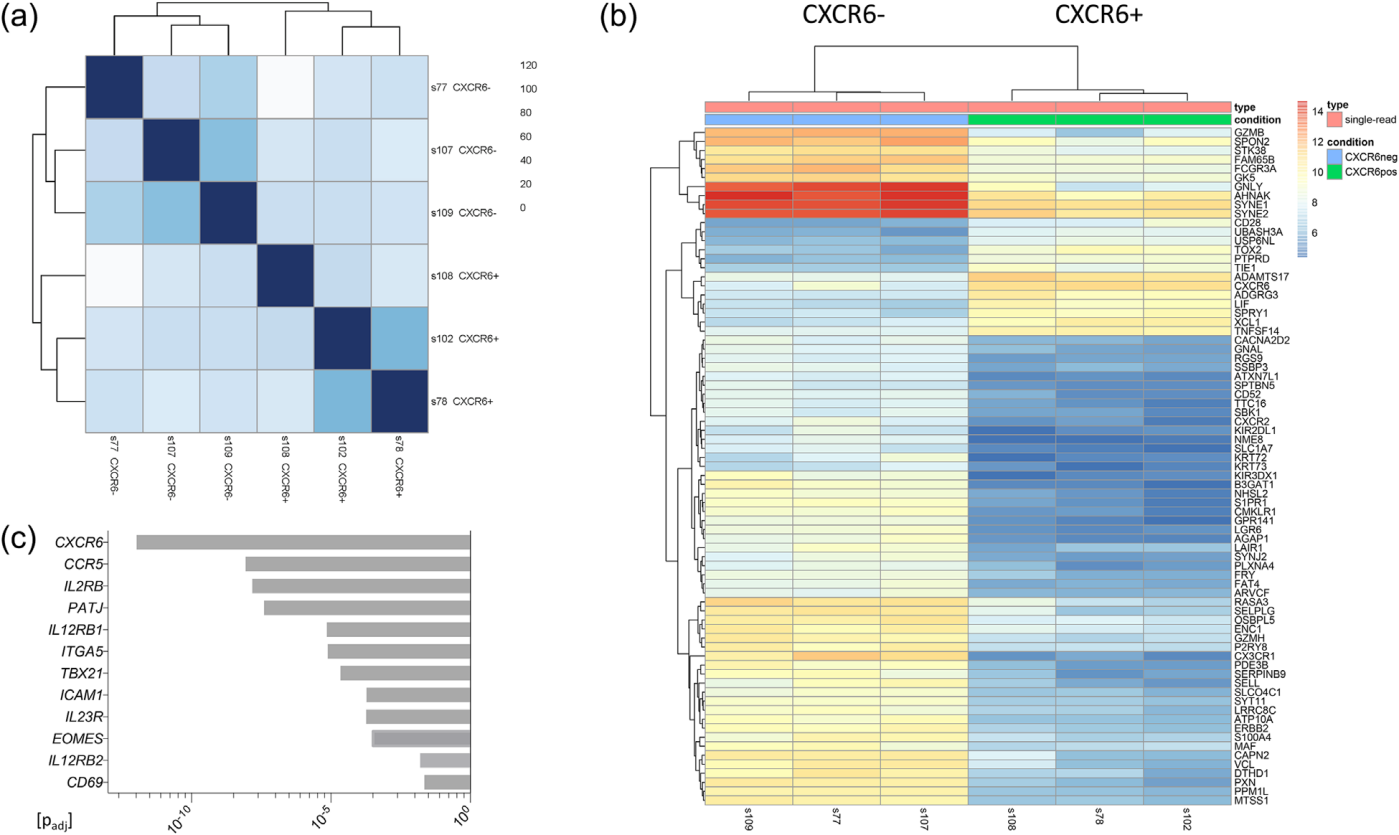
RNA‐sequencing of CXCR6+ and CXCR6‐ NK cells isolated from the liver perfusate from three patients, resulting in six paired samples (s77 + s78, s102 + s107, s108 + s109). All three patients had undergone resection for colorectal metastases and had either a normal background liver or mild steatosis. Genes with a *p* value (adjusted for multiple comparisons according to Benjamini Hochberg) of <0.05 were analysed using: (a) Euclidian distance matrix displaying the overall similarity between samples, (b) a heat map displaying the top 75 differentially expressed genes in CXCR6+ and CXCR6− NK cells, and (c) differential expression of other selected genes of interest between CXCR6+ and CXCR6− NK cells.

As hepatic CXCR6+ NK cells are known to have a distinct transcriptional profile within the liver and do not appear to be “adaptive,” we performed RNA sequencing of paired sorted liver‐resident CXCR6+ and CXCR6− NK cells from three individuals with colorectal metastases to better understand their role. Calculation of Euclidian distances revealed a close similarity among the three CXCR6‐negative samples and three CXCR6‐positive samples (Fig. 2a). We used R package DESeq2 31 to analyze the differential expression of genes between the two groups using a *p*‐value adjusted for multiple comparisons according to Benjamini Hochberg (Supplementary Table S2), and plotted the top 75 differentially expressed genes (Fig. 2b). Analysis revealed reduced expression of genes associated with maturity and cytotoxicity in liver‐resident CXCR6+ NK cells including KIR, CD16 (FCGR3A), CD57 (B3GAT1), granulysin (GNLY), granzyme B and H, and desmoyokin (AHNAK) (Fig. 2b). The latter is found on mature cytotoxic T‐cells and controls calcium signaling during cytolysis 32. CXCR6+ NK cells also displayed downregulation of signaling proteins promoting migration of lymphocytes out of tissue into the circulation (Sphingosine‐1‐phosphate receptor 1 [S1PR1], paxillin [PXN]) (Fig. 2b).

CXCR6+ NK cells expressed higher levels of Eomes and lower levels of T‐bet (TBX21) compared to CXCR6‐ NK cells (Fig. 2c) 20, 29. In terms of tissue residency they were CD69+ CD49e− (Fig. 2c) 18, 20, 33, 34. CXCR6+ NK cells showed upregulation of CCR5 which may support their migration toward, and long‐term residence in the liver (Fig. 2c) 18, 29. However CXCR2 and CX3CR1 were reduced, which code for receptors thought to be responsible for the movement of CD56^dim^ NK cells toward the liver as part of their free movement between compartments (Fig. 2b) 18. In addition CXCR6+ NK cells displayed upregulation of adhesion molecules (ICAM1, PATJ) (Fig. 2c). Finally to determine the potential for CXCR6+ liver‐resident NK cells to respond to cytokines used to generate memory‐like NK cells in the blood, we studied signaling pathways for IL‐2, IL‐12, IL‐15, and IL‐18. We observed upregulation of the IL‐23R gene, described by Cuff et al. 29, which pairs with IL‐12RB1, although the latter was down‐regulated; in addition to upregulation of IL‐12RB2 and IL‐2R (Fig. 2c). There was no consistent significant differential expression of other receptors or downstream signaling molecules within these pathways.

### Culture of hepatic MNCs with activating cytokines leads to an increase in CD49a+ NK cell frequencies, with no further enrichment of the CXCR6+ NK subset

**Figure 3:**
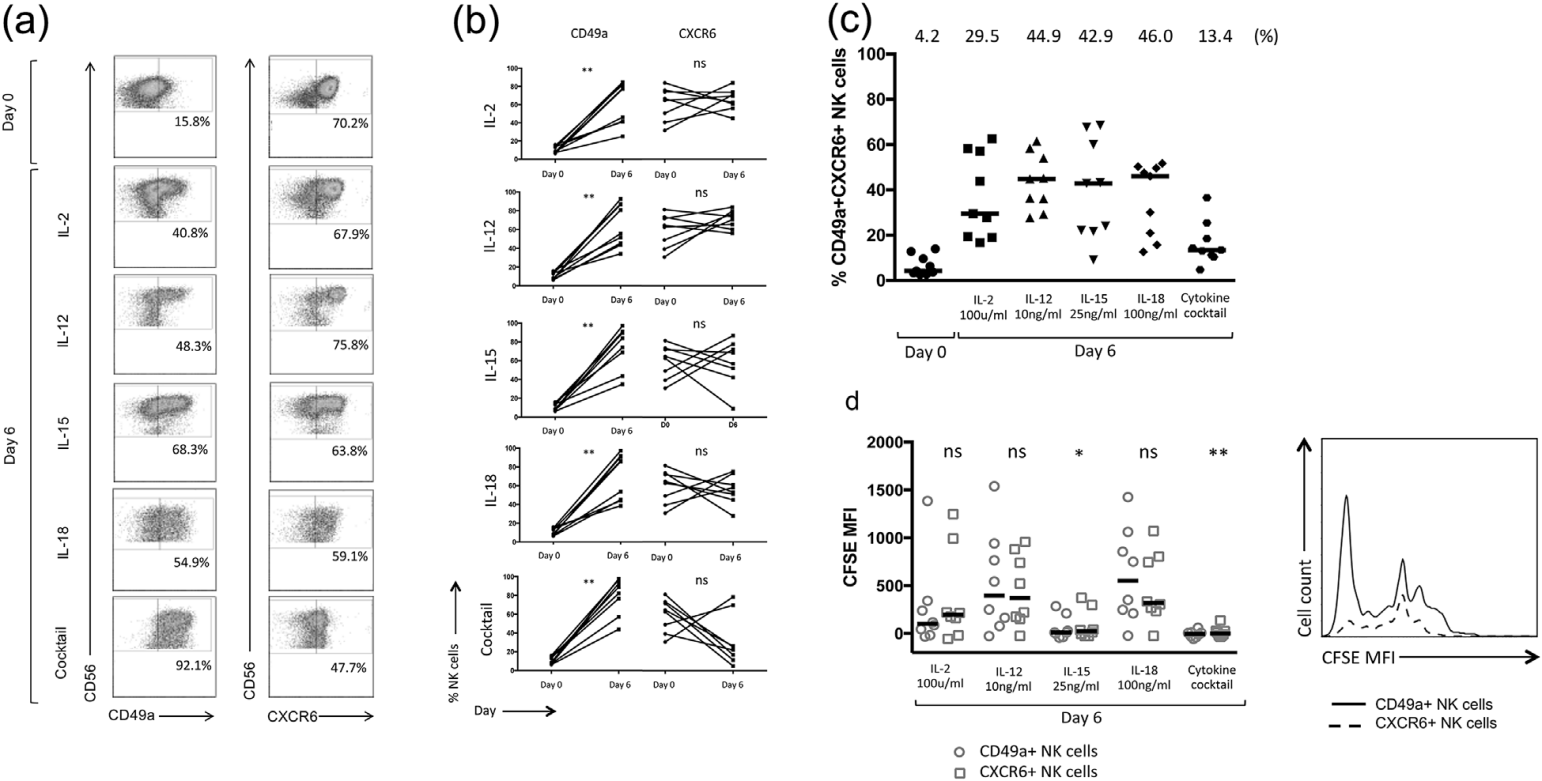
(a) Representative flow cytometry plots gated on NK cells, individual frequencies shown. (b) Percentage of CD49a+ and CXCR6+ NK cells in the peripheral blood at rest (day 0) and following incubation with IL‐2, IL‐12, IL‐15, IL‐18, and the cytokine cocktail (*n* = 8). Dot plots display individual values. (Wilcoxon matched pairs test). (c) Percentage of CD49a + CXCR6+ NK cells in the liver at rest (day 0) and following incubation with IL‐2, IL‐12, IL‐15, IL‐18, and a cytokine cocktail at day 6. Median percentages are shown. Dot plots display individual values. (d) Day 6 CFSE MFI of hepatic CD49a+ vs. CXCR6+ NK cells following culture with IL‐2, IL‐12, IL‐15, IL‐18, and the cytokine cocktail (*n* = 8). Dot plots display individual values and median (Wilcoxon matched pairs test). Representative flow cytometry histograms from one individual showing CFSE MFI of CD49a+ and CXCR6+ NK cells at day 6 following culture with IL‐15. *p *< 0.05*, *p *< 0.01**.

Having identified both CXCR6+ and CD49a+ NK cells in the human liver, we investigated their response toward activating cytokines, particularly the cytokine cocktail used to induce memory‐like NK cells in the peripheral blood. Following culture with IL‐2, IL‐12, IL‐15, IL‐18, or the cytokine cocktail (IL‐2/IL‐12/15/18) proliferating hepatic NK cells preferentially showed upregulation of CD49a rather than CXCR6 (Fig. 3a,b). Expression of CD49a on NK cells increased from 8.7% at rest to 77.1% (IL‐2), 55.7% (IL‐12), 83.9% (IL‐15), 85.7% (IL‐18), and 88.9% (cytokine cocktail). Frequencies of hepatic CXCR6+ NK cells did not increase significantly beyond their resting levels under the same conditions, with a negligible change of CXCR6 on dividing NK cells from 65.1% at day 0 to 65.5%, 64.2%, and 56.7% with IL‐2, IL‐15, and IL‐18 (Fig. 3b). IL‐12 generated the highest number of CXCR6+ NK cells by day 6 (74.1%) (Fig. 3b). Culture with the cytokine cocktail led to a decrease in the percentage of NK cells expressing CXCR6 (to 24.2% of total NK cells), in sharp contrast to its ability to upregulate CD49a (Fig. 3b).

The generation of high frequencies of CD49a+ NK cells following culture with activating cytokines, resulted in a significant increase in the percentage of double‐positive CD49a + CXCR6+ NK cells seen within the hepatic NK cell population in vitro (Fig. 3c). This was true following culture with IL‐2, IL‐12, IL‐15, and IL‐18 individually, but not the cytokine cocktail as a result of its negative influence on the frequency of CXCR6+ NK cells (Fig. 3c). Therefore, in common with our functional data, liver‐resident CD49a+ but not CXCR6+ NK cells appear to be reactive toward cytokines, particularly the cytokine cocktail.

The cytokine cocktail and IL‐15 induced the strongest proliferation of hepatic NK cells (Fig. 3d). CFSE MFI results suggested a superior proliferation of NK cells expressing CD49a over those expressing CXCR6 at day 6, following culture with IL‐15 or the cytokine cocktail. Differences between cytokines on NK cell proliferation as a whole were significantly greater than differences seen between NK cell subsets (Figs. 3d and S2). It is however not possible to conclude whether enrichment of CD49a+ NK cells within the hepatic NK cell population occurs as a result of enhanced proliferation of existing CD49a+ NK cells, or due to de novo upregulation and on previously negative cells. Marquardt et al. previously sorted these populations prior to culture with IL‐15 and feeder cells and reported an 800‐fold expansion of CD49a+ NK cells over three weeks, but also upregulation of CD49a on CD49a− NK cells suggesting both mechanisms may operate 17.

### CD49a and CXCR6 expression can be induced on peripheral blood NK cells in vitro

**Figure 4:**
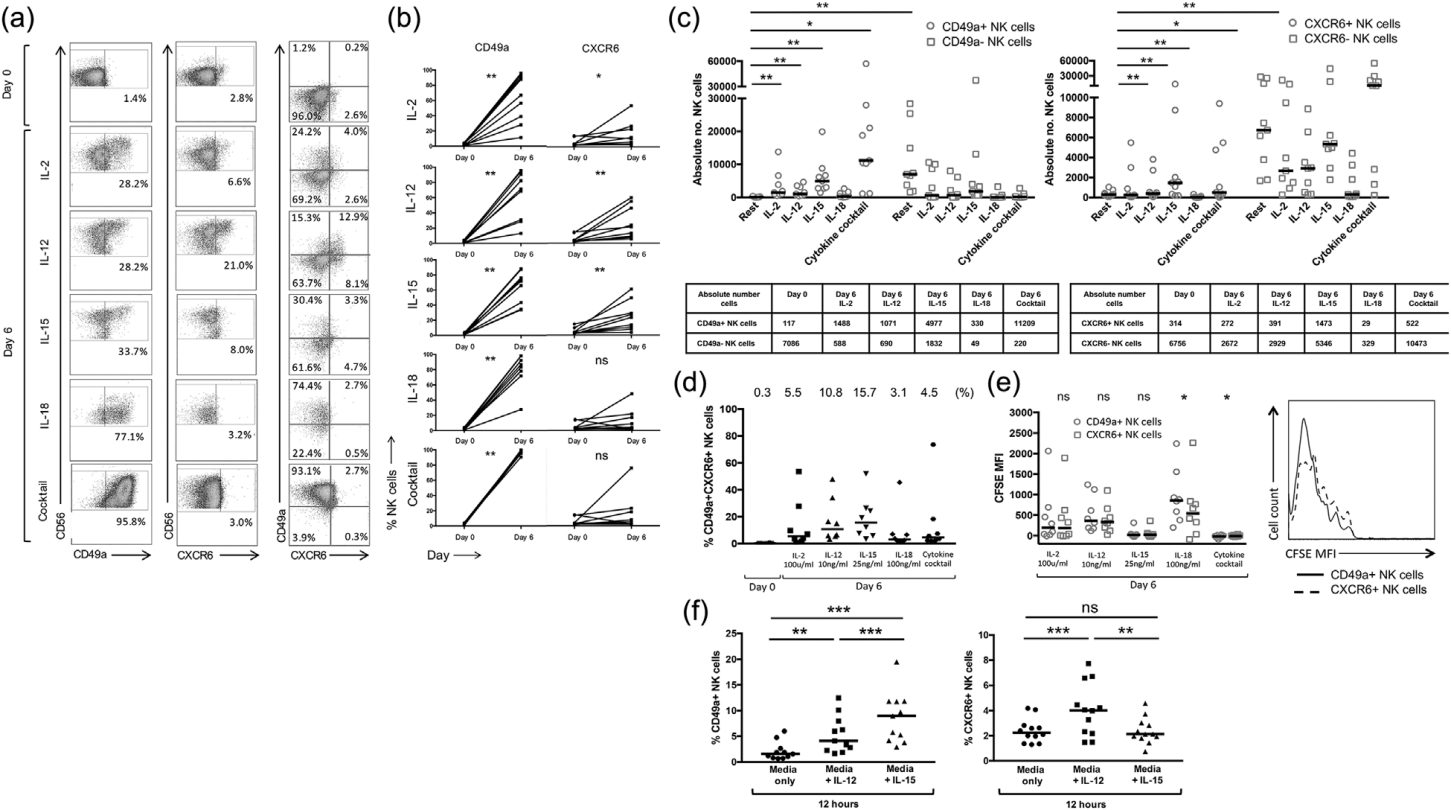
(a) Representative flow cytometry plots gated on NK cells, individual frequencies shown. (b) Percentage of CD49a+ and CXCR6+ NK cells in the peripheral blood at rest (day 0) and following incubation with IL‐2, IL‐12, IL‐15, IL‐18, and the cytokine cocktail (*n* = 9). Dot plots display individual values. (Wilcoxon matched pairs test). (c) Absolute number of CD49a± and CXCR6± NK cells at rest (day 0) and following incubation with IL‐2, IL‐12, IL‐15, IL‐18, and the cytokine cocktail. (*n* = 9). Dot plots display individual values. Median absolute cell numbers shown in table below. (d) Percentage of CD49a + CXCR6+ NK cells in the peripheral blood at rest (day 0) and following incubation with IL‐2, IL‐12, IL‐15, IL‐18, and a cytokine cocktail at day 6. Median percentages are shown. Dot plots display individual values. (e) Day six CFSE MFI of CD49a+ vs CXCR6+ NK cells in the peripheral blood following culture with IL‐2, IL‐12, IL‐15, IL‐18, and the cytokine cocktail (*n* = 8). Dot plots display individual values and median (Wilcoxon matched pairs test). Representative flow cytometry histograms from one individual showing CFSE MFI of CD49a+ and CXCR6+ NK cells at day 6 following culture with IL‐15. (f) Frequency of CD49a+ and CXCR6+ NK cells at rest and following a 12 h culture with media only, IL‐12 10 ng/ml, or IL‐15 25 ng/ml using purified NK cells (*n* = 12). Dot plots display median. (Wilcoxon matched pairs test). *p *< 0.05*, *p *< 0.01**, *p *< 0.001***.

Despite resting populations in the peripheral blood being small, cytokines were able to induce a large increase in CD49a+ NK cells frequencies from 2.1% (0.7–4.3%) to 98% (90.6–99.9%), cytokine cocktail; 83.3% (27.7–98.3%), IL‐18; 71.9% (33.9–88.3%), IL‐15; 71.3% (13.1–95.7%), IL‐12 and 66.9% (11.3–95.6%), IL‐2 (Fig. 4a,b), accompanied by an increase in absolute numbers of CD49a+ NK cells (Fig. 4c). In contrast to the liver CXCR6 could be up‐regulated on peripheral blood NK cells, with an increase in median frequency of CXCR6+ NK cells from 3.1% (1.5–14.6%) to 10.9% (2.8–54.2%), IL‐2; 21.4% (5.6–59.9%), IL‐12, and 23.3% (3.5–60.5%), IL‐15 (Fig. 4b). Frequencies remained unchanged with IL‐18 (4.1%, 1.5–48.4%) and the cytokine cocktail (5.6%, 2.1–76.1%), although there was a large degree of individual variability. As frequencies of CXCR6+ NK cells were lower at day 6 compared to CD49a+ NK cells, a significant increase in the total number of CXCR6+ NK cells in vitro could only be observed following culture with IL‐15 (associated with the highest viability of NK cells overall) (Fig. 4c). Importantly IL‐15 (15.7%) and IL‐12 (10.8%) were the most effective cytokines at inducing CD49a + CXCR6+ double‐positive NK cells compared to IL‐2 (5.5%), IL‐18 (3.1%), and the cytokine cocktail (4.5%) following 6 days of culture (Fig. 4d). In common with liver, IL‐15, and the cytokine cocktail supported the greatest proliferation of NK cells (Fig. 4e).

To examine the influence of specific cytokines on the induction of CXCR6+ and CD49a+ NK cells, we cultured purified NK cells isolated from the peripheral blood in IL‐12 and IL‐15 for 12 h. CD49a+ NK cells could be induced using both IL‐12 (4.1%) and IL‐15 (9.0%), whereas CXCR6+ NK cells could only be induced at this time point using IL‐12, from a resting frequency of 2.1–4.0% (12 h of IL‐15, frequency 2.1%) (Fig. 4f).

### Cytokine‐induced peripheral blood CD49a + CXCR6+ NK cells are CD56^bright^CD69+ with a higher frequency of NKG2C+ NK cells compared to other NK subsets

Cytokine‐induced CD49a+ NK cells generated in vitro displayed a similar phenotype to liver‐resident CD49a+ NK cells, being CD56^bright^ (68.8%) and CD69+ (83.0%) following IL‐15 stimulation. While peripheral blood CD49a− NK cells also highly expressed both markers under these conditions, CD49a+ populations contained a greater frequency of NKG2C+ NK cells than populations that remained CD49a− (37.7% vs. 19.6%, *p *< 0.01) (Supplementary Fig. S3) and CD49a+ liver‐resident NK cells (37.7% vs. 10.3%, *p *< 0.05). The majority of cytokine‐induced CXCR6+ NK cells generated in the peripheral blood were CD56^bright^ (73.3%) CD69+ (81.4%) (Supplementary Fig. S3), indicative that they display similar levels of markers of maturation and liver‐residency as those found in the liver tissue. However cytokine‐induced CXCR6+ NK cells contained higher frequencies of NKG2C+ NK cells compared to peripheral NK cells that remained CXCR6− in the presence of IL‐15 (47.5% vs. 25.0%, *p *< 0.01), and resting liver‐resident CXCR6+ NK cells (47.5% vs. 5.9%, *p *< 0.0001) (Supplementary Fig. S3). Significant differences were also seen for CD49a expression compared to peripheral NK cells that remain CXCR6− (83.0% vs. 63.0%, *p *< 0.01) and liver‐resident CXCR6+ NK cells (83.0% vs. 8.9%, *p *< 0.0001) (Supplementary Fig. S3).

**Figure 5:**
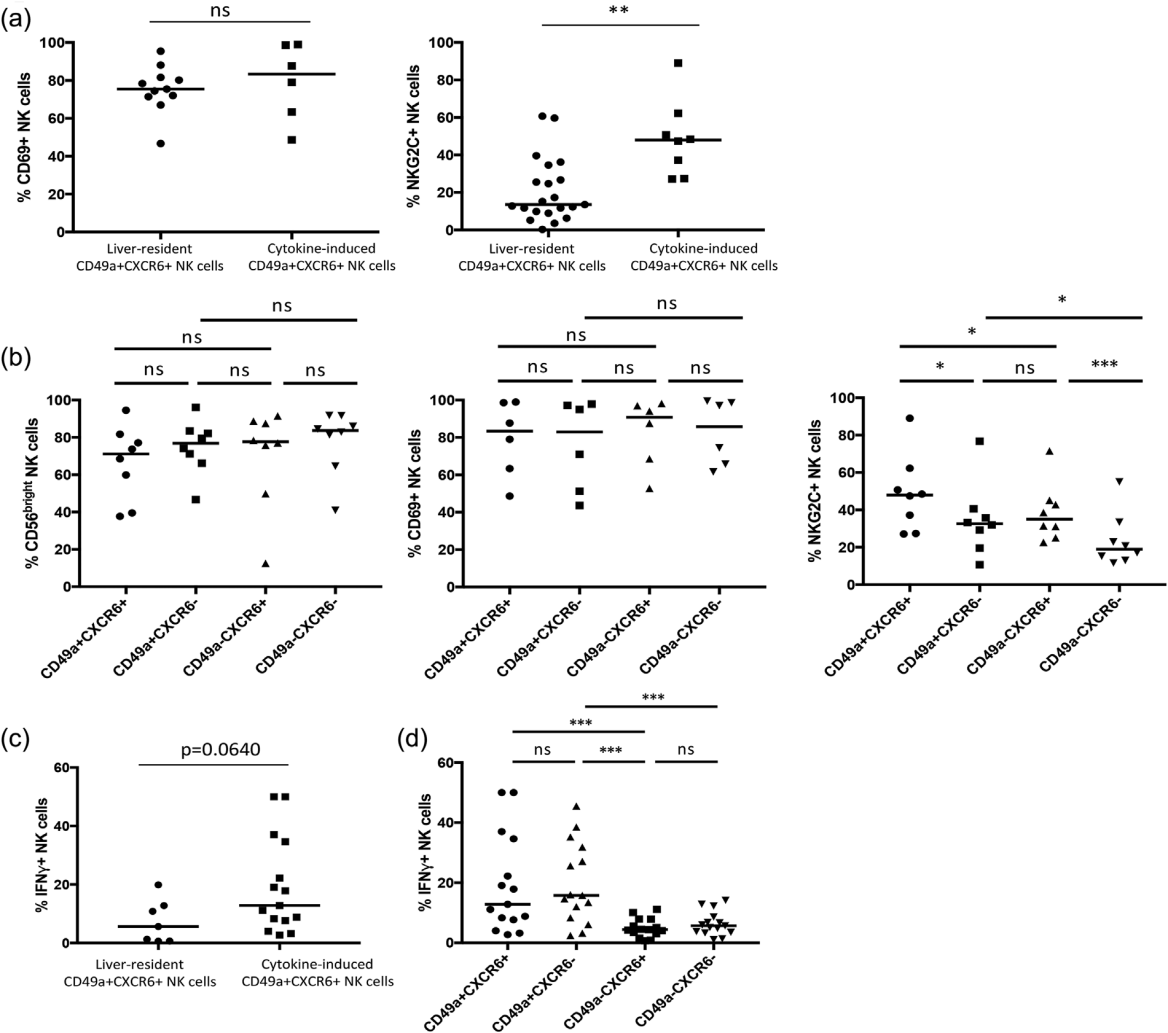
(a) A comparison of the frequencies of CD69+ (*n* = 11 liver, *n* = 6 cytokine‐induced) and NKG2C+ (*n* = 21 liver, *n* = 8 cytokine‐induced) NK cells between CD49a + CXCR6+ populations isolated from the liver and those generated in the peripheral blood following 6 days of culture with IL‐15. Dot plots display individual values and median. (Mann Whitney U test). (b) A comparison of the frequencies of CD56^bright^ (*n* = 8), CD69+ (*n* = 6), and NKG2C+ (*n* = 8) NK cells within CD49a + CXCR6+, CD49a + CXCR6−, CD49a‐CXCR6+, CD49a‐CXCR6− NK subsets generated in the peripheral blood following 6 days of culture with IL‐15. Dot plots display individual values and median. (Wilcoxon matched pairs test). (c) A comparison of the frequency of IFNγ+ NK cells (*n* = 7 liver, *n* = 15 cytokine‐induced) between CD49a + CXCR6+ populations isolated from the liver and those generated in the peripheral blood following stimulation with IL‐12 for 12 h. Dot plots display individual values and median. (Mann Whitney U test). (d) A comparison of the frequency of IFNγ+ NK cells (*n* = 15) between CD49a + CXCR6+, CD49a + CXCR6−, CD49a‐CXCR6+, CD49a‐CXCR6− NK subsets generated in the peripheral blood following stimulation with IL‐12 for 12 h. Dot plots display individual values and median. (Wilcoxon matched pairs test). *p *< 0.05*, *p *< 0.001***.

Frequencies of double‐positive CD49a + CXCR6+ NK cells were therefore higher following 6 days of cytokine stimulation of peripheral blood NK cells, than found in situ in the liver (3.9% vs. 15.7%, *p *< 0.05 [IL‐15]) (Figs. 1d and 4c). A direct comparison of the phenotype of resting liver‐resident CD49a + CXCR6+ NK cells and those generated through IL‐15 stimulation in the peripheral blood demonstrated high levels of CD69 (83.4% vs. 75.4%, *p *> 0.05), and a higher frequency of NK cells expressing the activating receptor NKG2C (48.0% vs. 13.5%, *p *< 0.001) (Fig. 5a). A similar phenotype was generated using IL‐2, IL‐12, IL‐18, and the cytokine cocktail, the latter resulting in particularly high frequencies of NKG2C+ NK cells within the CD49a + CXCR6+ population (64.2%) (Supplementary Fig. S4). Upregulation of NKG2C on cytokine‐generated CXCR6+ NK cells, was not associated with CD49a expression, as in contrast to liver NK cells, CD49a‐CXCR6+ NK cells induced by IL‐15 contained higher frequencies of NKG2C+ NK cells (35.0%) compared to CD49a‐CXCR6− NK cells (18.9%, *p *< 0.001) (Fig. 5b).

### Cytokine‐induced peripheral blood CD49a + CXCR6+ NK cells produce high quantities of IFNγ

Following 12 h culture with IL‐12 a small increase in the percentages of CD49a+ (1.5% vs. 3.3%, *p *< 0.001) and CXCR6+ (4.2% vs. 19.2%, *p *< 0.0001) NK cells from rest can be seen. Consistent with their comparable phenotype to liver‐resident CD49a + CXCR6+ NK cells, cytokine‐induced CD49a + CXCR6+ NK cells produced similar quantities of IFNγ (Fig. 5c). Cytokine‐induced peripheral blood CXCR6+ NK cells failed to produce significant quantities of IFNγ unless they co‐expressed CD49a (Fig. 5d). However after 6 days of culture, over 80% of CXCR6+ NK cells were CD49a+ (unlike in the liver) (Supplementary Fig. S3). Therefore, cytokine stimulation of peripheral blood NK cells can generate high frequencies of CD49a + CXCR6+ NK cells which are activated, express CD69 and produce high quantities of IFNγ (Fig. 5a,c).

## Discussion

We have shown that liver‐resident CXCR6+ NK cells are immature (CD56^bright^CD16^low^) and are found in all individuals, comprising nearly two thirds of hepatic NK cells. While human CXCR6+ NK cells do not display features consistent with “memory” as in mice, CXCR6 may play a role retaining NK cells within the liver via its interaction with the chemokine ligand 16 (CXCL16), expressed on sinusoidal endothelial cells, hepatocytes, and cholangiocytes 35. RNA sequencing has revealed reduced expression of markers of maturity and cytotoxicity.

Conversely, hepatic CD49a+ NK cells are only found in substantial frequencies in 10% of individuals, and may be even less frequent in a completely healthy population. The CD49a+ NK cell subset contains higher frequencies of KIR+ and NKG2C+ NK cells compared to the CD49a‐ subpopulation, suggesting some of these cells may have undergone previous clonal expansion. They also produce high quantities of IFNγ. While we did not stratify for CMV, no correlation was demonstrated between seropositivity and hepatic CD49a+ NK cell frequencies by Marqadt el at 17.

We have demonstrated that cytokines can generate hyperfunctional CD49a+ NK cells from PBMCs in vitro, with a phenotype comparable to those found in the resting liver (CD56^bright^ CD69+ IFNγ+, with higher percentages of NKG2C+ NK cells compared to CD49a− NK subsets). CD49a is also known as very late activation antigen‐1, and has previously been shown to be induced by IL‐2 36, 37. We now show that this activation is associated with enhanced functionality and a phenotype associated with liver‐residency. Therefore, potentially in addition to having an activated phentoype, cytokine‐induced CD49a+ NK cells may be adaptive, or a precursor of adaptive NK cells. Further experiments including additional stimulation would be useful to investigate this.

The cytokine cocktail led to the greatest upregulation of CD49a in both PBMCs and hepatic MNCs. This may mimic events in vivo in which an inflammatory hepatic cytokine microenvironment, found in hepatitis or cancer, dominated by IL‐1, IL‐2, IL‐6, IL‐12, IL‐15, IL‐18, IFNα, IFNγ, and TNFα/β 38, 39, may lead to the expansion of CD49a+ NK cells. Of the three patients in our cohort with significant enrichment of hepatic CD49a+ NK cells, one had hepatocellular carcinoma, one had aggressive colorectal cancer with synchronous lesions and bi‐lobar liver metastases and the third had colorectal metastases extending to the resection margins, suggesting that high frequencies of CD49a+ NK cells are associated with more severe liver disease. The cytokine hyper‐responsive behavior observed in vitro in this subset may occur as a result of previous cytokine‐priming and the adaptive qualities of liver‐resident CD49a+ NK cells may therefore be driven through similar mechanisms to cytokine‐induced memory‐like NK cells generated in the blood 26. It would be important to explore whether cytokine‐induced CD49a+ NK cells generated in the peripheral blood display memory‐like behavior similar to that demonstrated for liver‐resident CD49a+ NK cells 17, or whether CD49a is a surface markers of cytokine‐induced memory‐like NK cells described by Romee et al. 26.

We show IL‐2, IL‐12 and IL‐15 can upregulate CXCR6 on peripheral NK cells. This is likely to induce their migration toward and residence within tissues, but particularly the liver which is home to a major population of CXCR6+ NK cells and contains large numbers of CXCL16‐expressing cells 35. Both CXCR6+ and CD49a+ NK cells are associated with tissue‐residency in organs other than the liver. However, CXCR6 + CD69+ NK cells from the spleen do not express CD49a, and CD49a+ NK cells from the lung are hypofunctional 21, 40. Furthermore dynamic flow assays mimicking hepatic sinusoids have shown that a small molecule inhibitor for CXCR6 can reduce migration of NK cells across the hepatic endothelium, indicating the relevance of this chemokine receptor for migration to the liver 41. Cytokine‐induced CXCR6+ NK cells are more activated than their hepatic counterparts, in terms of NKG2C expression, cytokine‐induction, and potential to produce high quantities of IFNγ where CD49a is co‐expressed.

Interestingly, culture with the cytokine cocktail leads to a reduction in the frequencies of CXCR6+ NK cells within the hepatic NK cell population, and fails to expand the CXCR6+ population within PBMCs, in contrast to the individual influences of all four cytokines. Persistent exposure to multiple activating cytokines may lead to NK cell exhaustion and activation‐induced NK cell apoptosis. Loss of activated cells within the culture may lead to a lower than expected frequency of CXCR6+ NK cells .

We have shown through culture with IL‐12 or IL‐15 it is possible to generate high frequencies of activated double‐positive CD49a + CXCR6+ NK cells in the peripheral blood which display both markers of tissue residency (CD69) and phenotypic and functional similarities to liver‐resident adaptive‐like CD49a+ NK cells (NKG2C, IFNγ). This data also suggests cytokine signaling, in addition to CMV infection, can lead to expansion of NK cells expressing the adaptive marker NKG2C 22, 23, 24. This transition may be supported by changes at a transcription factor level, for example IL‐15 and TGFβ have recently been shown to induce transition of Eomes^low^ to Eomes^high^ NK cells 29. However, CXCR6 was not upregulated under these conditions and T‐bet is already highly expressed on the majority of CD49a− peripheral blood NK cells, suggesting other mechanisms may be important 42.

It is therefore possible that in liver disease, in addition to clonal expansion of adaptive‐like CD49a+ liver‐resident NK cells, there is hepatic recruitment of newly generated CD49a + CXCR6+ NK cells, capable of releasing high levels of Th1 cytokines, induced by systemic inflammation in the peripheral blood including high levels of IL‐12/IL‐15. This process may be driven through IFNγ upregulation of CXCL16 in the liver. These findings may have important therapeutic applications. The generation of activated CXCR6+ NK cells in the peripheral blood that co‐express CD49a and adopt an “adaptive” phenotype, may allow hyperfunctional NK cells to be preferentially recruited to the liver, boosting the hepatic innate immune response to fight viruses and cancer, providing a basis for novel, locally acting immunotherapies for common hepatic disease.

## Materials and Methods

### Patients

Patients were recruited from University Hospital Southampton NHS Foundation Trust. Liver tissue was obtained from the margin of 52 adults undergoing resection for liver metastases or primary liver cancer. Demographic data is displayed in Supplementary Table S1. Paired peripheral blood samples were obtained for 29 of the 52 patients, the remainder collected from individuals with haemochromatosis.

### Isolation of mononuclear cells from human liver tissue

Tissue was infiltrated with chelating buffer (1x Phosphate Buffered Saline [PBS] [BioWhittaker, Belgium] 50 ml, HEPES 28 mg [Sigma, Poole, UK], EGTA 9.5 mg [Sigma]) and perfusate collected. Cells were isolated from the liver parenchyma using collagenase digestion (Dulbecco's Modified Eagle Medium [DMEM] [Gibco®, Life Technologies™, UK] 50 ml, TIV Collagenase 18 mg [Sigma], calcium chloride 90 µl [Sigma]) followed by mechanical disaggregation or mechanical disaggregation alone. Hepatic mononuclear cells (MNCs) and PBMCs were isolated using Ficoll‐Paque™ density centrifugation (GE Healthcare, Sweden).

### NK cell surface staining

Hepatic MNCs and PBMCs were analyzed in parallel. Cells were incubated in Zombie Violet™ Fixable Viability Kit (Biolegend®, London, UK) for 15 min, then blocking buffer (10% human serum [HS] [Sigma] in FACS wash [PBS with 1% bovine serum albumin [BSA] [Sigma] and 0.1 % sodium azide [Sigma]) for 20 min prior to surface antibody staining: CD3 (UCHT1, PerCP, Biolegend®; UCHT1, BDV450, BD Biosciences, Oxford, UK; UCHT1, BV510, Biolegend®), CD56 (HCD56, PE‐Cy7, Biolegend®), CD16 (3G8, APC‐Cy7, Biolegend®), CD57 (HNK‐1, APC, Biolegend®; NK‐1, PE‐CF594, BD Biosciences), CD161 (DX12, BV421, BD Biosciences), CD158a (HP‐MA4, FITC, Biolegend®, KIR2DL1/S1/S3/S5), CD158b (CH‐L, FITC, BD Biosciences, KIR2DL2/3; DX27, PerCP, Miltenyi Biotec, Guildford, UK, KIR2DL2/3), NKG2C (REA205, PE, Miltenyi Biotec; REA205, ViobrightFITC, Miltenyi Biotec; REA205, APC, Miltenyi Biotec), CD49a (SR84, PE, BD Biosciences), CD49b (AK‐7, FITC, BD Biosciences), CXCR6 (K041E5, APC, Biolegend®; K041E5, PerCP/Cy5.5, Biolegend®). Cells were analyzed using a three laser FACS Aria (BD Biosciences) flow cytometer. Gates were set using fluorescence minus one (FMO) controls. Data was analysed using FlowJo v.10.0 (Treestar, Ashland, OR, USA).

### NK cell proliferation assays

Paired PBMCs and perfusate MNCs were resuspended in PBS/0.1% BSA to create a 2x cell solution. This was resuspended in Carboxyfluorecin succinimidyl ester (CFSE) staining solution (CellTrace™ CFSE Cell Proliferation Kit) (Life Technologies™, Paisely, UK) to make a final CFSE concentration of 5 μM and incubated for 10 min, 37°C. Staining was quenched with 5 volumes ice‐cold Roswell Park Memorial Institute Medium (RPMI) 1640 + Glutamax (Gibco®, Life Technologies™) supplemented with 10% fetal bovine serum (Hyclone®, Thermoscienticic, Northumberland, UK), penicillin, streptomycin and glutamine (Gibco®, Life Technologies™) (R‐10) and incubated for 5 min, 4°C. Cells were washed three times in R‐10 then recounted. PBMCs and liver MNCs were incubated in R‐10 supplemented with 5% HS (Sigma) in addition to Recombinant Human IL‐2 100 U/ml (PeproTech, London, UK), IL‐12 10 ng/ml (PeproTech), IL‐15 25 ng/ml (R&D Systems, Oxford, UK), IL‐18 100 ng/ml (Medical and Biological Laboratories, Japan), or a cocktail of all four for 6 days. Media and cytokines were changed every 2–3 days. A CFSE FMO was included. On day 0 and 6 PBMCs and liver MNCs underwent staining with Zombie Violet™ Fixable Viability Kit (Biolegend®), CD3‐BV510 (Biolegend®), CD56‐PECy7 (Biolegend®), NKG2C‐APC (Miltenyi Biotec), CXCR6‐PerCP/Cy5.5 (Biolegend®), and CD49a‐PE (BD Biosciences).

### NK cell purification and stimulation with IL‐12 and IL‐15

Freshly isolated PBMCs were counted and centrifuged at 300 g for 10 min before resuspending in NK cell isolation buffer (NKIB) (40 μl per 10^7^ cells) (250 mM EDTA 2 ml, pH 8, BSA 1.25g, 248 ml PBS) and NK cell biotin‐antibody cocktail (10 μl per 10^7^ cells) (Human NK cell isolation kit, Miltenyi Biotec) and incubated for 5 min, 4°C. Cells were resuspended in 30 μl per 10^7^ cells NKIB and NK cell micro‐bead cocktail (20 μl per 10^7^ cells) (Human NK cell isolation kit, Miltenyi Biotec) and incubated for 10 min, 4°C. The cell suspension was placed onto the LS column (Miltenyi Biotec) and unlabelled NK cells collected. The column was rinsed with a further 3 ml NKIB and cell suspension collected. Purified NK cells were cultured for 12 h in R‐10 supplemented with 5% HS alone or with IL‐12 10 ng/ml, or IL‐15 25 ng/m prior to surface staining with CD3‐BV510, CD56‐PE‐Cy7, CXCR6‐PerCP/Cy5.5, and CD49a‐PE as above.

### NK cell intracellular interferon gamma and tumor necrosis factor staining

PBMCs and liver MNCs were stimulated for 12 h with IL‐12 10 ng/ml (PeproTech) or IL‐15 1 ng/ml (R&D Systems) to examine IFNγ and TNFα production respectively. An unstimulated control was included. BD GolgiStop™ (BD Biosciences) was added (4 µl/6 ml culture medium) for the last 4 h. Surface staining was performed for CD3‐BV510 (Biolegend®), CD56‐PE‐Cy7 (Biolegend®), CXCR6‐PerCP/Cy5.5 (Biolegend®), and CD49a‐PE (BD Biosciences). Cells were fixed and permeabilised (BD Cytofix/Cytoperm™ Plus Kit, BD Biosciences) prior to incubation with IFNγ (B27, APC, Biolegend®) or TNFα (MAb11, FITC, Biolegend®).

### RNA sequencing

Hepatic CXCR6+ and CXCR6− NK cells were sorted using fluorescence‐activated cell sorting, gating on live CD3‐CD56 + CXCR6+ and CD3‐CD56 + CXCR6− lymphocytes. Total RNA was isolated using TRIzol® reagent (Life Technologies™) and digested with RNase‐Free DNase‐I. Quantity and quality of extracted RNA were analyzed using the Fragment Analyser (Advanced Analytical) and NanoDrop® ND‐1000 Spectrophotometer (Thermo Scientific NanoDrop Technologies, Wilmington, Delaware, USA). A total of 50 ng of each total RNA was used as starting material. TruSeq Stranded Total RNA Library Prep Kit with Ribo‐Zero Human/Mouse/Rat (Illumina Cat. N° RS122‐2201) was used to prepare samples. Accurate quantitation of cDNA libraries was performed using the QuantiFluorTM dsDNA System (Promega, Germany) and the size range of cDNA libraries determined using the Fragment Analyser (280 bp). cDNA libraries were amplified and sequenced using cBot and HiSeq 2000 (llumina) (SR, 50bp, ca. 30 million reads/sample). Sequence images were transformed with Illumina software BaseCaller, which were demultiplexed with CASAVA (v.1.8.2). Quality checks were performed via FastQC (Babraham Bioinformatics).

Sequenced reads were mapped against the human genome (hg38) using STAR (version 020201) 43 with parameters—outSAMtype BAM SortedByCoordinate—outFilterMismatchNmax 2. Read counts per gene were examined using featureCount (v.1.5.0‐p1) 44. Normalization of read counts to the library size, estimation of dispersions and testing for differentially expressed genes based on a statistical test assuming negative binomial data distribution were computed in the R/Bioconductor environment (v.2.15.2) loading DESeq2 (1.14.1) and biomaRt (2.14.0) packages 31, 45, 46. Significant genes were determined as log2 fold change (log2FC) <1 or > − 1, base mean <1000, and false discovery rate‐corrected *p*‐value <0.05 with multiple testing correction according to Benjamini and Hochberg. A heat map was constructed using the top 75 differetially expressed genes. Euclidian distance matrix was also calculated using DeSeq2. Data generated conformed to *MIAME* standards and was submitted to the Gene Expression Omnibus database.

### Ethical approval

Ethical approval to collect paired peripheral blood and liver tissue was granted by the Wales Research Ethics Committee (REC No. 13/WA/0329). Ethical approval to collect peripheral blood samples from haemochromatosis patients was granted by the South Central Hampshire Research Ethics Committee (REC No. 06/Q1701/120). Informed consent of all participants was obtained.

## Conflicts of Interest

The authors declare no commercial or financial conflict of interest.

## Supporting information

Additional supporting information may be found in the online version of this article at the publisher's web‐site.

**Table S1**. Patient demographic data.
**Figure S1**. (a) A comparison of the frequency of CD49a+ NK cells within the peripheral blood, hepatic perfusate, and liver parenchyma NK cell populations (paired and unpaired samples, *n *= 35, *n *= 35, *n *= 18). Dot plots display individual values and median. (Mann Whitney U test). (b) A comparison of the frequency of CD49a+ NK cells within the peripheral blood, hepatic perfusate, and liver parenchyma NK cell populations (paired and unpaired samples, *n *= 26, *n* = 34, *n *= 11). Dot plots display individual values and median. (Mann Whitney U test). *p *< 0.0001****.
**Figure S2**. (a) Day 6 CFSE MFI of hepatic NK cells following culture with IL‐2, IL‐12, IL‐15, IL‐18, and the cytokine cocktail. Median values displayed below. Dot plots display individual values and median. Representative flow cytometry histograms from one individual show CFSE expression at day 6 following culture with IL‐2, IL‐12, IL‐15, IL‐18, and the cytokine cocktail. (b) Day 6 CFSE MFI of peripheral blood NK cells following culture with IL‐2, IL‐12, IL‐15, IL‐18, and the cytokine cocktail. Median values displayed below. Median values displayed below. Dot plots display individual values and median. Representative flow cytometry histograms from one individual show CFSE MFI at day 6 following culture with IL‐2, IL‐12, IL‐15, IL‐18, and the cytokine cocktail.
**Figure S3**. (a) A comparison of CD56bright, CD69+, NKG2C+, and CXCR6+ NK cell frequencies found within CD49a+ and CD49a− NK subsets generated in the peripheral blood following 6 days of culture with IL‐15 (*n *= 9). Bar chart displays median and interquartile range. (Wilcoxon matched pairs test). (b) A comparison of CD56bright, CD69+, NKG2C+, and CD49a+ NK cell frequencies found within CXCR6+ and CXCR6− NK subsets generated in the peripheral blood following 6 days of culture with IL‐15 (*n *= 9). Bar chart displays median and interquartile range. (Wilcoxon matched pairs test). median *p *< 0.05*, *p *< 0.01**.
**Figure S4**. A comparison of CD56bright, CD69+, and NKG2C+ NK cell frequencies between CD49a+CXCR6+, CD49a+, CXCR6−, CD49a‐CXCR6+, CD49a‐CXCR6− NK subsets generated in the peripheral blood following 6 days of culture with IL‐2, IL‐12, IL‐15, IL‐18, and a cocktail of all four cytokines (*n *= 9). Bar chart displays median and interquartile range.

**Table S2**. Differential expression of genes between CXCR6+ and CXCR6‐ NK cells.
