# Supplementary material for: IL‐12 and IL‐15 induce the expression of CXCR6 and CD49a on peripheral natural killer cells
Source: Immun Inflamm Dis. 2017 Sep 27;6(1):34–46. doi: 10.1002/iid3.190 (PMC5818449; doi:10.1002/iid3.190)
Supplement: Supplementary file 1 — Table S1. Patient demographic data. Figure S1. (a) A comparison of the frequency of CD49a+ NK cells within the peripheral blood, hepatic perfusate, and liver parenchyma NK cell populations (paired and unpaired samples, n = 35, n = 35, n = 18). Dot plots display individual values and median. (Mann Whitney U test). (b) A comparison of the frequency of CD49a+ NK cells within the peripheral blood, hepatic perfusate, and liver parenchyma NK cell populations (paired and unpaired samples, n = 26, n = 34, n = 11). Dot plots display individual values and median. (Mann Whitney U test). p < 0.0001****. Figure S2. (a) Day 6 CFSE MFI of hepatic NK cells following culture with IL‐2, IL‐12, IL‐15, IL‐18, and the cytokine cocktail. Median values displayed below. Dot plots display individual values and median. Representative flow cytometry histograms from one individual show CFSE expression at day 6 following culture with IL‐2, IL‐12, IL‐15, IL‐18, and the cytokine cocktail. (b) Day 6 CFSE MFI of peripheral blood NK cells following culture with IL‐2, IL‐12, IL‐15, IL‐18, and the cytokine cocktail. Median values displayed below. Median values displayed below. Dot plots display individual values and median. Representative flow cytometry histograms from one individual show CFSE MFI at day 6 following culture with IL‐2, IL‐12, IL‐15, IL‐18, and the cytokine cocktail. Figure S3. (a) A comparison of CD56bright, CD69+, NKG2C+, and CXCR6+ NK cell frequencies found within CD49a+ and CD49a− NK subsets generated in the peripheral blood following 6 days of culture with IL‐15 (n = 9). Bar chart displays median and interquartile range. (Wilcoxon matched pairs test). (b) A comparison of CD56bright, CD69+, NKG2C+, and CD49a+ NK cell frequencies found within CXCR6+ and CXCR6− NK subsets generated in the peripheral blood following 6 days of culture with IL‐15 (n = 9). Bar chart displays median and interquartile range. (Wilcoxon matched pairs test). median p < 0.05*, p < 0.01**. Figure S4. A compariso [file IID3-6-34-s001.pdf]

Supplementary Table 1. Patient demographic data

**Demographic Details (n=52)**	
**Age, years (range)**	65 (29-80)
**Men, n (%)**	32 (61.5)
**Pre-operative chemotherapy, n (%)**	29 (55.8)
**Reason for resection, n (%)**	
Metastases, colorectal	33 (63.5)
Metastases, other	8 (15.4)
Hepatocellular carcinoma	6 (11.5)
Cholangiocarcinoma	2 (3.8)
Gastrointestinal stromal tumour	1 (1.9)
Hepatic cyst	1 (1.9)
Lymphoma	1 (1.9)
**Background liver histology, n (%)**	
Normal	30 (57.7)
Mild steatosis	16 (30.8)
Moderate steatosis	3 (5.8)
Fibrosis	3 (5.8)
**Clear resection margin**	50 (96.2)

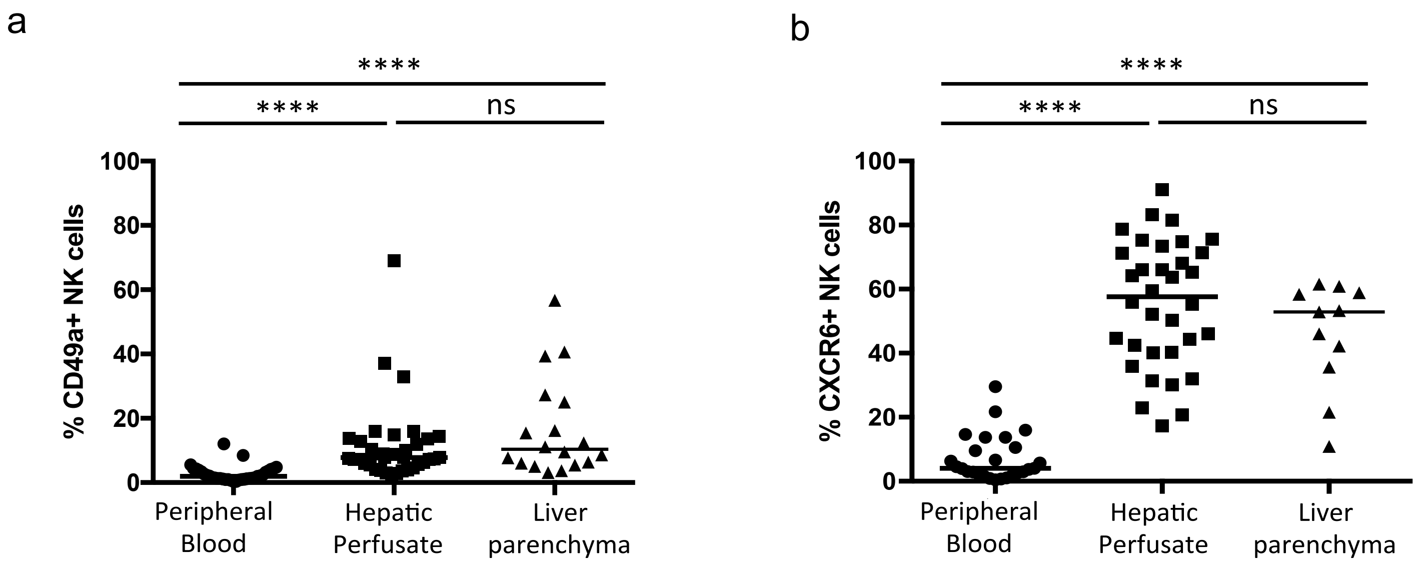

Supplementary Figure 1. a) A comparison of the frequency of CD49a+ NK cells within the peripheral blood, hepatic perfusate and liver parenchyma NK cell populations (paired and unpaired samples, n=35, n=35, n=18). Dot plots display individual values and median. (Mann Whitney U test). **b)** A comparison of the frequency of CD49a+ NK cells within the peripheral blood, hepatic perfusate and liver parenchyma NK cell populations (paired and unpaired samples, n=26, n=34, n=11). Dot plots display individual values and median. (Mann Whitney U test). p<0.0001****.

HEPATIC PERFUSATE

a

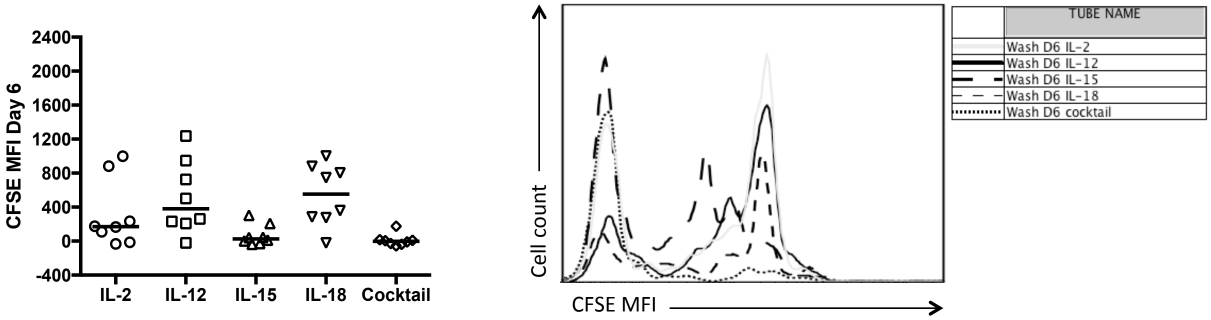

IL-2	IL-12	IL-15	IL-18	Cocktail
170	381.3	26.91	553.1	-2.58

PERIPHERAL BLOOD

b

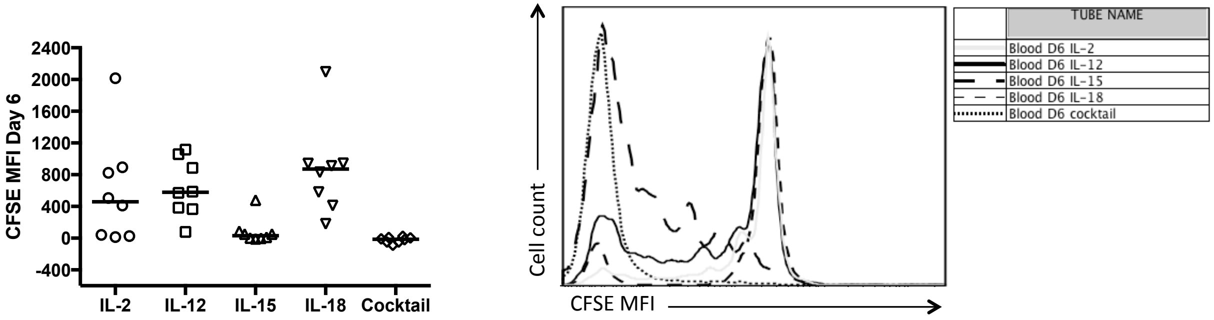

IL-2	IL-12	IL-15	IL-18	Cocktail
459.6	579.1	30.52	870.9	-13.11

Supplementary Figure 2. a) Day 6 CFSE MFI of hepatic NK cells following culture with IL-2, IL-12, IL-15, IL-18 and the cytokine cocktail. Median values displayed below**.** Dot plots display individual values and median. Representative flow cytometry histograms from one individual show CFSE expression at day 6 following culture with IL-2, IL-12, IL-15, IL-18 and the cytokine cocktail. **b)** Day 6 CFSE MFI of peripheral blood NK cells following culture with IL-2, IL-12, IL-15, IL-18 and the cytokine cocktail. Median values displayed below**.** Median values displayed below**.** Dot plots display individual values and median. Representative flow cytometry histograms from one individual show CFSE MFI at day 6 following culture with IL-2, IL-12, IL-15, IL-18 and the cytokine cocktail.

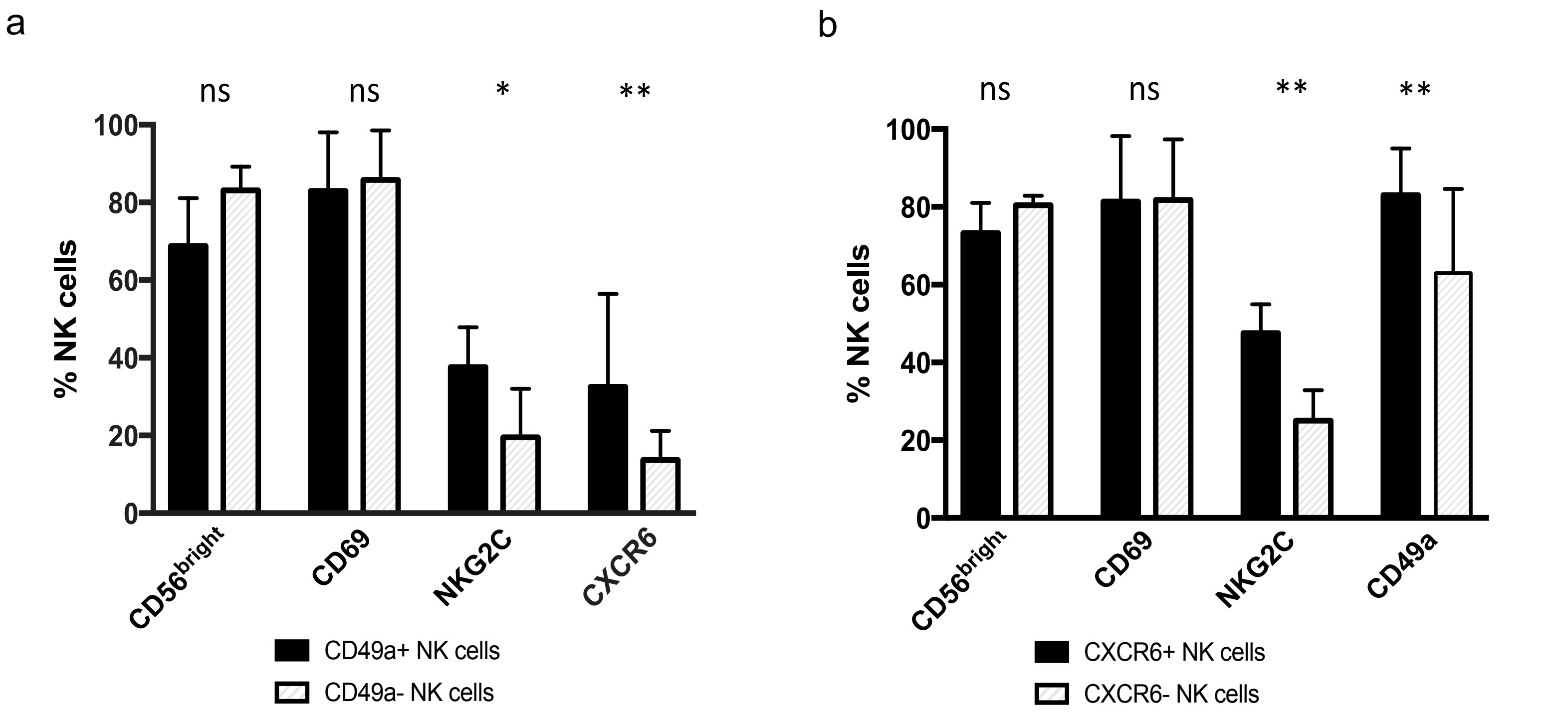

Supplementary Figure 3. a) A comparison of CD56^{bright}, CD69+, NKG2C+ and CXCR6+ NK cell frequencies found within CD49a+ and CD49a- NK subsets generated in the peripheral blood following 6 days of culture with IL-15 (n=9). Bar chart displays median and interquartile range. (Wilcoxon matched pairs test). **b)** A comparison of CD56^{bright}, CD69+, NKG2C+ and CD49a+ NK cell frequencies found within CXCR6+ and CXCR6- NK subsets generated in the peripheral blood following 6 days of culture with IL-15 (n=9). Bar chart displays median and interquartile range. (Wilcoxon matched pairs test). median. $p<0.05$*, $p<0.01$**.

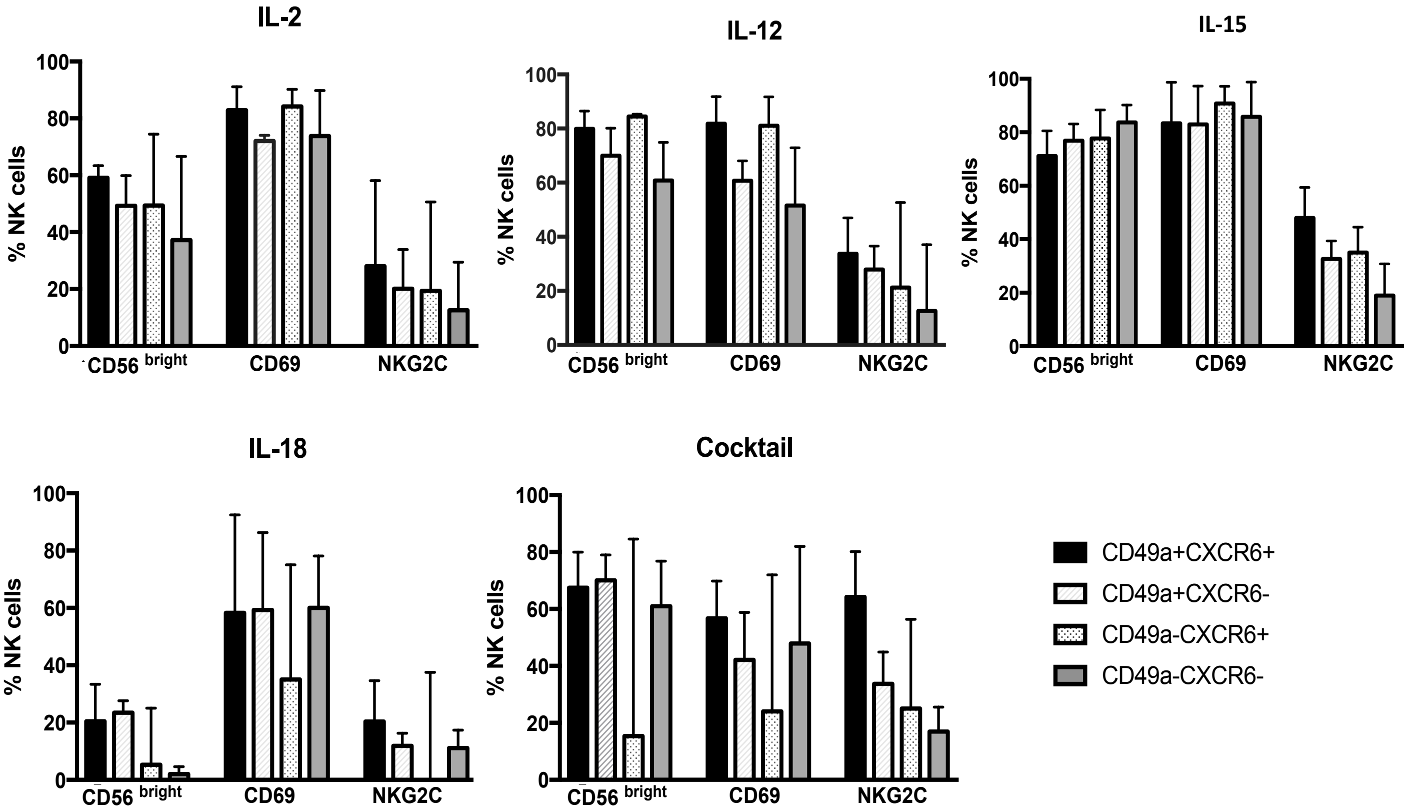

Supplementary Figure 4. A comparison of CD56^{bright}, CD69+ and NKG2C+ NK cell frequencies between CD49a+CXCR6+, CD49a+CXCR6-, CD49a-CXCR6+, CD49a-CXCR6- NK subsets generated in the peripheral blood following 6 days of culture with IL-2, IL-12, IL-15, IL-18 and a cocktail of all four cytokines (n=9). Bar chart displays median and interquartile range.
