# Supplementary material for: IL‐12 and IL‐15 induce the expression of CXCR6 and CD49a on peripheral natural killer cells
Source: Immun Inflamm Dis. 2017 Sep 27;6(1):34–46. doi: 10.1002/iid3.190 (PMC5818449; doi:10.1002/iid3.190)
Supplement: Supplementary file 2 — Table S2. Differential expression of genes between CXCR6+ and CXCR6‐ NK cells. [file IID3-6-34-s002.pdf]

Supplementary table 2: Differential expression of genes between CXCR6+ and CXCR6- NK cells

	baseMean	log2FoldChange	lfcSE	stat	pvalue	padj
CX3CR1	1156.401623	-7.980994386	0.52884912	-15.09125	1.85E-51	3.65E-47
PPM1L	583.6948367	-4.9054212	0.330436319	-14.845285	7.46E-50	7.37E-46
MTSS1	619.5951011	-4.72030262	0.343356137	-13.747541	5.27E-43	3.47E-39
PXN	712.4393154	-5.428799533	0.414634737	-13.092968	3.61E-39	1.78E-35
LGR6	223.6987026	-6.498281421	0.517779783	-12.55028	3.96E-36	1.57E-32
S1PR1	314.2016863	-7.031692305	0.562416541	-12.502641	7.22E-36	2.38E-32
CMKLR1	269.2159895	-6.560409963	0.53635757	-12.231411	2.11E-34	5.96E-31
GZMB	2299.433909	-4.995093291	0.412336243	-12.114126	8.89E-34	2.20E-30
PDE3B	395.7542464	-5.658980336	0.471765893	-11.995315	3.76E-33	8.25E-30
GNLY	8886.467113	-5.650735237	0.477491549	-11.83421	2.60E-32	5.13E-29
ATP10A	366.3147195	-4.172082588	0.355950445	-11.720965	9.95E-32	1.79E-28
KIR3DX1	232.9493831	-7.200133678	0.618516981	-11.640964	2.55E-31	4.20E-28
SYNE1	10726.48669	-3.042275079	0.267313602	-11.380921	5.20E-30	7.91E-27
AGAP1	204.0350272	-6.961487363	0.618091323	-11.262878	2.00E-29	2.82E-26
ERBB2	447.4176719	-3.966808571	0.355937564	-11.144675	7.60E-29	1.00E-25
NHSL2	242.1828161	-6.041681293	0.552826104	-10.928719	8.40E-28	1.04E-24
AHNAK	11834.85436	-4.050701497	0.370917755	-10.920754	9.17E-28	1.07E-24
B3GAT1	345.6939897	-8.929690252	0.818217463	-10.91359	9.93E-28	1.09E-24
FCGR3A	1814.013634	-3.239740659	0.302917397	-10.695129	1.07E-26	1.12E-23
GZMH	642.4817306	-3.556633866	0.345023521	-10.308381	6.46E-25	6.38E-22
OSBPL5	853.8477407	-3.206266313	0.312178922	-10.270605	9.56E-25	8.99E-22
SYNJ2	165.4770638	-4.703168143	0.459918597	-10.226088	1.52E-24	1.36E-21
ADAMTS17	1251.38346	3.081798753	0.306256673	10.0627971	8.07E-24	6.93E-21
SYT11	292.4670117	-3.327566842	0.331388296	-10.041293	1.00E-23	8.26E-21
PTPRD	222.0815116	3.61398661	0.360217906	10.0327789	1.09E-23	8.39E-21
GPR141	177.2306492	-7.948604016	0.7923382	-10.031832	1.10E-23	8.39E-21
FAT4	175.0665409	-3.987543324	0.400331591	-9.9606012	2.27E-23	1.66E-20
SYNE2	9492.098662	-2.991880991	0.305181144	-9.8036233	1.09E-22	7.66E-20
P2RY8	637.411718	-3.42167291	0.353081197	-9.6908953	3.30E-22	2.25E-19
SERPINB9	327.4432227	-5.014313152	0.522170858	-9.6028207	7.78E-22	5.12E-19
ATXN7L1	92.08462477	-5.369354822	0.561719556	-9.5587821	1.19E-21	7.59E-19
STK38	1125.687869	-2.837611559	0.302417561	-9.3830912	6.41E-21	3.96E-18
DTHD1	651.9953874	-4.385634537	0.46878052	-9.3554112	8.33E-21	4.99E-18
TOX2	327.6850266	3.772486461	0.405134837	9.31168123	1.26E-20	7.31E-18
SLCO4C1	254.7138035	-3.495940576	0.383936063	-9.105528	8.58E-20	4.85E-17
TTC16	105.7462665	-5.287219155	0.594609744	-8.8919147	6.01E-19	3.30E-16
FAM65B	1643.237615	-2.669913391	0.301552207	-8.853901	8.45E-19	4.39E-16
CXCR2	128.2030049	-5.543984957	0.626059884	-8.8553589	8.34E-19	4.39E-16
CD52	121.4370905	-4.757064861	0.539277309	-8.8211849	1.13E-18	5.74E-16
SPTBN5	88.31704364	-4.88408546	0.554285458	-8.8114985	1.23E-18	6.10E-16
UBASH3A	124.3906053	3.597101877	0.409132383	8.79202435	1.47E-18	7.08E-16
XCL1	648.8994594	3.161995521	0.36271465	8.71758426	2.84E-18	1.34E-15
LIF	442.9609489	3.044709371	0.350322484	8.69116175	3.59E-18	1.65E-15
ARVCF	161.5725168	-3.418274792	0.393495722	-8.6869427	3.72E-18	1.67E-15
GK5	1411.683712	-2.518261852	0.291044572	-8.6524955	5.04E-18	2.21E-15

SELL	357.47285	-4.711743363	0.547520308	-8.6056048	7.59E-18	3.23E-15
RASA3	1164.700542	-3.67499634	0.42712409	-8.6040484	7.70E-18	3.23E-15
SBK1	112.283576	-4.584520356	0.533582259	-8.5919655	8.55E-18	3.52E-15
KRT72	113.0390357	-6.050882196	0.705546871	-8.5761591	9.81E-18	3.95E-15
CAPN2	794.2785472	-4.24648981	0.495725266	-8.5662162	1.07E-17	4.23E-15
CD28	138.1372008	3.954014291	0.464377896	8.51464793	1.67E-17	6.47E-15
FRY	190.4141246	-3.550640856	0.418215608	-8.4899769	2.07E-17	7.85E-15
KIR2DL1	95.46831745	-5.468412996	0.645514924	-8.4713967	2.42E-17	9.04E-15
LRRC8C	298.3626647	-3.363882391	0.397606335	-8.460334	2.67E-17	9.75E-15
RGS9	101.2615334	-3.683064969	0.435567897	-8.4557769	2.77E-17	9.96E-15
S100A4	470.436332	-3.31031848	0.391993401	-8.4448322	3.04E-17	1.07E-14
TNFSF14	655.0237568	2.296638199	0.273025993	8.41179324	4.04E-17	1.40E-14
SPRY1	462.1882366	2.848233774	0.341710765	8.33521816	7.74E-17	2.63E-14
SELPLG	928.7254835	-3.862333691	0.464281207	-8.3189533	8.87E-17	2.97E-14
VCL	684.6127911	-4.228368059	0.512963955	-8.243012	1.68E-16	5.53E-14
SPON2	2490.869932	-2.944230026	0.358124699	-8.2212426	2.01E-16	6.52E-14
LAIR1	241.4006477	-3.526658163	0.430533801	-8.1913619	2.58E-16	8.23E-14
SSBP3	129.2021884	-3.409264724	0.419259298	-8.1316377	4.24E-16	1.33E-13
USP6NL	151.2400056	2.949136365	0.365336131	8.07239173	6.89E-16	2.13E-13
CACNA2D2	135.6819698	-3.433228289	0.433714928	-7.9158638	2.46E-15	7.46E-13
PLXNA4	139.5754648	-4.142954799	0.525757062	-7.8799794	3.27E-15	9.80E-13
CXCR6	1272.207071	3.075824148	0.390977401	7.86701261	3.63E-15	1.07E-12
GNAL	117.0628894	-3.451750762	0.444643924	-7.762955	8.30E-15	2.41E-12
NME8	108.0401933	-9.017606704	1.166019657	-7.7336661	1.04E-14	2.99E-12
MAF	388.5160366	-2.770939132	0.359717964	-7.703088	1.33E-14	3.75E-12
ADGRG3	574.7305747	2.803347262	0.364596735	7.68889844	1.48E-14	4.13E-12
ENC1	728.4432858	-2.882132099	0.376360292	-7.657907	1.89E-14	5.19E-12
KRT73	65.6178742	-5.67083512	0.740741295	-7.6556217	1.92E-14	5.21E-12
TIE1	241.4683342	2.766636374	0.361571339	7.65170265	1.98E-14	5.30E-12
SLC1A7	84.05412558	-7.85941872	1.029268059	-7.6359299	2.24E-14	5.91E-12
CARMIL3	60.80442359	-4.695704048	0.616582446	-7.6156953	2.62E-14	6.82E-12
AKR1C3	81.67474117	-9.537318967	1.260130512	-7.5685168	3.78E-14	9.69E-12
STRBP	341.222707	2.507185251	0.332002019	7.55171688	4.30E-14	1.09E-11
ADGRG1	2082.225216	-2.890494969	0.383866063	-7.529957	5.08E-14	1.27E-11
MIAT	355.2776987	-3.386234232	0.450845577	-7.5108516	5.87E-14	1.45E-11
NTNG2	167.3435304	-4.107992635	0.54769335	-7.5005341	6.36E-14	1.55E-11
BTBD11	94.70638788	-5.643532367	0.75327253	-7.4920193	6.78E-14	1.61E-11
PCSK5	91.86462617	-4.335409284	0.578572627	-7.4932845	6.72E-14	1.61E-11
ASCL2	57.14082303	-6.291905193	0.842220526	-7.4706149	7.98E-14	1.88E-11
XCL2	412.1816523	2.541884275	0.340346068	7.4685284	8.11E-14	1.88E-11
RAP1GAP2	887.5840343	-6.520214652	0.873244715	-7.4666523	8.23E-14	1.89E-11
CDC25B	348.0045848	-2.773112347	0.372629266	-7.4420144	9.92E-14	2.25E-11
FGFBP2	1248.932267	-7.374878465	0.996253487	-7.4026124	1.34E-13	2.98E-11
CEP78	2070.437589	-2.048724434	0.276784231	-7.4018828	1.34E-13	2.98E-11
DAB2	143.6874237	-4.094604433	0.553846558	-7.3930304	1.44E-13	3.15E-11
EMP3	203.9008485	-3.007520122	0.407158005	-7.3866167	1.51E-13	3.27E-11
SOX13	59.4063161	-6.388014959	0.865430758	-7.3813126	1.57E-13	3.37E-11
MGAT3	85.50568662	3.8425097	0.521398704	7.36961882	1.71E-13	3.64E-11

GDPD5	86.64749275	-4.822926699	0.660423825	-7.3027752	2.82E-13	5.92E-11
RP11-20I20.4	130.360126	-3.067726669	0.420575471	-7.2941169	3.01E-13	6.25E-11
TFEB	248.715603	-3.031958361	0.419489392	-7.2277355	4.91E-13	1.01E-10
DOK6	219.2177995	-2.948623743	0.410303138	-7.1864518	6.65E-13	1.35E-10
MGAM	199.4812478	-4.128175317	0.579425993	-7.1245946	1.04E-12	2.10E-10
RASSF4	424.3853219	-2.448071743	0.344010121	-7.1162783	1.11E-12	2.21E-10
PTCH1	267.656352	-2.662555046	0.374564335	-7.1084051	1.17E-12	2.32E-10
LINC00299	521.267032	-2.360514606	0.334527443	-7.056266	1.71E-12	3.35E-10
DAB2IP	288.8293581	3.060722491	0.434008732	7.05221407	1.76E-12	3.41E-10
CCDC141	594.8751439	2.326124588	0.330482768	7.03856543	1.94E-12	3.73E-10
DRAXIN	97.80459698	-3.952430989	0.564149572	-7.0059984	2.45E-12	4.66E-10
CLNK	190.0303066	3.196331054	0.457255921	6.9902453	2.74E-12	5.16E-10
SGSM1	58.12165111	-7.376881842	1.056627475	-6.9815351	2.92E-12	5.44E-10
CAST	1024.540607	-1.905057835	0.272972174	-6.9789452	2.97E-12	5.44E-10
TMEM173	156.6500201	-3.068316564	0.439651383	-6.9789763	2.97E-12	5.44E-10
ESR2	67.58276179	-3.59153683	0.516636412	-6.9517687	3.61E-12	6.54E-10
PAWR	110.5030152	2.550412399	0.368288411	6.92504115	4.36E-12	7.83E-10
LINC00298	58.56315198	-3.775028958	0.545794434	-6.9165765	4.63E-12	8.24E-10
ITGAX	945.0229295	-2.807436834	0.406011286	-6.9146768	4.69E-12	8.27E-10
AKT3	674.4564308	-2.128318435	0.30861026	-6.8964604	5.33E-12	9.32E-10
KIR2DP1	72.25052495	-3.781712294	0.548499315	-6.8946527	5.40E-12	9.36E-10
SULT1B1	87.41070551	-4.567916383	0.664217516	-6.8771393	6.11E-12	1.05E-09
KIAA1644	41.61193019	6.988359679	1.016946175	6.87190714	6.33E-12	1.08E-09
GRAP2	217.1640349	-2.402720509	0.349822154	-6.8684058	6.49E-12	1.10E-09
FAM169A	242.5765203	-2.432157023	0.35451948	-6.8604327	6.87E-12	1.15E-09
CERCAM	47.65040929	-4.845299781	0.707042667	-6.85291	7.24E-12	1.19E-09
EPB41L4A	68.32544227	-3.746014436	0.546573168	-6.8536376	7.20E-12	1.19E-09
SH3BP5	112.5262308	-2.748424493	0.401417216	-6.8468027	7.55E-12	1.22E-09
KIR3DL1	102.9983775	-4.258940596	0.622049274	-6.846629	7.56E-12	1.22E-09
CDHR1	756.4162987	2.710967214	0.396540571	6.83654439	8.11E-12	1.30E-09
IL7R	166.5106465	-2.901313282	0.425214822	-6.8231706	8.91E-12	1.42E-09
CASK	382.7730717	2.068680053	0.304105059	6.80251772	1.03E-11	1.62E-09
FGL2	521.482293	-3.500941388	0.517494152	-6.7651806	1.33E-11	2.09E-09
GLB1L2	42.10631521	-5.115438305	0.756555063	-6.7614884	1.37E-11	2.12E-09
PELI2	326.6434518	-2.327144173	0.346060302	-6.7246782	1.76E-11	2.72E-09
CDC42EP3	322.7528628	-2.458401291	0.367415147	-6.6910722	2.22E-11	3.39E-09
CHN2	587.8037156	1.907866016	0.285580596	6.68065702	2.38E-11	3.59E-09
TLR3	38.97296136	-5.358326693	0.80202662	-6.6809836	2.37E-11	3.59E-09
MC1R	157.8537218	-2.954997231	0.443154566	-6.6680961	2.59E-11	3.88E-09
LAIR2	54.33467295	-7.980992638	1.198290627	-6.6603147	2.73E-11	4.06E-09
UAP1	383.9307801	-2.167726907	0.326390651	-6.6415104	3.10E-11	4.58E-09
SPRY2	879.3315992	2.460579289	0.374485667	6.57055665	5.01E-11	7.34E-09
CTD-3076O17.2	36.92321016	4.361677718	0.665117013	6.55775996	5.46E-11	7.93E-09
CCR5	238.7523578	2.787938815	0.426063927	6.54347538	6.01E-11	8.67E-09
RARG	56.20378358	-3.815133377	0.584526655	-6.5268767	6.72E-11	9.61E-09
LDB2	317.5455652	2.259897686	0.347984148	6.49425469	8.34E-11	1.19E-08
RASGRP2	773.7993103	-2.261435869	0.348522568	-6.4886354	8.66E-11	1.22E-08
ABCG1	183.1367324	2.659569081	0.412068241	6.45419572	1.09E-10	1.51E-08

CDHR3	37.71736579	-5.71108133	0.884842767	-6.454346	1.09E-10	1.51E-08
IL2RB	9267.029127	1.816137142	0.28144692	6.45285848	1.10E-10	1.52E-08
RP11-90E5.1	60.22051882	3.071810416	0.47733002	6.43540168	1.23E-10	1.69E-08
CHD7	204.2673563	-2.371045043	0.368723462	-6.4304154	1.27E-10	1.73E-08
LINC00565	37.24742189	-8.419881509	1.311171559	-6.4216475	1.35E-10	1.82E-08
LINC01146	62.6445092	-3.589987221	0.559202303	-6.4198363	1.36E-10	1.83E-08
C1orf21	766.8809444	-1.823096435	0.28490462	-6.3989711	1.56E-10	2.09E-08
TIGIT	1828.843705	1.742666401	0.273790729	6.36495766	1.95E-10	2.59E-08
HS6ST1	51.82648529	-3.86356364	0.610085524	-6.332823	2.41E-10	3.17E-08
AFAP1	41.89255963	-4.989051751	0.789987334	-6.3153566	2.70E-10	3.53E-08
LYAR	154.6089467	-2.466320504	0.390784838	-6.311198	2.77E-10	3.60E-08
RP11-93B14.9	161.5441624	2.11129844	0.334654239	6.30889496	2.81E-10	3.63E-08
PRKCA	507.3950073	1.785750092	0.283564732	6.2975042	3.02E-10	3.88E-08
PATJ	510.1534777	2.147236858	0.341289942	6.29153279	3.14E-10	4.01E-08
DZIP3	742.7449615	2.086837322	0.331977153	6.28608716	3.26E-10	4.12E-08
NEO1	546.5114324	2.376335682	0.381053951	6.23621846	4.48E-10	5.64E-08
CALHM2	103.0091044	-2.408658574	0.38740468	-6.2174225	5.05E-10	6.32E-08
GZMK	301.4876087	2.658151674	0.427610169	6.21629668	5.09E-10	6.32E-08
DENND5A	436.6213859	2.124661277	0.342480636	6.20374134	5.51E-10	6.81E-08
GRAMD3	285.7306525	2.210979105	0.356897183	6.19500297	5.83E-10	7.15E-08
ARL4C	2879.026686	-2.376196203	0.383781113	-6.1915402	5.96E-10	7.27E-08
FGFR1	96.20959158	-4.313067539	0.698101147	-6.1782846	6.48E-10	7.85E-08
FGD4	144.7834221	2.23158189	0.361581	6.17173439	6.75E-10	8.14E-08
FAM129A	541.055833	-2.910192828	0.471710194	-6.1694508	6.85E-10	8.21E-08
ITGB7	299.9970558	-3.223154336	0.523745959	-6.1540414	7.55E-10	8.99E-08
TGFA	359.1117435	2.741240703	0.445610005	6.15165878	7.67E-10	9.07E-08
ANK3	145.6542351	2.913841995	0.474225931	6.14441727	8.03E-10	9.44E-08
DNMT3A	602.4217135	1.806838896	0.294162744	6.14231045	8.13E-10	9.51E-08
BANK1	105.7216557	2.955316655	0.481338882	6.13978377	8.26E-10	9.60E-08
RP11-229P13.19	29.31382496	-8.07045423	1.315620433	-6.1343333	8.55E-10	9.88E-08
GREM2	52.44479444	3.167401218	0.516847461	6.12830953	8.88E-10	1.02E-07
DAPK2	332.6832632	2.499321272	0.409061515	6.109891	9.97E-10	1.14E-07
RAP2A	378.1209704	-2.077876519	0.340151733	-6.1086754	1.00E-09	1.14E-07
DOCK7	111.9731097	2.190193889	0.359702086	6.08891073	1.14E-09	1.28E-07
GAS7	450.2668625	-2.134821482	0.351794453	-6.0683773	1.29E-09	1.45E-07
RP11-532M24.1	31.48156462	-5.945414729	0.98229388	-6.0525825	1.43E-09	1.58E-07
PLEKHA1	687.0374397	-1.846111984	0.304995023	-6.0529249	1.42E-09	1.58E-07
TRPM2	360.8450119	2.021168613	0.334451891	6.04322674	1.51E-09	1.67E-07
SSX2IP	177.3505015	-2.344984621	0.392168534	-5.9795328	2.24E-09	2.46E-07
GOLM1	114.0485134	-2.358062739	0.394487574	-5.9775336	2.27E-09	2.47E-07
EEF1DP3	24.73508298	-7.80728745	1.313407103	-5.9443012	2.78E-09	3.01E-07
LEXM	32.58139416	-6.515659812	1.096668918	-5.9413189	2.83E-09	3.05E-07
HDAC9	759.8569285	2.378457376	0.400873301	5.93318978	2.97E-09	3.19E-07
CNR2	194.1633307	2.632617224	0.444147931	5.92734322	3.08E-09	3.29E-07
DSTN	185.3641599	-1.996902403	0.336969049	-5.9260707	3.10E-09	3.30E-07
ME1	59.74507027	3.89896077	0.65908635	5.91570554	3.30E-09	3.49E-07
PRSS35	24.30406282	5.730341167	0.969057155	5.91331599	3.35E-09	3.52E-07
CD226	1257.524541	-2.429735621	0.411113945	-5.910127	3.42E-09	3.57E-07

CD27	27.94960806	4.168973426	0.706568845	5.90030746	3.63E-09	3.77E-07
FCGR2C	81.48102224	-3.038274903	0.515892503	-5.8893566	3.88E-09	4.01E-07
HAVCR1	54.12402357	-2.999630074	0.509996068	-5.8816729	4.06E-09	4.18E-07
CCND1	67.65712172	2.733854614	0.466037791	5.86616508	4.46E-09	4.57E-07
TSPAN2	55.0027525	-3.909783401	0.669296452	-5.8416317	5.17E-09	5.26E-07
SPECC1	752.008577	1.718544432	0.294780532	5.82991157	5.55E-09	5.62E-07
TSPAN14	739.5390697	-1.665414052	0.287172552	-5.7993497	6.66E-09	6.71E-07
SWAP70	269.2774713	1.909756669	0.329456356	5.79669093	6.76E-09	6.75E-07
KLF3	772.6481306	-2.140282965	0.369232128	-5.7965784	6.77E-09	6.75E-07
SPTB	42.57539795	-4.643089507	0.801595532	-5.7923096	6.94E-09	6.89E-07
AKR1C7P	22.53892477	-7.685354506	1.328907199	-5.7832138	7.33E-09	7.24E-07
SIGLEC9	45.01581388	-4.392284665	0.760707635	-5.7739458	7.74E-09	7.61E-07
ADAMTS1	393.4635437	-2.876506001	0.499649725	-5.7570451	8.56E-09	8.37E-07
RP11-677N16.1	30.67063071	-4.657946607	0.810319734	-5.7482823	9.02E-09	8.77E-07
SERPINE2	152.9191099	2.476049293	0.432067781	5.73069644	1.00E-08	9.69E-07
SLC4A4	823.6310965	1.565454342	0.27335909	5.72673235	1.02E-08	9.87E-07
CRTAM	280.9953101	2.085101337	0.364826959	5.71531596	1.10E-08	1.05E-06
PRF1	13439.26399	-1.826871357	0.319993127	-5.7090956	1.14E-08	1.08E-06
MAML3	105.9719721	2.228077167	0.390416981	5.70691665	1.15E-08	1.09E-06
PTPRB	34.24431389	-5.02404783	0.880737527	-5.7043644	1.17E-08	1.10E-06
BAALC	138.0863142	2.377999193	0.417311788	5.69837532	1.21E-08	1.14E-06
LINC01451	29.10098492	-7.116815176	1.250099015	-5.6930012	1.25E-08	1.17E-06
HS3ST3B1	261.5682627	-2.126213759	0.373639573	-5.6905476	1.27E-08	1.18E-06
KIF13A	109.2302908	-3.507323098	0.618442113	-5.6712229	1.42E-08	1.32E-06
PRNP	274.2441563	-2.285881802	0.403136977	-5.670236	1.43E-08	1.32E-06
BPGM	304.610208	-2.222197151	0.395075618	-5.6247388	1.86E-08	1.71E-06
CD160	1745.175112	2.041086939	0.363090269	5.62143113	1.89E-08	1.73E-06
C19orf84	54.8977635	-2.888977819	0.514062904	-5.6198916	1.91E-08	1.74E-06
ANXA1	1929.302846	-1.994406918	0.35583048	-5.6049356	2.08E-08	1.89E-06
CSF2	54.12349309	2.847859706	0.508920159	5.59588701	2.19E-08	1.98E-06
S100B	35.00564039	-5.081477843	0.910813156	-5.5790563	2.42E-08	2.17E-06
RP4-738P11.4	79.39206824	2.753105882	0.493579088	5.57784142	2.44E-08	2.18E-06
SH2D1A	1290.657661	1.551326258	0.278227666	5.57574407	2.46E-08	2.19E-06
CD55	522.4517637	-2.157736575	0.387094094	-5.5741914	2.49E-08	2.20E-06
PODN	19.11148828	-7.449316276	1.339256753	-5.5622764	2.66E-08	2.35E-06
COL13A1	27.6821988	-6.201008923	1.115235198	-5.5602701	2.69E-08	2.37E-06
MAPK12	84.07994316	2.614046398	0.470577461	5.55497578	2.78E-08	2.43E-06
EBF4	30.28460876	-4.282468263	0.772889209	-5.5408566	3.01E-08	2.62E-06
TBC1D4	90.16487134	2.326312761	0.422610394	5.50462742	3.70E-08	3.21E-06
KIF19	24.39975085	-5.253225465	0.955087085	-5.5002581	3.79E-08	3.27E-06
FZD4	25.52794176	-5.025062782	0.913937769	-5.4982549	3.84E-08	3.29E-06
FLNA	12483.05232	-2.529232017	0.460783199	-5.4889849	4.04E-08	3.46E-06
MYRF	61.04989576	-2.856263704	0.521011086	-5.4821553	4.20E-08	3.56E-06
ATP9A	193.689666	-2.410232907	0.439603438	-5.4827435	4.19E-08	3.56E-06
ZEB1	348.6009545	1.651613733	0.302179436	5.46567216	4.61E-08	3.89E-06
RP11-345J18.2	47.78000586	-3.272034501	0.599148125	-5.4611445	4.73E-08	3.98E-06
NSG1	34.3223889	-3.439037338	0.631562744	-5.4452821	5.17E-08	4.33E-06
ANXA4	91.45564284	-2.295180845	0.421974236	-5.4391492	5.35E-08	4.46E-06

ACTN1	154.8552327	2.260761378	0.416097293	5.43325183	5.53E-08	4.59E-06
GSTO2	85.55961643	-2.887713877	0.531534366	-5.4327887	5.55E-08	4.59E-06
RAP2B	522.9811921	-1.658015147	0.305441568	-5.4282564	5.69E-08	4.68E-06
MBOAT1	45.59772752	2.892434127	0.53298952	5.42681238	5.74E-08	4.70E-06
LZTFL1	630.9189426	1.855473933	0.342442689	5.4183488	6.02E-08	4.91E-06
EVA1C	152.118722	-2.14035831	0.395313592	-5.4143302	6.15E-08	5.00E-06
PLCH2	1313.022124	1.787493487	0.330877447	5.40228264	6.58E-08	5.33E-06
DHCR24	64.59010831	-3.295918175	0.612804328	-5.3784186	7.51E-08	6.06E-06
SSBP3-AS1	39.66806137	-3.460393896	0.64377005	-5.3752017	7.65E-08	6.14E-06
HIP1	444.9783493	1.570838389	0.292444852	5.37140038	7.81E-08	6.25E-06
MMRN1	53.25307384	2.44186203	0.455654604	5.35901977	8.37E-08	6.67E-06
NAAA	85.49686777	-2.381648903	0.445271626	-5.3487551	8.86E-08	7.03E-06
SPIRE2	76.03573078	-2.907754551	0.543716947	-5.3479197	8.90E-08	7.03E-06
IL12RB1	339.3831198	-1.839652189	0.344160933	-5.3453254	9.03E-08	7.10E-06
SMPD3	175.7660643	2.220436127	0.415584417	5.34292441	9.15E-08	7.17E-06
ZNF667	31.53357474	3.44690458	0.646555908	5.33117792	9.76E-08	7.62E-06
SORL1	6778.43211	-1.582838855	0.296982203	-5.3297431	9.84E-08	7.65E-06
WDR49	72.81585568	2.174640703	0.408109994	5.32856518	9.90E-08	7.66E-06
CEBPB	266.791111	-2.816817987	0.528745945	-5.3273562	9.97E-08	7.66E-06
RP3-467K16.4	168.1591838	-1.95253358	0.366507879	-5.3273987	9.96E-08	7.66E-06
ITGA5	535.8096002	-5.414545833	1.016970276	-5.3241928	1.01E-07	7.76E-06
LTBP3	543.1720559	1.635634552	0.307923066	5.31182861	1.09E-07	8.28E-06
DGKK	137.5595736	-7.518565232	1.419164344	-5.297882	1.17E-07	8.90E-06
PCDHGB6	36.76801209	-3.145236958	0.594945052	-5.2866008	1.25E-07	9.43E-06
CHSY3	34.41399714	-4.436703826	0.845052638	-5.25021	1.52E-07	1.15E-05
AC018634.9	15.97972573	-7.169902879	1.365882185	-5.2492835	1.53E-07	1.15E-05
FMN1	29.41931865	-6.369930666	1.214507924	-5.2448655	1.56E-07	1.17E-05
TNFRSF11A	24.71403388	-4.433628453	0.846148086	-5.2397784	1.61E-07	1.19E-05
RP11-627G23.1	20.62682797	-6.645116887	1.268151319	-5.2400031	1.61E-07	1.19E-05
PRR5	208.2340526	-1.984358102	0.378729013	-5.2395196	1.61E-07	1.19E-05
JAKMIP2	142.418393	-2.729149898	0.520946858	-5.2388259	1.62E-07	1.19E-05
SMPDL3B	50.37460547	2.515956163	0.48172108	5.22284838	1.76E-07	1.29E-05
GRAMD1A	373.054433	1.713191563	0.328227271	5.21952839	1.79E-07	1.31E-05
LIPC	28.0394079	-3.662188993	0.702836695	-5.2105831	1.88E-07	1.37E-05
ZEB2-AS1	179.7735152	-1.965069693	0.3773957	-5.2069213	1.92E-07	1.39E-05
TTN	7549.353461	1.730087162	0.332784122	5.19882725	2.01E-07	1.45E-05
PTGDS	257.2378447	-7.279168561	1.408577386	-5.1677449	2.37E-07	1.71E-05
INPP4B	95.9794582	2.410786476	0.467037665	5.16186736	2.44E-07	1.76E-05
IQSEC1	653.6342079	-1.984401113	0.386014414	-5.1407436	2.74E-07	1.96E-05
LCNL1	17.62384644	-7.333580711	1.430973352	-5.1248898	2.98E-07	2.12E-05
LIM2	26.79847728	-3.528027333	0.688561167	-5.1237675	2.99E-07	2.13E-05
TBX21	1297.091247	-1.576111836	0.308082454	-5.1158767	3.12E-07	2.21E-05
CABLES1	139.4534978	2.459081737	0.480965984	5.11279762	3.17E-07	2.24E-05
JAG2	105.0211904	2.278265256	0.445687457	5.11180025	3.19E-07	2.24E-05
DAAM1	123.226799	-1.928592109	0.377454573	-5.1094681	3.23E-07	2.26E-05
GNAQ	318.2836176	2.113217931	0.41403958	5.10390318	3.33E-07	2.32E-05
ADAM22	59.00702844	-2.726204411	0.534241763	-5.1029414	3.34E-07	2.33E-05
TMCC3	286.1676588	-2.031490704	0.398958555	-5.0919843	3.54E-07	2.46E-05

AGPAT4	532.7536052	-2.153361248	0.423315816	-5.0868906	3.64E-07	2.51E-05
SLAIN1	98.09609995	-2.103063254	0.413785897	-5.0824914	3.73E-07	2.56E-05
LY9	528.8852126	1.562914911	0.30856877	5.06504566	4.08E-07	2.80E-05
GYG1	464.8537673	1.388002386	0.274665633	5.05342577	4.34E-07	2.97E-05
PCDHGA10	29.77524561	-3.382770331	0.670106184	-5.0481109	4.46E-07	3.03E-05
ZEB2	4071.091806	-1.731256198	0.342961593	-5.0479594	4.47E-07	3.03E-05
LRFN2	14.62498357	-7.081295516	1.403435769	-5.0456855	4.52E-07	3.06E-05
THBS1	992.9535466	1.76625538	0.35047493	5.03960548	4.66E-07	3.15E-05
HIVEP1	577.970635	1.38724863	0.275363254	5.03788582	4.71E-07	3.16E-05
GCSAM	104.9614085	2.503252475	0.497158756	5.03511694	4.78E-07	3.20E-05
PARP15	828.8435335	-1.634332755	0.324855475	-5.0309534	4.88E-07	3.26E-05
NINL	35.36583122	-3.427807505	0.68229066	-5.0239696	5.06E-07	3.37E-05
LGALS1	169.5568676	-2.170922993	0.433954861	-5.002647	5.65E-07	3.75E-05
FZD3	25.41024424	-3.546320788	0.708994022	-5.0019051	5.68E-07	3.75E-05
LTBP4	139.5767772	-1.981627135	0.396519533	-4.9975524	5.81E-07	3.81E-05
ABCA1	162.0150123	2.910766128	0.582381603	4.99803928	5.79E-07	3.81E-05
CLIP2	242.3889612	1.781992049	0.357086154	4.99037005	6.03E-07	3.94E-05
C20orf166-AS1	28.57282707	4.059889488	0.814933415	4.98186651	6.30E-07	4.11E-05
HPGD	50.76402245	-3.791262868	0.761624993	-4.9778604	6.43E-07	4.18E-05
CTD-3088G3.8	106.152496	-2.372990778	0.478150997	-4.9628481	6.95E-07	4.50E-05
PLOD1	229.920771	-2.4193356	0.489135736	-4.9461436	7.57E-07	4.89E-05
GBP1	154.932807	-2.155378104	0.436424413	-4.9387203	7.86E-07	5.06E-05
TSHZ3	18.50877775	-5.291240225	1.071653438	-4.9374546	7.91E-07	5.08E-05
PWWP2AP1	58.90986938	2.716985888	0.550838057	4.93245856	8.12E-07	5.19E-05
MIR646HG	92.7026353	-2.267264009	0.460025021	-4.9285667	8.28E-07	5.28E-05
EFHC2	29.77512873	-4.761798813	0.966393667	-4.9273903	8.33E-07	5.29E-05
RNU2-63P	62.88260836	2.727486144	0.554487357	4.91893297	8.70E-07	5.51E-05
COTL1	337.4104072	1.744088607	0.354600994	4.91845381	8.72E-07	5.51E-05
SORCS2	20.17500288	-4.477251838	0.912263259	-4.9078507	9.21E-07	5.79E-05
PKD2	69.295806	-2.056306337	0.420915702	-4.8853163	1.03E-06	6.48E-05
FLNB	704.3795578	1.909146158	0.391351967	4.87833541	1.07E-06	6.69E-05
SCD5	47.82628059	-3.419766627	0.702232549	-4.8698492	1.12E-06	6.96E-05
S100A6	315.014218	-2.267528186	0.465830962	-4.867706	1.13E-06	7.01E-05
RAB11FIP1	2626.83209	1.24278048	0.255810997	4.85819802	1.18E-06	7.31E-05
ANTXR2	84.35418326	-3.317021345	0.682720252	-4.8585366	1.18E-06	7.31E-05
PVT1	59.14884952	2.272459604	0.467815387	4.8575991	1.19E-06	7.31E-05
C11orf21	604.91396	-1.501566062	0.309194584	-4.8563789	1.20E-06	7.33E-05
WNT10B	24.47696021	-3.422115112	0.70488025	-4.8548886	1.20E-06	7.37E-05
ADAM15	104.3552898	-2.072500225	0.428207794	-4.8399405	1.30E-06	7.92E-05
SEL1L3	735.8823126	1.299444902	0.268592052	4.83798717	1.31E-06	7.97E-05
AGPAT4-IT1	96.55044471	-2.661053529	0.551640197	-4.8238934	1.41E-06	8.53E-05
SIRPG	22.25280079	3.697102467	0.768442779	4.8111617	1.50E-06	9.07E-05
TBXAS1	312.6419964	-1.773372575	0.36874476	-4.8092143	1.52E-06	9.13E-05
AC006548.28	25.73181914	-4.174059686	0.868344413	-4.8069172	1.53E-06	9.20E-05
CTD-2017C7.3	36.66907242	-2.946722346	0.613496959	-4.8031572	1.56E-06	9.35E-05
GUCY1B3	32.690303	-4.337791837	0.903729105	-4.7998806	1.59E-06	9.48E-05
AKR1C4	12.17282806	-6.787076553	1.416601179	-4.791099	1.66E-06	9.87E-05
LILRB1	111.0864843	-6.299689433	1.317464705	-4.7816761	1.74E-06	0.000103

IFNG-AS1	23.30927326	3.841551058	0.80369633	4.77985393	1.75E-06	0.000104
KRT73-AS1	18.0879786	-6.407167471	1.342215447	-4.773576	1.81E-06	0.000107
TANC2	923.2959619	1.335348102	0.279846972	4.77170824	1.83E-06	0.000107
TRAT1	71.74318951	2.25488991	0.472672184	4.77051535	1.84E-06	0.000108
UNC5C	35.12847961	3.420082667	0.717126166	4.76915058	1.85E-06	0.000108
SNX18P13	24.49332029	-6.068662476	1.273625873	-4.7648706	1.89E-06	0.00011
MT2A	70.8706418	-2.458846629	0.516138598	-4.7639271	1.90E-06	0.00011
ZSWIM5	105.4130616	1.915998788	0.402741038	4.75739646	1.96E-06	0.000114
PLEKHO2	780.8724443	1.512474126	0.318288655	4.75189455	2.02E-06	0.000116
SLC4A10	87.754438	2.665140911	0.561036986	4.75038362	2.03E-06	0.000117
ITGAM	1811.114893	-1.417850667	0.298611451	-4.7481457	2.05E-06	0.000118
ADARB1	379.7158159	1.925756967	0.405984098	4.74342955	2.10E-06	0.00012
RP11-861A13.3	40.15023394	-2.682857776	0.566744562	-4.7338042	2.20E-06	0.000126
ATP6V0E2	58.61785132	-2.172733595	0.459646107	-4.7269705	2.28E-06	0.00013
NUMBL	38.81133312	-2.799939145	0.593713064	-4.7159804	2.41E-06	0.000137
BCL2	867.0430339	-1.419457847	0.301471419	-4.7084326	2.50E-06	0.000141
ST6GAL1	731.5383117	-1.545820524	0.329150035	-4.6964009	2.65E-06	0.000149
RGAG4	18.49463503	-5.474786215	1.166157397	-4.6947232	2.67E-06	0.00015
CYSLTR2	309.4648742	1.874254181	0.399297728	4.6938764	2.68E-06	0.00015
DKK3	103.2352224	3.068235914	0.654087568	4.69086414	2.72E-06	0.000152
ARAP3	42.81796342	-2.738250028	0.584523619	-4.6845841	2.81E-06	0.000157
ZNF683	11.5395067	-6.734908842	1.438002411	-4.6835171	2.82E-06	0.000157
RP1-278C19.8	12.36198298	-6.801527875	1.452498341	-4.6826407	2.83E-06	0.000157
RNF217	79.58804856	1.866405447	0.398753438	4.68060027	2.86E-06	0.000158
SERPINB8	30.32302497	-2.767186786	0.591939389	-4.6747806	2.94E-06	0.000162
PLXNC1	617.8803589	1.524765007	0.326487226	4.67021336	3.01E-06	0.000166
IRF8	1934.967013	1.580790113	0.338820283	4.66557107	3.08E-06	0.000169
PDE7A	3000.099148	1.43982451	0.308779569	4.66295266	3.12E-06	0.000171
AC007349.7	42.8932479	2.707230005	0.58065796	4.6623489	3.13E-06	0.000171
C1R	34.54181306	2.501800658	0.536733118	4.6611632	3.14E-06	0.000171
HLA-DPA1	278.6750143	-1.50865007	0.323855368	-4.6584069	3.19E-06	0.000173
ABCB1	1544.714708	1.376580356	0.295925105	4.65178632	3.29E-06	0.000178
COL5A3	24.11864265	-5.289676351	1.137628984	-4.6497377	3.32E-06	0.000179
IL23R	28.34847307	2.824765299	0.608462787	4.64246189	3.44E-06	0.000185
TMBIM1	463.4522124	-1.891679196	0.40767418	-4.6401742	3.48E-06	0.000187
ICAM1	504.6884764	2.078066491	0.44815624	4.63692414	3.54E-06	0.000189
LIMS1	359.5918266	1.4504785	0.313260163	4.63026798	3.65E-06	0.000195
ANXA2	250.6111863	-1.482630124	0.320207041	-4.6302234	3.65E-06	0.000195
RP11-284N8.3	87.61136808	-2.319101669	0.501210301	-4.6270032	3.71E-06	0.000197
FRMPD3	33.316167	-3.727914252	0.806448572	-4.6226311	3.79E-06	0.000201
KIR2DL3	49.57478305	-3.117204874	0.675945736	-4.61162	4.00E-06	0.00021
F2R	775.0938138	-1.787299921	0.387541851	-4.6118888	3.99E-06	0.00021
AMPD3	122.0242284	1.925088463	0.4180591	4.60482373	4.13E-06	0.000217
TFDP2	893.2574251	-1.260875755	0.273870225	-4.6039169	4.15E-06	0.000217
EXPH5	214.1393542	1.992141981	0.43277076	4.60322684	4.16E-06	0.000217
GPR155	84.87603781	-2.362374292	0.514962826	-4.5874657	4.49E-06	0.000233
AMOT	38.16618369	-2.442298125	0.532405251	-4.5872916	4.49E-06	0.000233
NIPAL2	37.60490377	-3.407893291	0.744572044	-4.5769826	4.72E-06	0.000244

ATP8B4	2078.985492	1.478114817	0.322936014	4.57711359	4.71E-06	0.000244
RP11-445P17.8	10.37957682	-6.574932799	1.437166615	-4.5749273	4.76E-06	0.000246
KIAA0930	442.5157219	1.592602037	0.348309409	4.57237731	4.82E-06	0.000248
ACP5	111.2460054	1.839474178	0.402360821	4.57170301	4.84E-06	0.000248
PDLIM1	103.1858554	1.759779634	0.385888169	4.5603358	5.11E-06	0.000261
CD3G	29.68797691	-3.184407626	0.698697264	-4.5576357	5.17E-06	0.000264
MATN2	47.30731222	2.753115056	0.60477443	4.5523007	5.31E-06	0.00027
ITPRIPL1	170.8232956	-1.547673358	0.340120358	-4.5503697	5.36E-06	0.000272
C12orf75	175.1877609	-1.779763186	0.391403286	-4.547134	5.44E-06	0.000275
LRRC6	22.01704676	-4.270609785	0.940623027	-4.5401927	5.62E-06	0.000284
RP11-467I20.6	10.93001296	7.119113051	1.568347101	4.53924584	5.65E-06	0.000285
PDE4D	2035.687565	-2.011894816	0.443691003	-4.5344503	5.78E-06	0.00029
PLEKHG3	1020.064425	-4.282059511	0.945313884	-4.5297753	5.90E-06	0.000296
AKR1C8P	10.35574725	-6.565876095	1.449749065	-4.5289742	5.93E-06	0.000296
MYO6	114.8578122	-2.17991632	0.481501733	-4.5273281	5.97E-06	0.000298
PALLD	107.7297945	-2.002840285	0.442737516	-4.5237646	6.07E-06	0.000302
SIGLEC10	91.58417773	-2.005993154	0.443589415	-4.5221844	6.12E-06	0.000304
ZNF74	242.5179368	1.419301273	0.314340508	4.51517141	6.33E-06	0.000313
LAT2	1165.271778	1.40836269	0.311936497	4.51490192	6.33E-06	0.000313
LINC01505	16.84934965	-4.263020812	0.945216309	-4.5101008	6.48E-06	0.000319
KLF8	23.85552668	-4.147994241	0.920281672	-4.5073094	6.57E-06	0.000323
EOMES	2163.35995	1.401688016	0.3111033	4.50553889	6.62E-06	0.000325
ZNF362	236.6179603	1.427141247	0.317440833	4.49577087	6.93E-06	0.000339
HDDC2	158.4537129	-2.064734132	0.459465659	-4.4937725	7.00E-06	0.000341
BFSP1	13.78029981	-5.957518989	1.325924756	-4.4931049	7.02E-06	0.000342
RDH10	87.04403962	-2.405870173	0.535559471	-4.4922559	7.05E-06	0.000342
AF131217.1	245.8585621	1.413516676	0.315344129	4.48245757	7.38E-06	0.000357
PPP2R5A	1008.455092	-1.27937252	0.28576967	-4.476936	7.57E-06	0.000366
RP11-445P17.3	9.868011786	-6.496157419	1.451910813	-4.4742124	7.67E-06	0.00037
TIAM1	61.56944927	-2.848208802	0.637044819	-4.4709708	7.79E-06	0.000373
LAMA2	80.02263622	-2.922134491	0.653546168	-4.4711983	7.78E-06	0.000373
SFMBT2	1815.913353	1.214592471	0.271906898	4.46694246	7.93E-06	0.00038
UNC93B1	128.8975486	1.596145562	0.357754438	4.46156747	8.14E-06	0.000388
RP11-93B14.10	36.18525667	2.303842819	0.51750842	4.45179775	8.52E-06	0.000405
ANKRD9	108.6471708	-2.44140331	0.548662503	-4.449736	8.60E-06	0.000408
RUNX2	417.9325221	2.132630394	0.480001575	4.44296541	8.87E-06	0.00042
ZNF229	26.37757627	3.242090074	0.730166223	4.44020823	8.99E-06	0.000425
KIR3DL2	68.88684234	-2.303106376	0.519035945	-4.4372772	9.11E-06	0.00043
CTD-2532D12.4	10.10479562	-6.54519728	1.475250434	-4.4366686	9.14E-06	0.00043
RP11-81H14.2	167.2730194	-2.318063184	0.522743966	-4.434414	9.23E-06	0.000433
FHAD1	27.18198368	-2.974445554	0.67187074	-4.4271098	9.55E-06	0.000447
FAM167A	63.86181141	2.721937292	0.615404873	4.4230025	9.73E-06	0.000455
TRPV3	27.02384534	-3.204094371	0.725710423	-4.4151142	1.01E-05	0.00047
NANOGP11	9.455855103	-6.443023076	1.460282163	-4.4121768	1.02E-05	0.000476
FASLG	685.9795706	1.288002023	0.292109913	4.40930611	1.04E-05	0.000481
MIR600HG	112.9220142	1.716835637	0.389491933	4.40788497	1.04E-05	0.000483
TNR	138.9783084	2.603347823	0.591226144	4.40330295	1.07E-05	0.000492
KLF7	169.2487068	-2.312800422	0.525652201	-4.3998682	1.08E-05	0.000499

C2CD2	235.2098077	1.626141398	0.369926432	4.39585079	1.10E-05	0.000507
GLIS3	16.93045173	4.149400229	0.9464183	4.38431952	1.16E-05	0.000533
ZNF667-AS1	24.26880799	3.071377889	0.701007373	4.38137744	1.18E-05	0.000539
ATP1B1	80.98261209	1.826033102	0.417285823	4.37597685	1.21E-05	0.000552
CH17-373J23.1	61.27987687	3.193672111	0.730052534	4.37457849	1.22E-05	0.000554
CDK5R1	542.8694066	1.65236327	0.377786709	4.37379938	1.22E-05	0.000555
CDYL	800.2088148	1.300839967	0.297710473	4.36948004	1.25E-05	0.000564
RPS20P31	12.35087581	-5.828476142	1.333965396	-4.3692859	1.25E-05	0.000564
ZHX3	98.78087341	-1.81546185	0.416017502	-4.3639074	1.28E-05	0.000576
PATL2	498.7663474	-1.7095743	0.392165788	-4.3593152	1.30E-05	0.000587
RP11-23P13.6	19.82513079	-3.667644254	0.84316028	-4.3498779	1.36E-05	0.000612
CXorf40A	318.4527688	1.564026106	0.360201114	4.34209125	1.41E-05	0.000632
SATB2	12.52384386	-5.942039934	1.371583341	-4.3322485	1.48E-05	0.00066
PRSS30P	72.00890864	-2.287026043	0.52798791	-4.3315879	1.48E-05	0.00066
LINC00484	62.73452305	-2.533300719	0.584924674	-4.3309862	1.48E-05	0.000661
LINC00963	80.11884588	-1.983015317	0.458284904	-4.3270361	1.51E-05	0.000668
FOSB	8842.34726	1.209890337	0.279674945	4.32605908	1.52E-05	0.000668
ADAM28	327.1411681	1.478044642	0.341554498	4.327405	1.51E-05	0.000668
RGS1	3275.607839	1.470230612	0.339791673	4.32685886	1.51E-05	0.000668
OR7E13P	9.027775351	-6.375069231	1.473526986	-4.3264014	1.52E-05	0.000668
HLA-DPB1	212.2175029	-1.998019674	0.46236474	-4.3213063	1.55E-05	0.000681
CTD-2020K17.1	264.585717	1.425900741	0.330080619	4.319856	1.56E-05	0.000684
LINC00092	22.37145372	-3.165577245	0.73355499	-4.3153919	1.59E-05	0.000696
RNVU1-19	91.68292277	2.630430465	0.609685129	4.31440811	1.60E-05	0.000698
ZNF600	284.0729572	-1.44739615	0.336205665	-4.3050915	1.67E-05	0.000726
RP11-4B16.3	35.12523268	-2.7265624	0.63340525	-4.3046097	1.67E-05	0.000726
ARHGEF11	50.26554864	2.305335121	0.535935141	4.30151887	1.70E-05	0.000735
GOLGA7B	16.28004853	-4.296709466	1.002013611	-4.288075	1.80E-05	0.000779
STK17A	2220.252589	1.111276756	0.259256715	4.28639526	1.82E-05	0.000783
STARD9	1381.601549	1.257029975	0.2941782	4.27302218	1.93E-05	0.00083
SPIRE1	36.86738986	-2.366733887	0.55400368	-4.2720545	1.94E-05	0.000832
RP11-730K11.1	44.51684045	-2.292162657	0.536905047	-4.2692142	1.96E-05	0.000841
BACH2	124.3972019	-2.26743246	0.531713729	-4.2643858	2.00E-05	0.000857
CARMIL2	264.4363984	1.448756269	0.340084845	4.25998479	2.04E-05	0.000872
STAMBPL1	326.7858405	1.314946571	0.309054019	4.25474671	2.09E-05	0.000891
RP3-471M13.2	20.54576697	4.102091953	0.964235554	4.25424258	2.10E-05	0.000891
FAM110C	14.76145367	-4.812768268	1.133777328	-4.2448973	2.19E-05	0.000927
NEK3	24.89776855	-3.042745481	0.717374612	-4.2415015	2.22E-05	0.000939
RP11-753H16.5	8.842053049	-6.359414762	1.502480002	-4.2326119	2.31E-05	0.000975
AC125421.1	8.836095658	-6.357033648	1.502861407	-4.2299534	2.34E-05	0.000985
GAS1	18.35182187	-3.338307768	0.789568036	-4.2280179	2.36E-05	0.000991
IGF2R	3822.489397	-1.307396574	0.309494339	-4.2242988	2.40E-05	0.001005
CA5B	252.587242	-1.29161259	0.306216731	-4.2179687	2.47E-05	0.001031
KCNQ5	197.8572306	1.408049292	0.333850664	4.21760219	2.47E-05	0.001031
C17orf51	12.91700359	-5.085276235	1.20701887	-4.2130876	2.52E-05	0.00105
AATK	220.443926	1.572211323	0.373309101	4.21155369	2.54E-05	0.001055
CRACR2B	36.3687875	2.256889787	0.536536288	4.20640661	2.59E-05	0.001077
ADAMTS14	27.70545909	-3.437971257	0.818012761	-4.2028333	2.64E-05	0.001092

ARHGAP42	48.48524445	-2.314001391	0.550942311	-4.2000793	2.67E-05	0.001103
GPR82	101.9675273	2.083420227	0.49623937	4.19841785	2.69E-05	0.001109
RASSF3	349.4592184	-1.22217939	0.29125024	-4.1963206	2.71E-05	0.001114
SIGLEC17P	207.1442542	1.573605831	0.374965975	4.196663	2.71E-05	0.001114
LTA	23.34819955	3.561644178	0.848887674	4.19566014	2.72E-05	0.001115
MX2	131.2776059	-1.620427447	0.386674921	-4.1906712	2.78E-05	0.001138
ZDBF2	220.4842759	1.749288815	0.417693914	4.18796817	2.81E-05	0.001149
RAB11FIP5	72.36389504	-2.663069692	0.636255703	-4.1855337	2.84E-05	0.001159
PPP2R5C	3524.479885	-1.211026028	0.289770876	-4.1792538	2.92E-05	0.001189
ZMAT4	12.77235446	-5.91999979	1.417959956	-4.175012	2.98E-05	0.001209
BCO2	203.326195	2.077606356	0.498347249	4.16899333	3.06E-05	0.001239
CCRL2	58.27406769	2.065352387	0.495985526	4.16413843	3.13E-05	0.001263
STX11	243.980111	1.43737758	0.345579678	4.15932322	3.19E-05	0.001287
ZYG11B	128.8206961	-1.556768477	0.374334193	-4.1587664	3.20E-05	0.001287
CCNB3	16.04212477	-4.435123802	1.066975012	-4.156727	3.23E-05	0.001296
SVIL	116.5889823	-2.220386938	0.534414622	-4.154802	3.26E-05	0.001305
LINC01504	18.67656839	-4.159376689	1.001321975	-4.1538854	3.27E-05	0.001307
ARMCX2	38.30196903	2.247971547	0.541381398	4.15228812	3.29E-05	0.001314
LINC00996	103.2511827	1.698511335	0.409501087	4.14775781	3.36E-05	0.001337
SLC2A1	331.9063987	-1.489350739	0.359834192	-4.1389917	3.49E-05	0.001387
NUDT14	72.5924146	2.008785494	0.485817355	4.13485742	3.55E-05	0.001409
AIG1	63.94207301	1.791091061	0.433637286	4.13038989	3.62E-05	0.001431
ENO2	26.16185771	-2.897058119	0.701373376	-4.1305505	3.62E-05	0.001431
PRSS23	493.1202428	-5.139354163	1.244680751	-4.1290541	3.64E-05	0.001436
PDE9A	15.21774153	3.355763961	0.813636623	4.12440132	3.72E-05	0.001463
ANO9	130.5649927	1.810587534	0.439329923	4.12124793	3.77E-05	0.00148
RELL1	226.6832299	1.417498122	0.344391216	4.11595318	3.86E-05	0.001511
KIR2DS4	91.9790384	-4.998573862	1.215141108	-4.1135748	3.90E-05	0.001524
AC021188.4	27.1728243	-2.543034179	0.618415889	-4.1121747	3.92E-05	0.001528
MDFIC	498.7926078	1.336630947	0.325051151	4.11206342	3.92E-05	0.001528
CLDND1	862.3970219	1.367084039	0.332760753	4.10830912	3.99E-05	0.00155
MGAT4A	2182.002369	-1.295428434	0.315710507	-4.1032161	4.07E-05	0.001581
PCDH1	87.27708207	-5.538864962	1.350634443	-4.1009357	4.11E-05	0.001594
MSX2P1	12.07354186	-5.078119283	1.239820549	-4.0958502	4.21E-05	0.001626
MACC1	23.06515008	2.639861031	0.645132251	4.09196878	4.28E-05	0.00165
KCNA3	206.3412346	-1.804104564	0.441209313	-4.0889993	4.33E-05	0.001668
AMOTL1	61.09054111	-2.310137186	0.566188989	-4.0801521	4.50E-05	0.00173
CELSR2	58.08795737	-2.339891073	0.574790392	-4.0708598	4.68E-05	0.001794
KLF2	1129.446113	-1.615895519	0.396944316	-4.0708368	4.68E-05	0.001794
ARHGAP8	12.47137101	-5.091748658	1.253013183	-4.0636034	4.83E-05	0.001843
AHR	386.8588191	1.619661018	0.398556421	4.06381865	4.83E-05	0.001843
TXNIP	13492.16443	-1.814227654	0.446520105	-4.0630369	4.84E-05	0.001844
KSR1	571.2718587	1.532842178	0.377420129	4.06136838	4.88E-05	0.001853
ESR1	106.6219432	-1.507979231	0.371586062	-4.0582233	4.94E-05	0.001875
ANKDD1A	116.8184675	1.715301924	0.42292793	4.0557783	5.00E-05	0.001891
TRAF5	959.2000046	1.105285004	0.272945448	4.04947221	5.13E-05	0.001939
HOPX	572.5028046	-1.732827079	0.429998842	-4.0298413	5.58E-05	0.002104
EFHD2	1994.20591	-1.229877664	0.305573366	-4.0248196	5.70E-05	0.002146

RP11-305L7.1	31.56062642	-2.389042746	0.59398336	-4.0220702	5.77E-05	0.002167
DLEC1	56.25903188	-2.033519189	0.505793657	-4.0204521	5.81E-05	0.002178
BIN1	412.7167661	-1.264437064	0.314774884	-4.0169567	5.90E-05	0.002206
DISC1	127.0296083	1.578799608	0.393268921	4.01455473	5.96E-05	0.002224
GGT7	25.3359325	-2.744042291	0.684946979	-4.0062112	6.17E-05	0.0023
RP11-81H14.1	49.54669768	-2.481334287	0.619829996	-4.0032498	6.25E-05	0.002325
INPP5B	630.6455232	1.118801196	0.279522144	4.00254942	6.27E-05	0.002327
TNFAIP8L1	148.4750774	1.438044252	0.35943715	4.00082255	6.31E-05	0.00234
MCM6	177.446596	-1.755672417	0.439299338	-3.9965287	6.43E-05	0.002378
SPN	1840.619884	-1.069866811	0.267906411	-3.9934349	6.51E-05	0.002405
NECTIN1	23.15694264	-3.336068125	0.835537748	-3.9927198	6.53E-05	0.002408
CMTM7	116.1062743	1.880803862	0.47119236	3.99158395	6.56E-05	0.002415
HLA-DMB	112.2398926	1.618418729	0.405736118	3.9888456	6.64E-05	0.002438
C16orf45	15.65260087	-3.384643705	0.84895939	-3.9868146	6.70E-05	0.002455
TRAC	158.7453762	1.775529799	0.446832668	3.97358995	7.08E-05	0.002589
TOB1	153.0483151	-1.801607953	0.453433238	-3.9732596	7.09E-05	0.002589
LINC00944	26.99727998	-3.831916894	0.966771995	-3.9636201	7.38E-05	0.002691
OR7E2P	7.853102008	-6.193462259	1.563082563	-3.9623385	7.42E-05	0.0027
ZNF365	12.36147154	-4.279780172	1.080769074	-3.9599395	7.50E-05	0.002723
RP11-736K20.4	14.10389364	-4.480961669	1.131759252	-3.9592887	7.52E-05	0.002725
BCL2A1	124.726138	1.542111132	0.390341876	3.95066793	7.79E-05	0.00282
ARRB2	396.3709634	-1.139838069	0.288754022	-3.9474362	7.90E-05	0.002853
SYNRG	1833.131838	1.070168026	0.271881853	3.936151	8.28E-05	0.002985
RCAN3	92.76134775	1.645521668	0.418568546	3.93130751	8.45E-05	0.00304
MEIS1	26.81344668	-2.858132542	0.728347843	-3.9241313	8.70E-05	0.003127
MYC	65.74177695	1.812834337	0.462350758	3.92090703	8.82E-05	0.003163
RP11-1055B8.4	17.65302623	-3.727505454	0.952919677	-3.911668	9.17E-05	0.003281
CYTH3	252.542155	1.316774452	0.336838199	3.90921949	9.26E-05	0.003308
PLXNA2	15.67224392	-3.892514806	0.996164697	-3.9075013	9.33E-05	0.003326
RPL34P20	34.30917405	2.212781988	0.567300197	3.90054859	9.60E-05	0.003416
PCDHGA11	13.93659102	-3.630764132	0.931576306	-3.8974415	9.72E-05	0.003454
ASB2	103.7215806	1.437187172	0.368959328	3.89524553	9.81E-05	0.003479
CTD-2554C21.1	20.76757685	2.972342319	0.763953569	3.89073687	9.99E-05	0.003538
DOCK5	708.5670373	-4.407326284	1.134356595	-3.8853094	0.000102	0.003612
SIGLEC5	6.146843422	6.300311342	1.622400562	3.88332665	0.000103	0.003635
RN7SL443P	9.985632763	4.586883174	1.181942478	3.88080068	0.000104	0.003653
ARMC9	26.7727256	2.743985535	0.7070158	3.88108092	0.000104	0.003653
RP11-75C9.2	6.315772186	6.336890894	1.632595721	3.88148199	0.000104	0.003653
NUAK1	89.03415783	-4.923768173	1.269853032	-3.8774315	0.000106	0.003698
GIPR	49.90489659	-2.271641229	0.587837868	-3.864401	0.000111	0.003894
CH507-513H4.5	19.0567903	3.745700553	0.972859097	3.85019841	0.000118	0.00412
CNN2	593.4786396	1.322235009	0.343668712	3.84741166	0.000119	0.004159
SNX10	242.3358568	1.279673082	0.332794882	3.84523066	0.00012	0.004185
DLL1	87.36929126	1.658729668	0.431392826	3.84505622	0.000121	0.004185
PROCR	26.45366543	-2.805026259	0.729650451	-3.8443425	0.000121	0.00419
PRR5L	1326.674747	-1.234868418	0.321434033	-3.8417476	0.000122	0.00419
CLPTM1	411.5278394	1.34292923	0.34950235	3.84240401	0.000122	0.00419
IFRD1	2699.957488	1.546278988	0.402491488	3.84176817	0.000122	0.00419

MTHFD2	163.1876685	-1.525370396	0.397019633	-3.8420528	0.000122	0.00419
LFNG	399.2252516	-1.59868108	0.416065683	-3.8423767	0.000122	0.00419
RP11-54O7.17	17.44336897	-3.764751791	0.979707848	-3.842729	0.000122	0.00419
RP11-435O5.6	10.52049164	-4.807950681	1.252268823	-3.8393918	0.000123	0.004223
PABPC1	3257.461736	1.166386588	0.304082885	3.83575218	0.000125	0.004279
RP11-298J23.5	11.9087372	-3.999220202	1.043491849	-3.8325361	0.000127	0.004305
U91324.1	32.27623017	-2.521088311	0.657804027	-3.8325827	0.000127	0.004305
TBC1D30	34.52274449	-2.188673158	0.571073011	-3.8325628	0.000127	0.004305
RP11-351D16.3	22.48772635	-2.704422305	0.70564253	-3.832567	0.000127	0.004305
ATF3	190.7941236	-1.762555728	0.459959472	-3.8319805	0.000127	0.004308
MICU3	59.13338922	1.737360905	0.453494993	3.8310476	0.000128	0.004317
MORN3	29.24902237	-2.236271213	0.584945518	-3.8230419	0.000132	0.004452
STN1	371.8815694	1.191965974	0.312226441	3.81763303	0.000135	0.004542
ATP11A	171.2523947	-1.675583948	0.439299068	-3.8142215	0.000137	0.004598
RP11-1105O14.1	8.243982175	-6.268260315	1.643607537	-3.8137208	0.000137	0.004599
LILRP1	7.216270092	-6.00472638	1.578242291	-3.8046924	0.000142	0.004762
ZBTB44	1247.08483	-1.097954423	0.288959464	-3.7996832	0.000145	0.004851
HDAC7	768.5624003	1.061752137	0.279493383	3.79884534	0.000145	0.004852
TPK1	99.11873832	-1.522431898	0.400768119	-3.7987849	0.000145	0.004852
RP11-736K20.5	6.609111179	-5.926249434	1.561025111	-3.7963832	0.000147	0.004883
ARHGAP31	251.1225738	1.388145972	0.365639615	3.7964868	0.000147	0.004883
RP11-413E1.4	13.40928077	-3.733891202	0.984273041	-3.7935522	0.000149	0.004931
KCNQ1OT1	291.0707352	-1.232437835	0.325121743	-3.7906964	0.00015	0.00498
GCNT4	6.891303069	-6.005539856	1.584846771	-3.7893505	0.000151	0.004998
HES6	76.86181731	-1.837370916	0.485117397	-3.7874769	0.000152	0.005028
ZNF662	17.7849954	3.575613556	0.944347718	3.78633155	0.000153	0.00504
FCGR2B	34.97465317	-3.051624333	0.806015549	-3.7860614	0.000153	0.00504
TMTC4	65.69405615	-1.589923334	0.420262869	-3.783164	0.000155	0.00509
TPST2	866.7897482	-1.208960415	0.319760775	-3.7808278	0.000156	0.005125
ZCCHC24	78.09141368	1.520512577	0.402181723	3.78066056	0.000156	0.005125
MXD1	366.4951192	-1.324070455	0.35092991	-3.7730339	0.000161	0.005275
LINC00469	6.571609621	-5.914799814	1.568023061	-3.7721383	0.000162	0.005285
IL10RA	2148.304468	-1.311007704	0.347597299	-3.771628	0.000162	0.005287
ELMO1	394.5386972	-1.185553481	0.314444052	-3.7703161	0.000163	0.005306
SLC35E1	494.0453718	1.341331033	0.356605986	3.76138115	0.000169	0.005491
MICAL2	717.0636258	1.434424715	0.381618762	3.75878981	0.000171	0.005539
ADRB2	880.089908	-1.225287349	0.32604899	-3.7579854	0.000171	0.005547
SESN1	857.331721	1.198612199	0.319145377	3.75569344	0.000173	0.005571
CD200R1	67.74310968	2.198311444	0.585298814	3.75587886	0.000173	0.005571
HNRNPA1P70	14.87149842	3.424336918	0.911669688	3.7561158	0.000173	0.005571
PCDHGB8P	11.60607838	-4.037033378	1.075129895	-3.7549262	0.000173	0.005579
IFNAR2	335.1719381	1.239914836	0.33056866	3.75085417	0.000176	0.005661
FAM102A	163.392481	1.287446427	0.344058393	3.74194164	0.000183	0.005856
ADD3	1796.254427	-1.228293062	0.328896712	-3.734586	0.000188	0.006011
IGFBP7	168.8582947	-4.568837711	1.223300171	-3.734846	0.000188	0.006011
SGCD	13.62454309	-4.236648183	1.134813633	-3.7333427	0.000189	0.006031
YBX3	34.24102106	-2.48240698	0.665086184	-3.7324591	0.00019	0.006042
ZNF101P2	21.97429443	-2.552751459	0.684665913	-3.7284629	0.000193	0.006129

TENM1	649.1631813	1.035230936	0.277762319	3.72703878	0.000194	0.006154
CCL25	13.38943681	3.591207682	0.963871472	3.72581593	0.000195	0.006174
LPIN1	1835.869185	1.162779475	0.312619635	3.71947039	0.0002	0.006321
C3AR1	61.00341117	-1.812484427	0.487423539	-3.7185	0.0002	0.006335
WEE1	318.640766	1.278210218	0.344154554	3.71405871	0.000204	0.006437
KCNC4	30.01462304	-2.32576914	0.626301194	-3.7134995	0.000204	0.006441
FEZ1	255.1175407	1.378286918	0.371286081	3.71219657	0.000205	0.006464
LINC00892	35.11558265	2.688604275	0.724901654	3.7089228	0.000208	0.006537
FZD8	19.87966583	-3.109399731	0.840102273	-3.7012157	0.000215	0.006729
COLQ	800.5898283	1.450538462	0.392472629	3.69589713	0.000219	0.00686
USP31	84.60943467	-1.446051753	0.391713669	-3.691604	0.000223	0.006966
RNF157	209.2207468	1.612889623	0.436960799	3.69115405	0.000223	0.006967
TMEM181	1261.969643	-0.973016796	0.26399211	-3.6857798	0.000228	0.007105
CCDC162P	20.00295842	-2.787879662	0.757426507	-3.6807263	0.000233	0.007236
GS1-115G20.2	45.78694506	-2.230820293	0.606389498	-3.6788571	0.000234	0.007277
MMP9	37.44072311	2.410775654	0.655639132	3.67698561	0.000236	0.00732
C10orf10	24.51562269	-2.472784171	0.672831433	-3.6751912	0.000238	0.00736
TKTL1	149.1948965	-2.188348688	0.595749372	-3.6732707	0.000239	0.007404
RP11-80H8.4	24.52712255	-2.752885338	0.750300076	-3.6690458	0.000243	0.007515
ATP2B1	913.6084668	-1.091706262	0.297683274	-3.6673416	0.000245	0.007554
CCR9	208.9932691	1.604538924	0.437709868	3.66575908	0.000247	0.007577
GPR34	28.13347947	2.220367169	0.60566387	3.66600565	0.000246	0.007577
PHACTR2	278.4157835	-1.472009274	0.402168849	-3.6601773	0.000252	0.007732
AP000654.4	6.425529869	-5.899874519	1.614158006	-3.6550787	0.000257	0.007875
HNRNPA1P31	20.53252062	2.50364192	0.685430116	3.65265818	0.00026	0.007937
IRF6	41.92307571	-2.102068168	0.576706088	-3.6449557	0.000267	0.008159
CXXC4	12.27672259	-4.097802967	1.124288001	-3.6447983	0.000268	0.008159
RP1-257C22.2	5.93496766	-5.761755279	1.581086211	-3.6441753	0.000268	0.008166
HOOK1	72.42713049	2.052178651	0.563534357	3.64162118	0.000271	0.008234
NEB	92.37068	-1.432652924	0.393526787	-3.6405474	0.000272	0.008256
CTB-133G6.1	83.62184758	-4.189817102	1.15176336	-3.6377413	0.000275	0.008334
MTM1	112.5793335	1.361859106	0.374477924	3.63668729	0.000276	0.008355
INPP5A	97.69431359	-1.832944287	0.504549348	-3.6328345	0.00028	0.008468
ADAM10	1858.771052	-1.075168428	0.29615744	-3.6303948	0.000283	0.008535
GARNL3	20.44339593	-2.661097908	0.733387099	-3.6285038	0.000285	0.008585
PABPC4	1008.089157	1.384480855	0.381748243	3.62668559	0.000287	0.008633
KLF9	462.4621063	-1.378414934	0.380818707	-3.6196093	0.000295	0.008859
KRTAP5-AS1	27.27781791	-2.547041782	0.704329381	-3.6162651	0.000299	0.00896
ANKRD20A11P	166.5296481	-5.154712828	1.42570454	-3.6155548	0.0003	0.008971
PARVG	516.8588873	0.990273811	0.273968272	3.61455654	0.000301	0.008992
BAHCC1	38.41336259	-2.696423845	0.747288981	-3.6082746	0.000308	0.009199
RP11-438B23.2	44.4979687	-2.224492411	0.61662549	-3.6075259	0.000309	0.009211
KCNQ5-IT1	35.5641756	1.888449679	0.523849076	3.60494991	0.000312	0.009289
RP11-214O1.2	91.9711049	-1.887341147	0.523619323	-3.6044146	0.000313	0.009294
PTGFRN	35.12531225	2.416655875	0.670550242	3.60398926	0.000313	0.009296
WDFY2	402.1668678	-1.019651377	0.283142629	-3.6011934	0.000317	0.009382
RRM2	39.4856204	-2.335809362	0.648889222	-3.5997044	0.000319	0.009422
MTHFD1L	41.99720889	1.869354521	0.519626602	3.59749581	0.000321	0.009488

RP11-467H10.2	14.45277978	-3.655762545	1.016668767	-3.5958246	0.000323	0.009535
MPP1	61.2774592	1.522813031	0.424189207	3.58993818	0.000331	0.009738
AC097713.4	8.694795904	-5.348637343	1.49223009	-3.5843248	0.000338	0.009925
GPR15	71.7831699	2.119045814	0.591216095	3.58421537	0.000338	0.009925
C2orf48	5.740653114	-5.709628287	1.594628623	-3.5805379	0.000343	0.010051
TANC1	558.2546435	3.155662729	0.882313803	3.5765764	0.000348	0.010174
PTPRD-AS1	11.82585468	3.526727821	0.98598779	3.57684736	0.000348	0.010174
HEG1	1725.170045	0.995746207	0.278639501	3.57360031	0.000352	0.010275
UBASH3B	564.2660856	1.127704964	0.315783221	3.57113643	0.000355	0.010357
PRMT9	278.7307551	1.145632561	0.321152481	3.56725428	0.000361	0.010496
RAB29	550.7787659	-1.062992123	0.29832396	-3.563214	0.000366	0.010643
CD47	2083.819326	-1.13948982	0.320276732	-3.5578289	0.000374	0.010848
IKZF3	5563.007635	1.066920745	0.300101785	3.55519626	0.000378	0.010925
AC004593.3	68.00735062	1.748540894	0.491806023	3.55534665	0.000377	0.010925
PARP3	89.62996921	1.464640911	0.412309088	3.55228869	0.000382	0.01103
CATSPER1	22.28363001	-2.472576872	0.696639208	-3.5492933	0.000386	0.01114
SPAG4	200.8171321	1.409823498	0.397527008	3.54648481	0.00039	0.011243
RP11-862L9.2	47.91274894	-2.018134345	0.569165369	-3.5457785	0.000391	0.011257
PLCG2	3296.470605	1.046530527	0.295264262	3.54438604	0.000394	0.0113
OSBPL2	843.0618569	0.933095462	0.263802101	3.53710398	0.000405	0.011589
CH507-42P11.8	118.4926675	1.729887774	0.489090152	3.53695074	0.000405	0.011589
RGS10	23.08125368	-2.418922927	0.685219456	-3.5301434	0.000415	0.011875
C11orf63	25.8146242	2.298261326	0.651176465	3.52939864	0.000417	0.011891
PTGER2	757.4352553	-1.108074119	0.314136508	-3.527365	0.00042	0.011965
YWHAH	304.2280503	1.338540981	0.379568661	3.52647918	0.000421	0.011988
CEBPA	29.2059735	-2.902816524	0.823516581	-3.5249036	0.000424	0.012042
HIVEP2	1026.809882	1.131226987	0.321216047	3.52170135	0.000429	0.012171
KRT2	13.73397377	-3.582502069	1.0182041	-3.5184518	0.000434	0.012303
KAT2B	1216.143443	-0.983953671	0.279711184	-3.5177488	0.000435	0.012318
VLDLR	45.54370524	-2.097335537	0.596590263	-3.5155377	0.000439	0.012404
KLF13	2584.471279	-1.111949568	0.316360975	-3.5148127	0.00044	0.01242
NDC80	32.47207675	-2.334934444	0.665400709	-3.5090652	0.00045	0.012673
ZFYVE28	318.558651	-1.578925774	0.450280984	-3.5065344	0.000454	0.012776
CXCR1	64.3978748	-5.239959512	1.496885413	-3.5005749	0.000464	0.013047
SURF4	1334.116026	1.264782493	0.361395603	3.49971744	0.000466	0.01307
PCNX2	108.1590252	-1.617562631	0.462329168	-3.498725	0.000467	0.013082
GSAP	2174.09228	-1.192658042	0.340880228	-3.4987598	0.000467	0.013082
SCMH1	123.3480363	1.268399266	0.362604201	3.49802695	0.000469	0.013097
PRKCB	1646.62345	-0.999381928	0.285788516	-3.4969282	0.000471	0.013132
ST7	33.78063387	-1.865631776	0.533561501	-3.4965637	0.000471	0.013132
CHST2	1342.549037	-1.650877533	0.472239815	-3.4958457	0.000473	0.013149
PHLDA1	194.3461047	-1.33524682	0.382050895	-3.4949449	0.000474	0.013175
GNAO1	19.9185251	-3.598196445	1.030447258	-3.4918783	0.00048	0.013293
TP53INP1	547.3281094	1.296168576	0.371201611	3.49181829	0.00048	0.013293
PODXL	37.26326114	-1.781301443	0.510336972	-3.4904417	0.000482	0.013324
SRPK2	662.4761147	-0.936561563	0.26830237	-3.4906943	0.000482	0.013324
DCBLD1	175.1049608	1.444075862	0.413991723	3.48817568	0.000486	0.013419
RRBP1	520.2817868	-1.128073668	0.323791728	-3.4839484	0.000494	0.013614

TIPARP	3201.56838	1.135838915	0.326522583	3.47859222	0.000504	0.01385
S1PR2	82.40873929	1.685944943	0.484646596	3.47870997	0.000504	0.01385
MT-ND6	61.42535317	2.543274626	0.731882362	3.47497734	0.000511	0.014018
SLC2A6	32.14417211	-2.227843293	0.641273199	-3.4740939	0.000513	0.014045
RP11-214O1.3	19.70712014	-2.837155424	0.816753953	-3.4736966	0.000513	0.014046
PGRMC2	258.4643982	1.126716155	0.324449687	3.47269915	0.000515	0.014079
OTOF	7.637099175	-5.235854282	1.510262527	-3.4668504	0.000527	0.014369
MTATP6P1	18.85188229	3.115706761	0.898842192	3.4663557	0.000528	0.014376
GPR68	384.5861412	1.222000266	0.352602047	3.46566413	0.000529	0.014393
CYBRD1	96.49600531	-4.365114656	1.260130889	-3.4640169	0.000532	0.01446
CTD-2017C7.1	35.00208287	-2.137668079	0.617168756	-3.4636687	0.000533	0.01446
MAGEF1	57.44259211	-1.67217411	0.48309013	-3.4614123	0.000537	0.014562
PACSIN1	6.942256741	-5.063434595	1.463539485	-3.4597185	0.000541	0.014634
SH2D3A	76.62330033	1.58112501	0.457598551	3.45526665	0.00055	0.014837
LINC00539	37.83509753	1.88321142	0.545000227	3.45543236	0.000549	0.014837
CHKA	222.5719555	1.41079237	0.408558337	3.45309896	0.000554	0.014937
DENND4A	1836.01788	0.891794259	0.258508568	3.44976673	0.000561	0.015102
BCL9L	421.5562256	-1.363565378	0.395345398	-3.4490483	0.000563	0.015121
KCTD5	260.1397774	1.094682726	0.317826818	3.44427425	0.000573	0.01537
VMP1	765.4684671	-1.206616031	0.350571397	-3.4418553	0.000578	0.015487
LRIT2	9.829901021	3.820292972	1.110401849	3.44045984	0.000581	0.015546
AK5	80.62098828	-4.056625438	1.179594727	-3.4389993	0.000584	0.015609
CTD-2060C23.1	5.658541271	-5.664406158	1.647334333	-3.4385286	0.000585	0.015615
P2RY11	124.6864791	1.251954341	0.364611557	3.43366609	0.000595	0.015855
IL12RB2	1132.083831	1.35835306	0.395567108	3.43393835	0.000595	0.015855
NFATC2	1627.139066	-1.018514724	0.297175105	-3.4273218	0.00061	0.016208
EDARADD	8.373233509	-4.661264869	1.360985399	-3.4249191	0.000615	0.01633
ERFE	17.46399497	-2.585902958	0.755447667	-3.4230074	0.000619	0.016423
VANGL1	246.4796447	1.157261177	0.338375026	3.42005494	0.000626	0.01658
GALC	671.3597966	-1.128432881	0.330270359	-3.4166944	0.000634	0.016764
LMNA	240.2703728	-1.890835496	0.553581438	-3.4156411	0.000636	0.016806
HIPK1-AS1	265.1789497	1.398616612	0.409918513	3.41193815	0.000645	0.017014
HSPA8P4	7.503085995	-5.093726355	1.49465632	-3.4079583	0.000655	0.017241
BEND3P1	27.49451825	2.264789447	0.664993115	3.40573368	0.00066	0.017359
TRPC3	10.2659155	-4.746279574	1.394882375	-3.4026379	0.000667	0.017533
RP11-69E11.8	61.96600415	1.559374102	0.458525137	3.40084758	0.000672	0.017625
SIPA1L1	952.9646283	1.198258265	0.352511158	3.39920663	0.000676	0.017707
CASP8	1001.249446	-0.99148645	0.291918978	-3.396444	0.000683	0.017863
RP11-864I4.3	14.85789468	-3.236399211	0.953126791	-3.39556	0.000685	0.017898
LINC00943	28.10752872	-3.008407241	0.886142407	-3.3949478	0.000686	0.017908
GCLM	172.64561	-1.14066219	0.336015472	-3.3946716	0.000687	0.017908
HMGA2	26.76641201	2.040957092	0.60139314	3.39371528	0.00069	0.017947
SPATS2L	46.95508098	1.548237988	0.456491085	3.39160619	0.000695	0.018062
RN7SL748P	8.795602307	-4.462881627	1.316512228	-3.389928	0.000699	0.018149
S1PR4	288.7476373	-1.218247901	0.359479193	-3.3889247	0.000702	0.018168
LRP12	71.03983875	1.390840887	0.410371308	3.38922547	0.000701	0.018168
MAPK3	114.4865806	1.192282545	0.35190794	3.38805241	0.000704	0.018202
CARNS1	125.9594485	-1.50944244	0.445566344	-3.387694	0.000705	0.018202

UACA	66.97122452	1.683058884	0.497944829	3.38001077	0.000725	0.01867
HPSE	15.42402657	-3.462214986	1.024227264	-3.3803191	0.000724	0.01867
S100PBP	1063.4098	0.99328226	0.294197187	3.37624663	0.000735	0.018894
HLA-DRB1	133.4977633	-1.70688283	0.505591442	-3.3760121	0.000735	0.018894
TXNRD3	44.27031565	1.821175278	0.539729732	3.3742356	0.00074	0.018992
OPHN1	61.6087124	-1.733375771	0.51415359	-3.371319	0.000748	0.019169
DUSP8	54.26779974	-2.47166128	0.734654406	-3.3643864	0.000767	0.019632
SAMHD1	1560.229499	-1.150919877	0.342523539	-3.3601191	0.000779	0.019912
CTA-292E10.6	136.2548704	1.283774814	0.38247339	3.35650754	0.000789	0.020147
CCDC112	10.00200928	-3.595377401	1.071742342	-3.3547031	0.000795	0.020253
RGS12	27.09478698	-2.926968613	0.872714306	-3.353868	0.000797	0.020288
SLC25A20	295.615079	-1.108530566	0.330885377	-3.3501951	0.000808	0.020533
IRAK2	360.0836227	1.155162571	0.344988978	3.34840428	0.000813	0.020639
GLCCI1	737.7445408	0.97280885	0.290846708	3.34474767	0.000824	0.020886
MLC1	629.6383686	-0.999992267	0.29916659	-3.3425934	0.00083	0.020995
PPFIA3	13.52945839	-3.092545253	0.925130658	-3.34282	0.000829	0.020995
STX7	402.3398216	-1.202423851	0.359909984	-3.3409016	0.000835	0.021097
PDXK	488.4970881	0.99423569	0.297633378	3.34047108	0.000836	0.021102
VIT	5.674050352	-5.709344999	1.709717786	-3.3393494	0.00084	0.021161
ENTPD1	222.381585	1.62417226	0.486860029	3.33601479	0.00085	0.021389
CD81	1083.619061	1.094175547	0.328353116	3.33231357	0.000861	0.02162
AXIN1	847.2866719	-1.40532067	0.421686081	-3.3326229	0.00086	0.02162
LARGE1	16.04819682	-3.004609516	0.902277907	-3.3300267	0.000868	0.021771
SLITRK5	5.11963225	-5.517561378	1.658238792	-3.3273624	0.000877	0.021953
TGFBR3	1936.737911	-0.922983559	0.277527557	-3.3257366	0.000882	0.021966
CHPT1	176.3249387	1.45198772	0.436503848	3.32640302	0.00088	0.021966
FMNL3	1016.816928	1.013328013	0.304720368	3.32543577	0.000883	0.021966
ZNF528-AS1	40.00957286	1.867237615	0.561490714	3.32550044	0.000883	0.021966
UBE2F	240.9959224	-1.257495743	0.37798673	-3.3268251	0.000878	0.021966
PCID2	282.9661204	-0.977151473	0.293906489	-3.3247019	0.000885	0.021996
TBX1	6.436457212	-4.943000028	1.487549098	-3.3229155	0.000891	0.022109
PARP9	373.935695	-0.997730525	0.300475444	-3.320506	0.000899	0.022273
GNS	816.4935322	-0.987096633	0.297309548	-3.3200973	0.0009	0.022274
SLFN13	2450.135107	0.946798325	0.28519827	3.31978986	0.000901	0.022274
ABHD15	801.8555453	1.230662223	0.370948572	3.31760874	0.000908	0.022421
PLK3	85.16867576	-1.313817432	0.396213449	-3.3159335	0.000913	0.022528
CD69	6118.789048	1.102258101	0.332599038	3.31407484	0.000919	0.02265
MAPK7	73.8541402	-1.456520811	0.440471628	-3.3067301	0.000944	0.023223
UNG	222.6027477	1.325823939	0.401270931	3.30406176	0.000953	0.023416
GPD1L	424.0935015	1.072475272	0.324687904	3.30309586	0.000956	0.023438
ZSCAN1	6.268995741	5.516338117	1.669987537	3.30322113	0.000956	0.023438
SH3BGRL3	197.2398897	-1.198957969	0.363129333	-3.3017381	0.000961	0.023523
FN3K	37.16199523	1.749746579	0.530116915	3.30068053	0.000965	0.023583
SLCO3A1	174.7602764	-1.701593707	0.516565731	-3.2940507	0.000988	0.024116
RP1-15D23.2	10.8824573	3.37482427	1.025214395	3.29182294	0.000995	0.024278
RNF122	138.4732019	1.175389283	0.357147916	3.29104337	0.000998	0.024315
RP11-359K18.4	18.46044609	-2.509498712	0.762705915	-3.2902573	0.001001	0.024353
ACP6	164.9327529	1.479787046	0.449929799	3.28892874	0.001006	0.024439

UST	284.4312508	1.258326452	0.383036919	3.28513099	0.001019	0.02474
SCX	36.71136343	2.291739383	0.697741225	3.28451194	0.001022	0.024751
ZNF853	115.9732582	1.179476108	0.359124535	3.28430946	0.001022	0.024751
RP11-107E5.3	708.5152143	-1.40224883	0.427143777	-3.2828497	0.001028	0.024849
RP11-107E5.4	83.74068631	-1.748908476	0.533028128	-3.281081	0.001034	0.024975
SMIM24	8.174593367	4.622153629	1.408885111	3.28071721	0.001035	0.024977
CTC-506B8.1	8.621740197	-4.013237735	1.225186482	-3.2756138	0.001054	0.025402
CD38	1209.205809	0.957508371	0.292363615	3.27505996	0.001056	0.02542
RP11-864I4.4	13.15433275	-3.419782424	1.044614758	-3.2737259	0.001061	0.02551
PCSK6	24.97572191	2.863828823	0.877154113	3.26490953	0.001095	0.026285
ADAM12	32.28123139	-1.905381497	0.583882006	-3.2632989	0.001101	0.026403
RP11-39H3.2	5.323114708	-5.617252238	1.723694018	-3.2588454	0.001119	0.026724
RNU1-120P	5.287832609	5.280183237	1.620198032	3.25897399	0.001118	0.026724
BAZ2B	1417.795117	1.08433858	0.332697497	3.25923276	0.001117	0.026724
PDCD4	2092.231113	1.095670031	0.336255755	3.25844247	0.00112	0.026729
JAZF1	256.6649725	-1.05047167	0.322493415	-3.257343	0.001125	0.026801
ABCD2	15.7577182	-3.36659213	1.0346003	-3.2540027	0.001138	0.027085
LRRC8A	313.0160462	-1.059429169	0.326101906	-3.2487672	0.001159	0.027555
LINC00384	5.249978887	-5.606981497	1.726745117	-3.247139	0.001166	0.02768
ADRB1	66.74433272	1.868897574	0.575692832	3.24634505	0.001169	0.027698
ZNF154	345.6036921	1.230949275	0.379188985	3.24626854	0.001169	0.027698
C10orf11	21.67666926	2.073593438	0.638891627	3.2456106	0.001172	0.027729
RP11-413E1.2	9.863906006	-3.489088941	1.076399969	-3.2414428	0.001189	0.028104
SLC24A3	3.995777949	5.733133779	1.770236627	3.23862567	0.001201	0.028349
JAML	133.7218907	1.550378404	0.479718762	3.23184859	0.00123	0.028996
CYP4F35P	7.991481439	-3.975171157	1.230307568	-3.2310385	0.001233	0.029043
ERGIC1	1278.183439	1.052202471	0.326209531	3.22554178	0.001257	0.029572
RPL21	212.1326477	1.096053278	0.34019213	3.22186548	0.001274	0.029918
SGO1	25.14663842	-2.195255874	0.681678162	-3.22037	0.00128	0.030039
SLC25A4	79.64201018	-1.510327144	0.470391328	-3.2107887	0.001324	0.031022
PPP1R26	66.52614762	1.443363372	0.449677045	3.20977774	0.001328	0.031057
SLC6A6	522.827118	0.886287565	0.276096468	3.21006484	0.001327	0.031057
UOX	5.289621438	-5.613984083	1.750592753	-3.2069047	0.001342	0.031332
RP11-640L9.2	31.23376866	-1.899536759	0.592795172	-3.2043729	0.001354	0.031572
ARF4P4	16.62130025	2.485104332	0.775915449	3.202803	0.001361	0.031707
WDR27	315.840991	0.962585343	0.300607217	3.2021365	0.001364	0.031743
ECHDC3	6.718613243	4.126755586	1.28947136	3.20034683	0.001373	0.031903
FBP1	46.20358513	1.485076967	0.464184331	3.19932593	0.001377	0.031979
EPS8L2	35.06953832	1.624649705	0.507969124	3.19832374	0.001382	0.032028
PCDHGB5	22.01625623	-2.045302637	0.639516417	-3.1982019	0.001383	0.032028
TTC22	105.3106534	-1.250278034	0.3910193	-3.1974842	0.001386	0.03207
SEMA6C	18.54390387	-2.601265536	0.814088963	-3.1953087	0.001397	0.032258
HEMGN	10.10289577	3.810347463	1.192550302	3.19512515	0.001398	0.032258
RAB38	17.18356582	-2.566167817	0.803553284	-3.1935254	0.001405	0.0324
YPEL5	4897.003358	1.30205303	0.408217838	3.18960346	0.001425	0.032804
CTB-91J4.1	11.2630347	-3.487124889	1.094421503	-3.1862723	0.001441	0.033146
FOS	21800.79065	1.25182993	0.39301279	3.18521423	0.001446	0.033228
CARD16	118.8016498	-1.439957781	0.453759781	-3.1733923	0.001507	0.034572

IQGAP2	2935.198658	-0.845452275	0.266597034	-3.1712741	0.001518	0.034784
SFRP4	5.247344034	-5.58100989	1.760508718	-3.170112	0.001524	0.034883
EIF4G3	898.1424133	-0.914401144	0.288585295	-3.1685646	0.001532	0.03497
MZF1	228.4922023	1.112205897	0.351144406	3.16737467	0.001538	0.03497
OTULIN	1500.885562	0.994480848	0.313926952	3.16787342	0.001536	0.03497
ZBTB10	1544.148106	1.033955387	0.326398311	3.16777187	0.001536	0.03497
NRXN2	7.154577879	-4.942355157	1.560198464	-3.1677734	0.001536	0.03497
METTL9	696.0070555	0.963750248	0.304271989	3.1673972	0.001538	0.03497
PGM5	11.27386029	3.19354954	1.009958383	3.16206053	0.001567	0.035574
SLC37A1	325.1613982	1.132333215	0.358218415	3.16101341	0.001572	0.035661
ERRFI1	51.39917557	-2.040578004	0.64588856	-3.1593345	0.001581	0.035785
ZNF415	27.60187297	1.94640208	0.616044608	3.15951484	0.00158	0.035785
OBSCN	351.8437774	1.174804549	0.372076845	3.15742451	0.001592	0.035979
KIT	14.15736641	-2.758200064	0.874685231	-3.153363	0.001614	0.036441
GSTP1	944.6763203	1.003519563	0.318271029	3.15303459	0.001616	0.036441
ZNF609	1551.425636	0.916061005	0.29059123	3.15240417	0.001619	0.036478
MZF1-AS1	68.10393943	1.598898081	0.507477255	3.15067929	0.001629	0.036652
ZDHHC3	503.0418308	0.87840028	0.278910832	3.14939465	0.001636	0.036772
HIST1H1D	655.0458888	-1.031283249	0.327538162	-3.1485896	0.001641	0.036832
RAP1B	987.9067763	-0.923476914	0.293345941	-3.1480814	0.001643	0.036854
ABCD1	172.3507256	1.230597962	0.39110238	3.14648548	0.001652	0.037001
AKAP12	59.14721951	1.557336942	0.4950331	3.14592487	0.001656	0.037001
AC006369.2	6.638828107	-4.903424377	1.558511703	-3.1462224	0.001654	0.037001
RNA5SP390	4.422153029	-5.318490813	1.692087128	-3.1431542	0.001671	0.03731
RNF130	151.0964104	-1.080768032	0.343895615	-3.1427212	0.001674	0.037323
ATP10D	592.0707002	0.876602062	0.279582554	3.13539615	0.001716	0.038188
XPNPEP2	7.698152946	-4.31760463	1.377363149	-3.1346886	0.00172	0.038188
HIST4H4	448.1479891	1.093426417	0.348771535	3.13507929	0.001718	0.038188
LIPC-AS1	5.245888793	-5.614145574	1.79096887	-3.1346975	0.00172	0.038188
MIR7845	8.173248066	-3.73643248	1.192659475	-3.1328577	0.001731	0.038326
CALCB	8.742339926	-3.608172377	1.151796838	-3.1326465	0.001732	0.038326
RP11-76C10.3	5.489362486	5.328765009	1.700732443	3.13321771	0.001729	0.038326
PRELID2	13.42879981	2.689572437	0.859133093	3.13056552	0.001745	0.038555
PIK3R1	6454.893034	1.157111884	0.369733428	3.12958418	0.001751	0.038641
TMEM184B	272.253837	-1.157132715	0.36978242	-3.1292259	0.001753	0.038645
LINC01128	105.1081204	1.220035828	0.389980448	3.12845384	0.001757	0.038703
SLC38A4	23.52042114	-2.072995661	0.662778226	-3.1277365	0.001762	0.038755
TLE4	801.7712605	-0.863730976	0.276461666	-3.1242341	0.001783	0.039175
ZNF827	277.8891365	1.11499993	0.357057009	3.12275043	0.001792	0.03933
ZFP36	4477.077085	1.284591079	0.411491628	3.12179153	0.001798	0.039376
NEDD9	1548.861105	0.806224475	0.258260184	3.12175289	0.001798	0.039376
IFITM3	78.51859584	1.322939917	0.423843257	3.12129518	0.001801	0.039393
DENND1B	977.3729852	0.919409262	0.294688958	3.11993116	0.001809	0.039532
MT-ND5	413.6557266	1.565919571	0.502777008	3.11454093	0.001842	0.040217
RP11-739B23.1	21.56484392	2.303420993	0.741215163	3.107628	0.001886	0.041125
SMIM3	33.87935989	-2.049956885	0.661039522	-3.1011109	0.001928	0.041948
CSGALNACT1	143.3067423	-5.394137692	1.739284066	-3.1013552	0.001926	0.041948
CRBN	522.8506267	-0.949590549	0.306257082	-3.1006321	0.001931	0.04197

FAM131B	11.4411843	-3.27651585	1.057123807	-3.0994627	0.001939	0.042089
CTD-3060P21.1	28.83529106	-5.497248274	1.773844662	-3.0990584	0.001941	0.042101
CYB561	35.63793359	-1.859006659	0.600161579	-3.0975103	0.001952	0.042275
PCNT	1091.599795	-1.208460715	0.39033191	-3.0959824	0.001962	0.042447
GRAMD4	324.849983	-1.001288005	0.323465159	-3.095505	0.001965	0.042469
ITPRIP	1130.985764	-1.545490286	0.499527821	-3.0939023	0.001975	0.042652
GPD2	486.358469	0.860554497	0.278280836	3.09239583	0.001985	0.042822
PCDHGC5	9.180811127	-3.276099058	1.059932399	-3.0908566	0.001996	0.042998
CERS6	324.7523651	0.965755319	0.312511572	3.09030258	0.002	0.043031
ZNF331	1440.294709	1.489122058	0.482764456	3.08457269	0.002038	0.043821
PLS1	13.29165538	-2.787144227	0.90420335	-3.0824308	0.002053	0.044042
DTX3L	665.377439	-0.870825937	0.282485425	-3.0827287	0.002051	0.044042
SMS	89.55444368	1.172468232	0.380620185	3.08041527	0.002067	0.044293
RHBDF2	613.4739777	-1.311496692	0.425937411	-3.0790831	0.002076	0.044443
ZFR2	30.75157754	2.239980648	0.72776548	3.07788801	0.002085	0.044574
TNFRSF25	72.80745646	-1.402662287	0.455833632	-3.0771365	0.00209	0.044638
LTB	66.4150572	1.317363754	0.428375918	3.07525166	0.002103	0.044872
PLAC8	1296.456855	-1.03385581	0.336335095	-3.0738862	0.002113	0.04503
ATP1B3-AS1	71.53640746	1.712225765	0.557419457	3.07170075	0.002128	0.045312
NANOGP4	70.53895419	2.052926633	0.670057989	3.06380443	0.002185	0.046475
AC093609.1	7.51031913	-4.058882661	1.325298597	-3.0626175	0.002194	0.046609
CARMIL1	16.71258645	3.029983789	0.989630973	3.06173096	0.002201	0.046689
FAM20B	432.8781269	0.95680595	0.312532098	3.06146459	0.002203	0.046689
HEY1	5.69864969	-4.742500226	1.549284063	-3.0610915	0.002205	0.046697
NPM2	4.983667159	5.064897606	1.656285432	3.05798597	0.002228	0.047083
GPR65	872.8409152	-1.257048003	0.411045896	-3.0581695	0.002227	0.047083
AP001625.6	19.39014775	2.718629871	0.889283002	3.05710315	0.002235	0.047171
ZNF571	321.3222286	1.051819661	0.344095694	3.05676496	0.002237	0.047174
SLC7A1	73.98137676	-1.387171202	0.453989063	-3.0555168	0.002247	0.04732
RP11-452L6.1	14.2097723	2.337378449	0.765452426	3.05359075	0.002261	0.047574
UBAP1	331.5125991	0.921529008	0.301962477	3.05179974	0.002275	0.047739
AC108448.2	7.855711046	-3.833408092	1.256197402	-3.0515969	0.002276	0.047739
FRMD3	30.86060596	-1.757381724	0.575788235	-3.052132	0.002272	0.047739
CTD-2647E9.3	61.41096225	1.525211302	0.500392897	3.04802748	0.002303	0.048258
MEGF11	20.46406355	-2.590686577	0.850223205	-3.0470664	0.002311	0.048345
UROS	239.4280947	0.917315121	0.301069511	3.04685492	0.002312	0.048345
EMBP1	50.44107194	1.453678059	0.477751854	3.04274708	0.002344	0.048958
RP11-142O6.1	14.55477455	2.383088876	0.783868591	3.04016375	0.002364	0.049327
RN7SKP203	13.58008519	2.404689244	0.791142415	3.03951501	0.00237	0.049382
AC144831.1	86.75695521	-1.67418236	0.551137377	-3.0376861	0.002384	0.04963
NFKBID	273.1403373	0.862385145	0.444210329	1.94138922	0.052211	0.433943
P4HA2	18.12806004	1.573295205	0.810681712	1.94070642	0.052294	0.434266
TRAJ49	4.626969143	2.666037338	1.373674755	1.94080682	0.052282	0.434266
ACVR1B	53.50993702	0.82656901	0.426022762	1.94019917	0.052355	0.434412
ZFP37	19.31446283	-1.422736644	0.733239174	-1.9403446	0.052338	0.434412
TAS2R50	11.86368357	-1.694160342	0.873421326	-1.9396828	0.052418	0.43475
PRR29	54.38531259	-0.851275512	0.439143862	-1.9384889	0.052564	0.435505
HYDIN	3.453942895	-3.817808313	1.969491551	-1.9384741	0.052565	0.435505

PFAS	145.0420999	0.670323025	0.345813978	1.93839193	0.052575	0.435505
PPIF	221.887451	0.773899645	0.399430071	1.93750972	0.052683	0.436214
RN7SL328P	21.44405306	-1.392885419	0.719406897	-1.936158	0.052848	0.437277
CTB-55O6.13	34.37273922	1.124975994	0.581053249	1.93609793	0.052856	0.437277
OCEL1	42.51419531	-1.086555446	0.561330077	-1.9356801	0.052907	0.437306
RP11-374F3.5	29.88803182	1.302278741	0.672763988	1.93571411	0.052903	0.437306
CAMK4	22.41359402	-1.310236173	0.676940071	-1.9355276	0.052926	0.437306
SYTL1	963.7846097	-0.604050337	0.312145006	-1.9351594	0.052971	0.437419
ARHGEF37	9.676126326	1.723976476	0.89091874	1.93505468	0.052984	0.437419
RP13-870H17.3	3.548010395	3.888139955	2.011593739	1.93286541	0.053253	0.439458
DONSON	59.23926884	-0.830094037	0.429651193	-1.9320185	0.053357	0.440136
CTD-2538C1.2	7.623260516	1.977081124	1.023534732	1.93162094	0.053406	0.440357
TRIM39	182.5271884	0.739139035	0.382933731	1.93020091	0.053582	0.441621
HOXB3	61.9240084	-1.221785915	0.633156641	-1.929674	0.053647	0.441727
MTCO2P1	4.078188604	-3.147546534	1.631007151	-1.9298177	0.053629	0.441727
DUSP22	369.192734	-0.71360957	0.369830956	-1.9295561	0.053662	0.441727
ZNF233	39.1955695	0.97202908	0.503924363	1.92891861	0.053741	0.441825
ITGA2B	17.28484965	-1.433999691	0.743382441	-1.9290201	0.053728	0.441825
PGBD2	73.74587287	-0.805450272	0.417500169	-1.9292214	0.053703	0.441825
ZNF831	1446.229725	-0.555181255	0.287900447	-1.9283793	0.053808	0.442008
TESC	85.83687025	0.835469022	0.433234997	1.92844306	0.0538	0.442008
DHRS7	369.7358985	-0.631687282	0.327614732	-1.9281406	0.053838	0.442068
ATOX1	147.4383101	-0.66930123	0.347272541	-1.9273082	0.053941	0.442735
DPP9	652.7026466	0.590107933	0.306214631	1.92710561	0.053966	0.442758
PLP2	49.61765566	1.061312218	0.550948166	1.92633769	0.054062	0.44336
LINC00649	63.85216025	-1.060408799	0.550778737	-1.92529	0.054193	0.444248
URGCP	204.7058378	0.670738676	0.348419266	1.92509066	0.054218	0.444268
GABPB2	294.1844014	-0.586557862	0.304857149	-1.9240417	0.054349	0.444427
TAB1	192.2291424	0.634198466	0.329579475	1.92426566	0.054321	0.444427
ZNF132	49.62275308	0.887028675	0.46087564	1.92465949	0.054272	0.444427
RPIA	87.74437446	0.751223068	0.39044091	1.9240378	0.05435	0.444427
TECTA	9.348396048	-1.861007395	0.967050727	-1.9244155	0.054303	0.444427
ARL5B	533.2881163	0.659256127	0.342788148	1.92321739	0.054453	0.445084
FGFR1OP2	788.4098029	0.610001305	0.317240677	1.92283446	0.054501	0.445109
AGMAT	6.687693193	2.066722238	1.07477224	1.92293973	0.054488	0.445109
PRPF19	182.8866249	0.676642526	0.351975026	1.9224163	0.054553	0.445354
CAMSAP1	401.7006829	0.588308834	0.306167466	1.92152629	0.054665	0.446084
RPL41	315.4351713	0.639408984	0.332920098	1.92060794	0.054781	0.446844
C1QC	9.42604925	2.492265441	1.297837264	1.92032199	0.054817	0.446954
RP11-59C5.3	137.8217492	-0.699413322	0.36437356	-1.9194953	0.054922	0.447385
HIST1H2BE	77.37019216	-0.743840576	0.387631833	-1.9189357	0.054992	0.447385
ADM2	4.743368615	-2.71919215	1.417110284	-1.9188289	0.055006	0.447385
LUCAT1	21.28557051	-1.436127901	0.748183428	-1.9194864	0.054923	0.447385
SNORD12B	51.91001118	0.952053783	0.49602575	1.91936363	0.054938	0.447385
RP11-354B3.1	66.97453956	0.903313335	0.470680189	1.91916583	0.054963	0.447385
RP11-269F20.1	17.21995173	-1.380783953	0.719731083	-1.918472	0.055051	0.447568
MUM1	240.7787985	0.667415763	0.348037599	1.9176542	0.055155	0.447858
ZNF496	298.1036438	0.571527757	0.298012017	1.91780104	0.055136	0.447858

SCML1	29.54978599	1.080374329	0.563363628	1.91772112	0.055146	0.447858
C9orf147	5.879590635	-2.362803117	1.232498652	-1.9170837	0.055227	0.448262
ZC3H7A	394.3016994	0.566946007	0.295829878	1.91645959	0.055307	0.448722
KRT86	55.62069842	1.241449579	0.6479316	1.9160195	0.055363	0.448992
CYLD	2414.957684	0.50762085	0.264960994	1.91583237	0.055386	0.449001
AMZ2	578.1858276	0.531323412	0.277379059	1.91551379	0.055427	0.449145
NFIA	37.99974145	-1.001555314	0.523107411	-1.9146265	0.05554	0.449753
KIAA1524	68.7865632	0.880663802	0.45998035	1.91456831	0.055548	0.449753
RP11-852E15.1	19.00161043	1.28208268	0.669803033	1.91411895	0.055605	0.450033
PRTFDC1	9.462076756	1.699478203	0.888227699	1.91333619	0.055705	0.450474
NCOR2	386.726052	-0.736563981	0.384934889	-1.9134768	0.055687	0.450474
KIAA1324	12.88576917	-1.59618656	0.834525228	-1.9126882	0.055788	0.450776
CCNJL	282.5113472	0.86458807	0.452022362	1.91271084	0.055785	0.450776
RP11-466A19.1	4.190852553	3.035993777	1.587560416	1.91236425	0.055829	0.450781
DENND4B	835.6359998	-0.597371791	0.312379486	-1.9123272	0.055834	0.450781
AMPH	16.81129976	-1.391444574	0.72769127	-1.9121359	0.055859	0.450795
ZIK1	126.0853792	0.799434172	0.418172222	1.91173428	0.05591	0.451026
EIF5	3932.628359	0.586242308	0.306842513	1.91056416	0.056061	0.451827
HMGB1P8	51.80511501	1.058904693	0.554316274	1.91028974	0.056096	0.451827
IL17RE	3.665334064	-3.184058683	1.666827733	-1.9102506	0.056101	0.451827
FAM214B	119.7520743	-0.729035106	0.381609778	-1.9104204	0.056079	0.451827
ZNF334	13.19318616	1.673048521	0.875992637	1.90988879	0.056148	0.452017
DAPK1	66.03810005	-2.587510321	1.354957629	-1.9096614	0.056177	0.452069
CCSER1	6.242568636	-2.507191607	1.313209884	-1.9092086	0.056235	0.452174
GNB1L	56.43840431	-0.974185553	0.510257061	-1.9092054	0.056236	0.452174
SORBS1	16.52680402	-1.399012009	0.733032371	-1.9085269	0.056323	0.452399
MTCO1P11	15.20089241	-1.793023024	0.939515173	-1.9084556	0.056332	0.452399
SAMD9	1600.030052	-0.606933007	0.318011057	-1.908528	0.056323	0.452399
ZNF112	18.38278208	1.499253482	0.78582052	1.90788283	0.056406	0.452809
RIC8A	890.3032165	0.591882769	0.310338781	1.90721497	0.056493	0.453319
AP001469.5	18.16116118	-1.387615646	0.727705965	-1.9068356	0.056542	0.453529
SLCO2B1	3.339333401	3.414530586	1.791666818	1.90578435	0.056678	0.454438
TGFBR1	1188.394329	-0.699559786	0.36716045	-1.9053245	0.056738	0.454732
OPA3	150.7035702	0.629282832	0.330517426	1.90393239	0.056919	0.455998
RP11-322D14.1	7.78322969	-2.02877446	1.065712328	-1.9036793	0.056952	0.456077
NCAPH	15.69376622	-1.361956319	0.71565762	-1.9030837	0.05703	0.456514
FAM179A	486.0533995	0.615420811	0.32341538	1.90288047	0.057056	0.456542
RP11-484L8.1	13.89266982	-1.591942743	0.83736778	-1.9011273	0.057285	0.457814
RN7SL577P	14.1352038	1.563157618	0.822301183	1.90095509	0.057308	0.457814
TDGF1P6	42.0372725	1.265408815	0.66556104	1.90126636	0.057267	0.457814
RP11-693N9.2	133.2412741	0.817479355	0.429926045	1.9014418	0.057244	0.457814
AMY2B	74.38986116	-0.871147879	0.458343326	-1.9006448	0.057349	0.457954
EEF1AKMT1	18.18896923	1.277392769	0.672214531	1.90027545	0.057397	0.458155
COL4A3	14.89634123	1.422227149	0.748788854	1.89937008	0.057516	0.458919
PCM1	2814.429104	0.525463695	0.276982047	1.8971038	0.057814	0.461113
IQCC	51.24357102	0.868890256	0.458110852	1.89668123	0.05787	0.461186
MAP4K1	257.1215463	0.639877754	0.337344655	1.89680715	0.057853	0.461186
ACOT4	21.28223311	-1.326035745	0.699309163	-1.8962082	0.057933	0.461312

RP11-429G19.3	29.72978215	1.107103006	0.583836235	1.89625607	0.057926	0.461312
AP000708.1	13.62775412	1.497630109	0.789884553	1.89601139	0.057959	0.461333
AC093495.4	71.55381408	-0.775457415	0.409177165	-1.895163	0.058071	0.461835
RP11-620J15.3	60.91918444	0.996622224	0.525906879	1.89505455	0.058085	0.461835
IPCEF1	1113.410558	0.798747761	0.421501599	1.89500529	0.058092	0.461835
PEX11G	10.29281885	-1.704234761	0.899952684	-1.8936937	0.058266	0.463032
GALNT10	981.3596227	-0.498243692	0.263248774	-1.8926724	0.058401	0.463924
CTD-2651B20.7	131.3841882	1.930612773	1.020243927	1.89230509	0.05845	0.463939
REPIN1	359.1527498	0.601792742	0.31799396	1.89246595	0.058429	0.463939
SLC28A3	3.341834838	-3.654788019	1.932243762	-1.8914736	0.058561	0.464632
ICA1L	67.12649567	-0.925156363	0.489260029	-1.8909298	0.058634	0.465021
SCYL1	256.0349424	0.587066599	0.310693985	1.8895332	0.05882	0.465404
TBXA2R	3.990299153	-3.338048494	1.766619921	-1.8895114	0.058823	0.465404
BCAT2	36.88032229	0.968562558	0.512589829	1.88954697	0.058819	0.465404
ADCY10P1	54.94300157	-0.905833062	0.479182343	-1.8903724	0.058708	0.465404
ZFP1	68.55164536	-0.85994602	0.45497006	-1.8901156	0.058742	0.465404
RP11-524O1.4	5.299049904	-2.479994998	1.312324702	-1.8897724	0.058788	0.465404
NTHL1	20.29840011	-1.217047862	0.644344765	-1.8888147	0.058917	0.465956
RP11-16E23.3	87.62793651	1.17315012	0.621283375	1.88826897	0.05899	0.466161
RP11-431M7.3	10.3977706	-1.777629877	0.941373342	-1.8883367	0.058981	0.466161
MAP1A	17.95652382	-1.35535685	0.718063379	-1.887517	0.059091	0.466586
FERMT2	22.04595108	1.360497502	0.720752183	1.88760788	0.059079	0.466586
CYB561D1	165.6585322	-0.680535077	0.360764943	-1.886367	0.059246	0.467061
TACC3	153.3127354	0.720130446	0.381707262	1.886604	0.059214	0.467061
RNF145	424.8527895	0.644872375	0.341817648	1.88659766	0.059214	0.467061
OFD1	1057.372238	0.551469138	0.29231758	1.88654113	0.059222	0.467061
PRDX1	58.15303863	-0.862132922	0.45711405	-1.8860346	0.05929	0.467227
SH3BP4	5.819837842	-2.365076149	1.254472935	-1.8853146	0.059387	0.467619
WNT3	32.13018603	1.232866546	0.653879765	1.88546368	0.059367	0.467619
HNRNPC	2375.534699	0.519308149	0.275589636	1.88435297	0.059517	0.468455
RP11-51J9.5	90.51210777	0.802502806	0.426090673	1.88340853	0.059645	0.469274
FAM124B	26.01215872	-1.337666048	0.710503691	-1.8827011	0.059741	0.469841
ADGRF3	28.73679937	-1.097973334	0.583387446	-1.8820654	0.059827	0.470332
ZNF256	175.0038013	0.824708197	0.438271728	1.88172803	0.059873	0.470505
CTD-2410N18.4	19.75745489	1.22841833	0.653285228	1.88037059	0.060058	0.471768
CHD4	2151.680599	0.507727317	0.270150951	1.8794208	0.060187	0.472021
RNU5E-4P	21.80999052	1.610682448	0.857509228	1.87832666	0.060336	0.472021
JPH3	25.41384185	-1.269924863	0.675837663	-1.8790383	0.060239	0.472021
TATDN1P1	28.4642852	1.238244185	0.659268409	1.87820949	0.060353	0.472021
RP11-505P4.6	3.683919816	3.097999082	1.648585481	1.8791862	0.060219	0.472021
RP11-478C1.8	6.000153593	-2.245727617	1.19553338	-1.8784315	0.060322	0.472021
SS18L1	96.61166084	-0.717654875	0.382061163	-1.8783769	0.06033	0.472021
SH3PXD2A	187.46849	0.698229719	0.37174882	1.87822982	0.06035	0.472021
RNU6ATAC	23.15955735	1.385199205	0.737151972	1.87912297	0.060228	0.472021
RP11-79M19.2	26.75352549	-1.376523463	0.732772787	-1.8785133	0.060311	0.472021
RN7SKP266	3.670510361	2.862205482	1.523691098	1.87846834	0.060317	0.472021
DECR1	255.8672175	-0.669304695	0.356428873	-1.8778072	0.060408	0.472264
ITGAV	256.7548292	-0.662541976	0.352914046	-1.8773466	0.060471	0.472398

RP11-531A24.5	34.49513545	-1.211225242	0.645187507	-1.8773228	0.060474	0.472398
EHBP1L1	822.2575848	0.555545704	0.29595034	1.87715853	0.060496	0.472398
DPEP2	162.1439054	-0.790708723	0.421408898	-1.8763456	0.060608	0.473081
CYB561A3	124.3583186	-0.648781141	0.345867625	-1.8758077	0.060682	0.473097
ANKRD28	1274.700928	0.509393398	0.271529639	1.87601398	0.060653	0.473097
ORAI1	269.5273765	-0.926789185	0.494055796	-1.8758796	0.060672	0.473097
LINC01278	95.29462978	-0.861680233	0.459460433	-1.8754177	0.060735	0.473328
ASPM	30.5271422	-1.236985584	0.659659673	-1.8751875	0.060767	0.473388
TRGV5	32.36612656	-1.074562155	0.573099603	-1.8750007	0.060793	0.473401
H2AFJ	130.1662953	0.637629605	0.340222625	1.87415403	0.060909	0.473935
TNFRSF14	708.4929142	-0.684827117	0.36538798	-1.8742464	0.060896	0.473935
ATP11A-AS1	7.632097275	-2.162513497	1.153997256	-1.873933	0.06094	0.473986
STAB1	68.20762191	0.982351084	0.524547608	1.87275868	0.061102	0.474892
ATP5HP4	125.0157257	-0.769955431	0.411204992	-1.872437	0.061146	0.474892
PGM2L1	81.85160889	-0.728148887	0.389031256	-1.8716976	0.061248	0.474892
FDPSP3	76.88641007	0.820054365	0.438087342	1.87189696	0.061221	0.474892
RGP1	227.3316621	0.615683618	0.328920991	1.87182829	0.06123	0.474892
CACNB1	98.70030245	0.683599605	0.36518053	1.87194976	0.061214	0.474892
RP11-567P19.1	13.29784178	-1.627816442	0.86949451	-1.8721411	0.061187	0.474892
RNASEH2B	304.3966134	-0.6036685	0.322411704	-1.8723529	0.061158	0.474892
TIMP1	34.42551043	-1.402287888	0.749289377	-1.8714904	0.061277	0.474928
TRAF2	181.9688981	0.704409239	0.376707064	1.86991248	0.061496	0.476071
USP20	619.3825661	0.651319274	0.348316652	1.86990565	0.061497	0.476071
ST7-OT4	18.56209682	-1.504673305	0.804614358	-1.8700553	0.061476	0.476071
ATXN10	348.5202799	0.561392907	0.300311554	1.86936833	0.061572	0.476462
RP11-410N8.3	15.71127087	-1.59094379	0.851178016	-1.8691082	0.061608	0.476556
MAPRE2	1370.539856	-0.519325214	0.277953025	-1.868392	0.061707	0.477087
UBXN2A	168.0612601	0.638099904	0.341546253	1.86826791	0.061725	0.477087
CTB-39G8.3	19.8636892	-1.216218921	0.651394457	-1.8671005	0.061888	0.478158
MRPL57P8	6.817928637	-2.196744942	1.176748087	-1.8667929	0.061931	0.478303
SYK	1389.857232	0.551030081	0.295242297	1.86636565	0.06199	0.478577
CASP3	528.5585715	0.575327116	0.30840373	1.86549986	0.062111	0.478764
WDR13	187.5373027	-0.654327674	0.350684746	-1.8658572	0.062061	0.478764
MIR23A	49.82198227	1.076221851	0.576855851	1.8656686	0.062088	0.478764
ICOS	7.197018146	-2.186364355	1.171827205	-1.8657737	0.062073	0.478764
RAB3IP	77.5676679	0.800903552	0.429476377	1.86483726	0.062204	0.479293
Sep-08	25.4464822	-1.178981842	0.632409279	-1.8642703	0.062284	0.479713
ESD	89.50184971	0.801750502	0.430100005	1.86410252	0.062307	0.479713
TMSB10	2095.195347	-0.62174917	0.333702826	-1.8631822	0.062437	0.480521
ARHGAP18	345.479762	0.678025375	0.364007664	1.86266786	0.062509	0.480891
ICAM2	191.3989294	-0.618661063	0.33216887	-1.8624896	0.062534	0.480897
DST	325.3436659	0.593188238	0.318589372	1.86192099	0.062614	0.481138
AC010150.1	4.251390531	-2.86257991	1.53734211	-1.8620318	0.062599	0.481138
GTF2IP13	12.0359335	-1.55273309	0.834085695	-1.861599	0.06266	0.4813
PPP1R15A	5214.005352	0.624058542	0.335331824	1.86101795	0.062742	0.481556
SEC14L2	106.7394833	0.802616081	0.431255981	1.86111293	0.062728	0.481556
LY75	215.1384347	-0.6832609	0.367194426	-1.8607605	0.062778	0.481647
FKBP5	1149.004031	-1.597499364	0.858776915	-1.860203	0.062857	0.482065

CTHRC1	9.610325863	-1.742450257	0.93684911	-1.8599049	0.062899	0.482093
PRR13P1	9.214503977	1.696923736	0.912406986	1.85983203	0.062909	0.482093
AXL	37.3421755	0.942843935	0.507004119	1.85963762	0.062937	0.482117
SFT2D2	1115.353316	-0.482689314	0.259673492	-1.8588317	0.063051	0.482804
RP5-1031J8.1	3.46173959	-3.130313185	1.684575329	-1.858221	0.063138	0.482913
SPATA1	11.65659933	-1.627709909	0.875953448	-1.8582151	0.063138	0.482913
RP11-324I22.3	19.35840393	-1.635499001	0.880063857	-1.8583867	0.063114	0.482913
PBXIP1	1459.520539	0.5968274	0.321339676	1.85731002	0.063267	0.483335
RP11-582J16.4	68.63804346	-0.769996563	0.414545467	-1.8574478	0.063247	0.483335
ECE1	163.9802728	-0.65215177	0.35107025	-1.8576105	0.063224	0.483335
GCDH	198.3797173	0.804989229	0.433545114	1.85676001	0.063345	0.483746
DHRS4L1	4.533792087	-2.78552822	1.500958376	-1.8558331	0.063477	0.484567
DDX24	1819.790018	0.529724717	0.28546917	1.85562846	0.063507	0.484602
RIMKLB	139.6516901	0.749999064	0.404288681	1.85510775	0.063581	0.484982
SUCLG2-AS1	36.02021591	1.004827195	0.541929464	1.85416602	0.063715	0.485821
TEPSIN	182.2640846	-0.655655333	0.353765848	-1.8533596	0.063831	0.486513
RAB27A	672.3339772	0.556371119	0.300227973	1.85316216	0.063859	0.486541
NUF2	14.2218232	-1.489930661	0.804237275	-1.8526009	0.06394	0.486638
EFCC1	6.270391997	2.226309857	1.201749025	1.85255807	0.063946	0.486638
DHODH	67.88765981	0.835622455	0.451029556	1.85269999	0.063925	0.486638
HIST1H3G	24.90302316	-1.274644009	0.688257425	-1.8519873	0.064028	0.487074
HSPA9	1625.041677	0.541163947	0.292352429	1.85106705	0.06416	0.487892
GALM	101.1084547	-0.72057011	0.389451029	-1.8502201	0.064282	0.488068
NPM1P26	40.63045654	1.069140884	0.57782457	1.8502863	0.064272	0.488068
MIR4496	9.668842108	-1.988496751	1.074585557	-1.8504778	0.064245	0.488068
TWF2	188.0404323	-0.666433218	0.360136111	-1.8505037	0.064241	0.488068
CD3D	20.21489821	-1.236627681	0.66848464	-1.8498969	0.064328	0.488234
SAMD3	1001.638499	-0.556668988	0.300991084	-1.8494534	0.064392	0.488531
ZNF69	20.12909162	-1.234385959	0.66757436	-1.8490614	0.064449	0.488773
RP11-358B23.5	18.70436541	-1.29414238	0.700435228	-1.8476261	0.064656	0.490158
TTC30B	70.88492003	0.911034353	0.493138489	1.8474209	0.064686	0.490196
RNU6-407P	6.400056754	2.391547734	1.29518238	1.84649496	0.06482	0.490672
H6PD	764.4051109	0.487220964	0.263828126	1.84673625	0.064785	0.490672
SYCP2	81.06938436	-0.762887849	0.413159412	-1.8464734	0.064824	0.490672
STRIP2	13.61180436	-1.854558931	1.004509081	-1.8462341	0.064858	0.490746
RP11-930O11.2	3.163430806	-3.085312513	1.671506454	-1.8458275	0.064917	0.490817
SLC20A1	1811.624609	-0.499688361	0.27069486	-1.845947	0.0649	0.490817
MMD	166.2444128	0.624115741	0.338180895	1.84550857	0.064964	0.49098
EI24	148.7293592	0.648012228	0.351237746	1.84493903	0.065046	0.491418
PIK3R6	17.09444094	1.493229959	0.809608383	1.84438549	0.065127	0.491838
RP11-23J18.1	34.70088406	-0.946536849	0.513299943	-1.8440229	0.06518	0.492049
RNY1P16	4.30007338	3.210941411	1.742858778	1.8423417	0.065425	0.492186
RP11-774O3.3	14.20992442	-1.472777245	0.799371093	-1.8424199	0.065414	0.492186
TRAM2	209.0170036	-0.591736525	0.321087782	-1.842912	0.065342	0.492186
BTN3A2	1729.224757	-0.535037147	0.290435145	-1.8421915	0.065447	0.492186
SEC24C	656.2097416	0.55357987	0.3003057	1.84338782	0.065272	0.492186
AC094019.4	18.92098521	1.316042729	0.713979191	1.84325082	0.065292	0.492186
RP11-521B24.4	23.5168699	1.258032443	0.682882153	1.84223945	0.06544	0.492186

RP11-236P24.3	4.674432523	-2.544155818	1.38038597	-1.8430757	0.065318	0.492186
LRRC75B	23.44039408	1.152476095	0.625412842	1.84274453	0.065366	0.492186
IFI30	8.908716041	-1.755023493	0.952596831	-1.8423571	0.065423	0.492186
CDCA3	15.23044425	-1.426690829	0.774804948	-1.8413548	0.06557	0.492842
ACACA	360.9075499	0.543303308	0.295072225	1.84125533	0.065584	0.492842
IGFBP3	16.21532595	-1.739147908	0.944656896	-1.8410366	0.065616	0.492895
SLC2A13	198.4758165	0.605389856	0.328864798	1.84084724	0.065644	0.492916
DDX3X	6851.474944	0.572088097	0.310816183	1.84059945	0.06568	0.492996
TRBV25-1	10.17542087	1.70001874	0.923705314	1.84043408	0.065705	0.492996
TRBJ1-2	9.726494571	1.619245587	0.880040745	1.83996661	0.065773	0.493324
PGM2	336.4868878	0.671319146	0.36497973	1.83933268	0.065866	0.493835
CST7	2027.756161	-0.554488977	0.301516506	-1.8390004	0.065915	0.494013
KCNJ1	19.94782358	-1.223258547	0.665281511	-1.8387082	0.065958	0.494148
STX1A	66.06679641	0.914521601	0.497557647	1.83802139	0.066059	0.494441
GGACT	37.41296899	-0.980502827	0.533406012	-1.8381923	0.066034	0.494441
RNF32	16.89935643	-1.447681478	0.787668299	-1.8379329	0.066072	0.494441
RP11-535C21.3	8.362917444	-1.863600768	1.014207978	-1.8374937	0.066137	0.494738
AL109767.1	8.184456432	-1.91400324	1.041860273	-1.8371017	0.066195	0.494796
TXLNB	22.30720342	-1.224863128	0.666707068	-1.8371834	0.066183	0.494796
RP11-173M1.4	4.174879966	-2.840265083	1.547034898	-1.8359412	0.066366	0.49589
SWT1	95.5571018	-0.676556346	0.368742015	-1.8347688	0.06654	0.496982
SLCO4A1	15.51145057	1.310458881	0.714296413	1.83461495	0.066563	0.496982
PFN2	17.94644525	-1.389281956	0.757383606	-1.8343174	0.066607	0.497123
RP11-517I3.1	25.21649577	1.202857992	0.65588626	1.83394296	0.066662	0.497163
EIF2AK2	553.0946383	0.566163968	0.30869784	1.8340393	0.066648	0.497163
TRMT5	31.40614374	-1.15703571	0.631051711	-1.8335038	0.066728	0.497461
AGAP3	221.6292908	0.657461551	0.358627699	1.83327042	0.066762	0.497479
TOB1-AS1	14.42092787	-1.714290078	0.935161515	-1.8331487	0.06678	0.497479
CACNB3	68.02538305	0.812040045	0.443080031	1.83271641	0.066845	0.49777
ITPR3	1084.753715	0.606321888	0.330903285	1.83232357	0.066903	0.497831
FANCA	128.2469693	-0.915410046	0.499572837	-1.8323855	0.066894	0.497831
VARS	200.0415941	0.645629516	0.352388814	1.83215099	0.066929	0.497834
CPVL	70.63155206	0.761991606	0.415982618	1.83178713	0.066983	0.497863
DNAJA1	1893.343451	0.558719035	0.304986501	1.83194677	0.066959	0.497863
RP5-1142A6.9	23.46796222	-1.164983913	0.636069398	-1.8315359	0.067021	0.497954
TBC1D22A	268.081559	-0.5980256	0.326609582	-1.8310106	0.067099	0.498337
SALL2	10.89931819	-1.787578633	0.976454389	-1.8306832	0.067148	0.498337
KLRF2	7.025150892	2.188250115	1.195217118	1.830839	0.067125	0.498337
FBXO3	694.4399442	-0.495616589	0.270856217	-1.8298143	0.067278	0.499113
